# Supplementary material for: Activation by cleavage of the epithelial Na+ channel α and γ subunits independently coevolved with the vertebrate terrestrial migration
Source: eLife. 2022 Jan 5;11:e75796. doi: 10.7554/eLife.75796 (PMC8791634; doi:10.7554/eLife.75796)
Supplement: Figure 1—source data 1. — Residues are colored by domain, as in Figure 1: transmembrane and intracellular domains are blue, palm and β-ball domains are orange, finger domain is light green, GRIP (gating release of inhibition by proteolysis) domain is dark green, thumb domain is yellow-green, and the knuckle domain is brown. GRIP domain polybasic tracts and C-terminal PY motifs are underlined in red. Select conserved residues in epithelial Na+ channel (ENaC) subunits are bold. [file elife-75796-fig1-data1.pdf]

Spotted Gar γ-like	--MVISKKA------------------
Asian Arowana γ-like	---MPPKK---------------
Coelacanth ASIC1	-------------------MAAPCHSSDSV----------LSPYDSKDRE---------------
Catfish ASIC1	-------------------MVTLTTTREPV----------NNGSKAPKAG---------------
E. Lamprey ASIC1	--MDLKGAPSDE---------------
E. Shark ASIC1	--MDLKPPAE---------------
J. Medaka ASIC1	----------------------MDLKADSD----------EMDYKRPAPI---------------
Black Rock Cod ASIC1	----------------------MDLKADSE----------DMDYKRPAPI---------------
Lancelet γ-like	--MS---------------
Lancelet α-like	--MPRT---------------
E. Shark α	---
Frog δ	---------------------------MES----------TEKEKKEGLI---------------
Coelacanth α	---------------------------MSE----------KKEEKSKGLI---------------
S. Lamprey α	---------------------PPTVLEFWR----------------GSVT---------------
J. Lamprey α	---
W. Lungfish α	----------------------MPNKEENA----------ENGKKKEGLF---------------
A. Lungfish α	----------------------MTDKEEEA----------EGGKKKEPMI---------------
Ropefish α	-------------------------MSTTD----------DKQERKEGLL---------------
Frog α	---------------------------MTK----------EEKNEKEALI---------------
Salamander α	-----------------------MTEE---------------KKEKEGLI---------------
Cow α	---------------------MKGDKPEEPGPGPEPSGPPPPTEEEEALL---------------
Human α	-MEGNKLEEQDSSPPQSTPGLMKGNKREEQGLGPEPAAPQQPTAEEEALI---------------
Chicken α	-MGTASRGGSVKAEKMPEGEKTRQCKQETE--------QQQKEDEREGLI---------------
Turtle α	----MHQVVAVKAENVPVGERLRRCKQEAEKQQKVEEVAEKLEKECQGLI---------------
Cow δ	--------MENGRLMAQVGRPGWGQAKWWAGAPLSLTSQMQAEGTGQTVGGGPGTWTCPQASPPT
Human δ	MAEHRSMDGRMEAATRGGSHLQAAAQTPPRPGPPSAPPPPPKEGHQEGLV---------------
Coelacanth δ	-----------------------CLQSLKM---------AQEEDKEEAVI---------------
Chicken δ	-----------------------MEQEAAR----------EEEERKEGLI---------------
Turtle δ	------------------------MEQEWV----------NEEMGDEGLI---------------
S. Lamprey β	----------------------MKIRKYLT----------RSLHRLQ-KG---------------
J. Lamprey β	-------------------MANMKIRKYLT----------RSLHRLQ-KG---------------
E. Shark β	-------------------MLWVSARRCLS----------QALRRLQ-DG---------------
Coelacanth β	----------------------MSVRKYFT----------RALHRLQ-KG---------------
Ropefish β	----------------------MGVQKYLT----------YALHRIQ-KG---------------
W. Lungfish β	---------------------MSFLKRFCV----------RSWHRIK-KG---------------
A. Lungfish β	----------------------MFLKRWFI----------RALHRLQ-KG---------------
Frog β	----------------MIHGKMKRLKRYFT----------RALHRIQ-KG---------------
Cow β	----------------------MHVKKYLL----------KGLHRLQ-KG---------------
Human β	----------------------MHVKKYLL----------KGLHRLQ-KG---------------
Chicken β	----------------------MNLKRYFV----------RALHRLQ-KG---------------
Turtle β	----------------MFTGMTMNFKRYFI----------RVLHRLQ-KG---------------
S. Lamprey γ	------------------MASEGDSKRVLH----------RVKDTLKIDG---------------
J. Lamprey γ	-----------------MASEGDSNKRVLH----------RVKDTLKIEG---------------
Ropefish γ	--------------------MESVAKKLPK----------KVKEKLPVTG---------------
E. Shark γ	--------------MESVGAMEPGKRKLSA----------KIMEKLPVTG---------------
W. Lungfish γ	---------------------MKNTKKFKE----------SVKKQLPLTG---------------
A. Lungfish γ	---------------------MGHGRRISE----------SIKKQLPVTG---------------
Coelacanth γ	------------------ATMTSRKKSLPE----------KIKENLPVTG---------------
Frog γ	--------------------MSKSGKKLTQ----------KLKKNLPVTG---------------
Cow γ	---------------------MAPGEKIKA----------KIKKNLPVTG---------------
Human γ	---------------------MAPGEKIKA----------KIKKNLPVTG---------------
Chicken γ	----------------------MAPGKITA----------RIKKTLPVRG---------------
Turtle γ	--------MEPPRPGDTRELNMAPGKTIKA----------KIKKTLPVTG---------------

Spotted Gar γ-like	---------------TLQSICRETLIHTSA**HG**VSSILRS-RSNHQKNCWIIFVVVVVGCML-**W**QC
Asian Arowana γ-like	-----------PGAMTSLWMLRDDIQHTTA**HG**IPNIFRA-RHWFRRSLWAMFVIFAFCCAI-**W**QC
Coelacanth ASIC1	-----------KRNHSLKQITIAFVKNSKF**HG**IRYIFAYHISKQRRAIWFLAFFIATGLLAI**W**SL
Catfish ASIC1	----------FERMSSMAKITLAFVFRTKV**HG**LRYVFAADKSKPRRFFWLVAICVCLALLFI**W**SC
E. Lamprey ASIC1	-----------SLDQARPSSVATFADSCTL**HG**IRHIFSPGGLSVRRLLWLLAFLGSLSLLV-**L**QS
E. Shark ASIC1	-----------DGVSNHPASVEAFAKTSTL**HG**ISHIFTYERFSFKRIIWTLAFLGSLSFLV-**H**TC
J. Medaka ASIC1	---------------------EVFATRSTL**HG**ISHMFTYERMCLKRTLWILFFMLSVGVLV-**M**VC
Black Rock Cod ASIC1	---------------------EVFASRSTL**HG**ISHMFTYERMCIKRTLWILFFLSSVGVLV-**M**VC
Lancelet γ-like	-----------EKRPSVRSTLRKYGENTSA**HG**IPRAVTT-KSLPRRLFWTCLFLASFSYFL-**Y**QA
Lancelet α-like	-----------TDNKVASALLE-FADTTTT**HG**VPRAVGS-SSLLRKICWTVAFVASLGYFL-**Y**QA
E. Shark α	--------------MDLARALALSRREAIC**HP**APRKP-------------------------**W**R-
Frog δ	-----------EFYDSFEDMLTFFCDNTTI**HG**TVRLNCSRKNKMKTTFWLVLYFVSFAMMY-**W**QF
Coelacanth α	-----------EFYSSYSDLFQFFCSTTTI**HG**AIRLVCTERNKMKTAFWSMLFVASFGLMY-**W**QF
S. Lamprey α	-----------DFYDSYDEMFEFFCDNTTI**HG**TIRLVCSKRNKLKTAFWSLLFTVTVILFY-**Y**TS
J. Lamprey α	-------------------MFEFFCDNTTI**HG**AIRLVCSKRNKLKTAFWSLLFIVTVILFY-**Y**TS
W. Lungfish α	-----------EFYDSFQELFEFFCINTTI**HG**TIRMVCSKHNNMKTAFWTILFIATFGIMY-**W**QF
A. Lungfish α	-----------GFYDSYQELFEFFCNNTTI**HG**TIRMVCSKHNNMKTVSWTILFITTFGVMY-**W**QF
Ropefish α	-----------EFYTSYSDLFNFFCSNTTI**HG**AIRLVCSENNRMKTAFWAILFPGTVAILY-**W**QF
Frog α	-----------EFFSSYRELFEFFCSNTTI**HG**AIRLVCSRRNRMKTAFWLVLFLVTFGLMY-**W**QF
Salamander α	-----------EFYSSYRELFEFFCNNTTI**HG**AIRLVCSAHNRMKTAFWVVLFIASFGLLY-**W**QF
Cow α	-----------EFHRSYRELFEFFCNNTTI**HG**AIRLVCSQHNRMKTVFWAVLWLCTFGMMY-**W**QF
Human α	-----------EFHRSYRELFEFFCNNTTI**HG**AIRLVCSQHNRMKTAFWAVLWLCTFGMMY-**W**QF
Chicken α	-----------EFYGSYQDVFQFFCSNTTI**HG**AIRLVCSKKNKMKTAFWSVLFILTFGLMY-**W**QF
Turtle α	-----------EFHKSYHELFQFFCNNTTI**HG**AIRLVCSKRNKMKTAFWSVLFFLTFGLMY-**W**QF
Cow δ	LPEEEHGERLVELHASFRELVTFFCTNSTI**HG**TIRLVCSSQNRLKTASWGLLLAGALGVLY-**W**QF
Human δ	-----------ELPASFRELLTFFCTNATI**HG**AIRLVCSRGNRLKTTSWGLLSLGALVALC-**W**QL
Coelacanth δ	-----------EFYDSFKDLFQFFCAHTTV**HG**GIRLICSERNMMKTAFWIILFFASFGLMY-**W**QF
Chicken δ	-----------EFYDSFKDMFEFFCKNTTI**HG**TIRLVCSSSNKMKTAFWTLLLLASFGMLY-**W**QF
Turtle δ	-----------EFYSSFKDMFEFFCKNTTI**HG**TVRLVCSSSNRMKTAFWTLLFLASFGMLY-**W**QF
S. Lamprey β	-----------PVA-SVSELLYWYCMNTNT**HG**CKRIVVY--GKKKRVLWFLITIIMLGVVL-**W**QW
J. Lamprey β	-----------PVA-SVSELLYWYCMNTNT**HG**CKRVVVY--GKKKRVLWFLITIIMLGVVL-**W**QW
E. Shark β	-----------PGE-SYRELLVWYCETTST**HG**PKRILTE--GPKKRALWLLLTLLLGGVVC-**W**QW
Coelacanth β	-----------PGY-TYKELLVWYCDNTNT**HG**PKRIIKE--GPKKQVLWFILTLTFTALIF-**W**QW
Ropefish β	-----------PGY-TYKELLVWYCNNTNT**HG**PKRIVTE--GPKKRFLWFLFTLVFAALVF-**W**QW
W. Lungfish β	-----------PGY-GYAELFHWYCDNTNT**HG**PKRLIIE--GPKKKAMWGLLTITFACLVF-**W**NW
A. Lungfish β	-----------PGY-GYSELFVWYCNNTNT**HG**PKRLIIE--GPKKKTLWSLFTVTFACLVF-**W**QW
Frog β	-----------PGY-TYKELLVWFCDNTNT**HG**PKRIIKE--GPKKRVMWFILTLVFAGLVF-**W**QW
Cow β	-----------PGY-TYKELLVWYCDNTNT**HG**PKRIICE--GPKKKAMWFVLTLLFTSLVC-**W**QW
Human β	-----------PGY-TYKELLVWYCDNTNT**HG**PKRIICE--GPKKKAMWFLLTLLFAALVC-**W**QW
Chicken β	-----------PGY-TYKELLVWYCDNTNT**HG**PKRIIKE--GPKKKVMWFFLTLLFASLVF-**W**QW
Turtle β	-----------PGY-TYKELLVWYCDNTNT**HG**PKRIIRE--GPKKKVIWFFLTLLFASLVF-**W**QW
S. Lamprey γ	-----------PDP-SITDLLDFYLNNTNM**HG**MRRIAVS-KGPIKKTIWIVFSLIAVAMVF-**W**QG
J. Lamprey γ	-----------PDP-SITDLLDFYLNNTNM**HG**MRRIAVS-KGPIKKTIWIVFSLIAVAMVF-**W**QG
Ropefish γ	-----------PYAITVKELMVWYCNYTNT**HG**CRRIVVS-RGRLRRWIWTVLTLSAVALIS-**W**QC
E. Shark γ	-----------PQALSMSELARWYCYNTNT**HG**FRRIVVS-RGRLRRGAWVLLTGCAASLIV-**W**QC
W. Lungfish γ	-----------PESRTVKDLMDWYCNNTNT**HG**CRRIAVS-RGHLRRWIWICFTLTAVAIIF-**W**QY
A. Lungfish γ	-----------PEAPTVKNLMDWYLNNTNT**HG**CRRIAVS-RGYLRRWIWICFTVSSVGMIF-**W**QW
Coelacanth γ	-----------PQALSISELMRWYCYNTNT**HG**CLRIVAS-RGRLRRWIWILLTLSAVALIF-**W**QC
Frog γ	-----------PQAPTLYELMQWYCLNTNT**HG**CRRIVVS-KGRLRRWIWISLTLCAVAVIF-**W**QC
Cow γ	-----------PQAPNIKELMQWYCLNTNT**HG**CRRIVVS-RGRLRRLLWILFTLTAVALIF-**W**QC
Human γ	-----------PQAPTIKELMRWYCLNTNT**HG**CRRIVVS-RGRLRRLLWIGFTLTAVALIL-**W**QC
Chicken γ	-----------PQAPTLRELMRWYCLNTNT**HG**CRRIVVS-RGRLRRFIWILLTLSAVGLIL-**W**QC
Turtle γ	-----------PQAPTVSELMHWYCMNTNT**HG**CRRIVVS-RGRLRRFIWILLTLSAVGLIL-**W**QC

Spotted Gar γ-like	SELINTFFHYPSQEKVTLVNSARLK-FPAVTFCNLNQVRKSLMLSKFSFLK-------GGLYFLN
Asian Arowana γ-like	MEIIMTFYSYPSHEKIRLISDTKLM-FPAVTICNLNSVRHSALKRNFALNNTFLDFCL-------
Coelacanth ASIC1	NRILYLFS-YPAVIKMQMIWARHLY-YPTVTICNYNLFRLSRMTK-------------ADLYYSG
Catfish ASIC1	NRLL-YLLSFPAVTKIYMVWANNMT-FPAVTLCNQNLFRVSSLTK-------------ADLYHSG
E. Lamprey ASIC1	LDWVQYYLRYPCVTKQDEVSTPLTV-FPAVTLCNLNEFRFSRMTR-------------NDLYHAG
E. Shark ASIC1	TQRIQYYFQYPHVTKLDEISAANMT-FPAITICNLNEFRFSKITK-------------NDLYHAG
J. Medaka ASIC1	VDRVQFYFEYPHVTKLDEVAASMIV-FPAITFCNLNSFRFSRVTR-------------NDLYHAG
Black Rock Cod ASIC1	VDRVQLYFQYPHVTKLDEVSAPMMV-FPSVTFCNLNSFRFSRVTR-------------NDLYHAG
Lancelet γ-like	QTLVNKYLVYPVNTDVK-IEWSELE-FPAVTICNANPLRYRELIKR----------------GSA
Lancelet α-like	SMLFNKYFDYPVATDIS-IKFATIE-FPAVTICNLNPVRLSKLNTAGGEFSSY---IISDVGTTA
E. Shark α	---------FPVKSHRCPPSFPLLLSLPPSPSPSPSPFTHSP-----------------------
Frog δ	GQLTDQYWAYPTSTIIG-LQSKGKI-FPAVTICNLNPYRFDQVNMYINQLDQLANETLYSLYEYR
Coelacanth α	GIIFGHYFSYPVSMSLT-LEHKKLL-FPAVTVCTLNPYRYKEVESELKELDSLTADTLFELYRYN
S. Lamprey α	ALVFLQYYSYTVAVTMG-LMFQQST-FPAITVCSLNPYRYEVVQSSLSQLDSMTGQALQQLYGYQ
J. Lamprey α	ALVFLQYYSYTVAVTMG-LMFQQST-FPAITVCSLNPYRYEAVQSSLRELDSMTGQALHRLYGYQ
W. Lungfish α	GLLLDQYYSFPVSITMA-VNYDKLV-FPAVTVCTLNPYRYNAVSTELANLDCYTEQLLSTLYHYT
A. Lungfish α	GLLLGQYYSYPVSITMS-VNFDKLI-FPAVTVCTLNPYRYNVVSTQLANLDCYTEELLSTLYHYN
Ropefish α	GLLFGQYFSHPVSIGVS-VNFNELQ-FPSVTVCTLNPYRYSAVREELKELDAVTEETLYKLYGYT
Frog α	GLLFGQYFSYPVSINLN-VNSDKLP-FPAVTVCTLNPYRYKAIQNDLQELDKETQRTLYELYKYN
Salamander α	GLLFGQYFSYPVSINMN-VNSDKLL-FPAVTVCTLNPYRYTAVLEDLRELDRLTEQTLYDLYRYN
Cow α	GQLFGEYFSYPVSLNIN-LNSDKLV-FPAVSICTLNPYRYKEIQEELEELDRITEQTLFDLYKYN
Human α	GLLFGEYFSYPVSLNIN-LNSDKLV-FPAVTICTLNPYRYPEIKEELEELDRITEQTLFDLYKYS
Chicken α	GILYREYFSYPVNLNLN-LNSDRLT-FPAVTLCTLNPYRYSAIRKKLDELDQITHQTLLDLYDYN
Turtle α	GILYRQYFSFPVNLNLN-LNSDRLT-FPAVTLCTLNPYRYSAVQKELDELDRITHQTLMDLYNYN
Cow δ	ALLFEQYWRYPVIMTVS-VHSERKL-FPSVTLCDMNPHRPHLARHHLRVLDDFARESIYSLYRFN
Human δ	GLLFERHWHRPVLMAVS-VHSERKL-LPLVTLCDGNPRRPSPVLRHLELLDEFARENIDSLYNVN
Coelacanth δ	GLLFSQYWGYPVSVAIR-VHSGPKI-FPAVTVCTLNPYRYTQVHKYLKELDQMALEVLSTWYGFN
Chicken δ	ALMFSQYWDYPVVLTMS-MHSEPKM-FPAITICNLDPYRFDLVSEHLAQLDRMAEKSVTVLYGIN
Turtle δ	ALLFSQYWTYPVIMTMS-VHSEPKM-FPAITLCNLDPYRFDLVSERLAQLDQMAEEAIANLYGYK
S. Lamprey β	VLLFQAYLSYGVSVSVN-MGFQRMN-FPAVTVCNLNAYRYSSMKDKIKDLEAYTRVALQTLYNYT
J. Lamprey β	VLLFQAYLSYGVSVSVN-MGFQRMN-FPAVTVCNLNAYRYSSIKDKIKDLEAYTRVALQTLYNYN
E. Shark β	GVLVQRYLSGETISTLR-TGFKAMV-FPAVTLCNVNPFRYSRSRGLLQPLDRLAELALQRIYMYN
Coelacanth β	GLLIQTYLSYGVSTSLS-MGFRAME-FPAVTVCNVNPMKYSEPPKLIQSMHFLIFLFLTKKISKR
Ropefish β	GILIQTYMSWGVSTSLS-VGFKTAP-FPAVTICNVNPFKYSKVKPLIEDLDEAARTALAKIHTYF
W. Lungfish β	GVLIQTYLSWGVSVSLS-VGFSSLA-FPAVTICNSNPFKYSRIKPLLTELDGFAASLLERIYIYS
A. Lungfish β	GLLIQTYLSWGVSVSLS-VGFRGMD-FPAVTVCNVNPFKYSKVKPLLKELDELVDILLEQFYSYS
Frog β	GVLILTYLSYGVSVSLS-IGFKTME-FPAVTLCNANPFKYSRVKPLLKELDELVATALDRIQFSS
Cow β	GLFIKTYLNWEVSVSLS-IGFKTMD-FPAVTICNASPFQYSKVQHLLKDLDELMEAVLGRILGPE
Human β	GIFIRTYLSWEVSVSLS-VGFKTMD-FPAVTICNASPFKYSKIKHLLKDLDELMEAVLERILAPE
Chicken β	GILINTYLSYNVTSSLS-IGFKTMK-FPAVTVCNANPFKYSEVRPLLKELDKLIEAALERILQPT
Turtle β	GILIDTYLSYSVSSSLS-IGFKTMK-FPAVTVCNASPYKYSKVRHLLKELDELTEAALERILQSK
S. Lamprey γ	IQLIQSF--YSIAVSVT-INYQKLP-FPAITVCSLNPYKYNQSQALLEKLDRNTAVALHNIGIAV
J. Lamprey γ	IQLIQSF--YSIAVSVT-INYQKMP-FPAITICSLNPYKYNQSQALLEKLDRNAAVALHNIGIAV
Ropefish γ	ALLIQTY--YSSSVSVT-VQFQTLT-FPAVTVCNLNPLRYSATKQLLTELDEQAERALQELYSPK
E. Shark γ	ALLASAY--YTVSVSIT-VHFQELP-FPAITICNINPYRYSATRWLVGELEKATLTVLDELYKYT
W. Lungfish γ	TLLVMSY--YSVTVSVM-VKYQTLS-FPAVTVCNINPYKPNTTISLLDELNRQARKILEKLYGFC
A. Lungfish γ	TLLLMSY--YTVSVSVT-VQFQTLP-FPAVTICNINPYRKNATSALLEELDKQTKLILKELYTSC
Coelacanth γ	ALLIISY--YSVTVAVS-VQFQELN-FPAITICNINPYRYSATGELLQELERETKNALKVLYDFP
Frog γ	ALLLMSY--YSVSASIT-VTFQKLV-YPAVTICNLNPYSYSKVKDRLAALEKETSQTLKNIYGFT
Cow γ	ALLISSF--YTVSVSIK-VHFQKLD-FPAVTICNINPYKYSAVRHLLADLEQETRAALKTLYGFS
Human γ	ALLVFSF--YTVSVSIK-VHFRKLD-FPAVTICNINPYKYSTVRHLLADLEQETREALKSLYGFP
Chicken γ	AELLLNY--YSASVSVT-VQFQKLP-FPAVTICNINPYKYSSMKDYLSELDKETKKALETFYGFS
Turtle γ	AELIMSY--YTASVSVT-VQFQKLP-FPAVTICNINPYKYSAMKEHLSELDKETKNALETLYGFS

```
Spotted Gar γ-like       -----------------------------------------------------------------
Asian Arowana γ-like     -----------------------------------------------------------------
Coelacanth ASIC1         -----------------------------YWLDLLHQDLSVNQDSLGVL----------------
Catfish ASIC1            -----------------------------YWIDIMHANHSVNRQSMAMLK---------------
E. Lamprey ASIC1         -----------------------------ELLALLDERMEIVEPRFADAQVIAQLRK--------
E. Shark ASIC1           -----------------------------ELLTLLNNRYEIPDPHLAERHILEALVE--------
J. Medaka ASIC1          -----------------------------ELLALLNGRYEIRDPHLVEENVLQVLRE--------
Black Rock Cod ASIC1     -----------------------------ELLALLNGRYEIRDPHMVEEHVLQILKE--------
Lancelet γ-like          AFQNAGFIPKPQP----------------QGQNNPNSNGSTPATGSTTVATAPTTTQNSTS----
Lancelet α-like          PPSPGPRRKRGVDTSQDNRPEYDV-----DWEDPYPPREESAEEHELLRERRETGSSAGSADSQG
E. Shark α               -----------------------------------------------------------------
Frog δ                   APESGQQVVDLQD-LLNNLTGQVN-GGFYLDESIVLLKLQENGSGPALPG---------------
Coelacanth α             TSQHGTSDDSMQSDIRRSNRIILS-----APDRVPLQVLDEPAAEHARTENQMAGT---------
S. Lamprey α             PPATKAAGTAAAP----------------GIRLDTGVVLERTGPDT-------------------
J. Lamprey α             PPATQSAGTAAAP----------------GIRLDTGVVLERTGPDT-------------------
W. Lungfish α            PANSNQSACNNTS-NKQDS----------NNKYIPLEFLTYNDTQSGYPFKGTTNGSSSFN----
A. Lungfish α            PLTSGNQSACNSS-STAGTRAFDE-------SYMKLEFLNDENTAYSGPVKGATNSTSPVN----
Ropefish α               FSKKVQQNSTSHT-SATEKSRNSN-PL--FNKKFVLEVLNRETSVNFNTPEKKGNHGV-------
Frog α                   --------STGVQ-GWIPNNQRVKRDR--AGLPYLLELLPPGSETHRVSRSVIEEELQVK-----
Salamander α             SSGMDALYHESRRER--------------RSATFPLFPLERLSPEQGAGGRVRRSSGAAVREEEL
Cow α                    -------SSKTLV-AHARSRRDLR-----EPLPHPLQRLPVPAPPHAARGVRRAGSSM-------
Human α                  -------SFTTLV-AGSRSRRDLR-----GTLPHPLQRLRVPPPPHGARRARSVASSL-------
Chicken α                MSLARSDGSAQFS-HRRTSRSLLH-----HVQRHPLRRQKRDNLVSLPENSPSVD----------
Turtle α                 MSLVQSNWAAQSS-RKRSPRSLSH-----HVHRHPLRRHKRDEPASLKGNSPPVD----------
Cow δ                    FSDSMDALGAEVP----GPEPAFH-----LDRRIRLQRLRPLDGQNR------------------
Human δ                  LSKGRAALSATVP----RHEPPFH-----LDREIRLQRLSHSGSRVR------------------
Coelacanth δ             ASEDITPNDSIGDGAGHGIKNISDNMNITLDQSIPLVLIRDKDSLDSSSAHSFPLD---------
Chicken δ                TSASLFHVNEKSI-HVRDLPSTGNHNGSSFKLSQKFSLLRTTEFNNRT-----------------
Turtle δ                 TSIFSSRYKKDIS-VKGGSANLSS-SSFQLNRHISLVMLKEPDTGSR------------------
S. Lamprey β             DSSTPSAYDTSYAVG--------------PWQEIPLVLIDRRDPNRTVVTEVMCSRAAVGIETHT
J. Lamprey β             DSSTPSPYNTSYA-KA-------------PWQDIPLVLIDRRDPNRTVVTEVVSKQSYLINGSID
E. Shark β               QNRTLPPPMDDLEDKWSRGGAGLG-----PGPLVPLVIIEQTEKGEEVLVRILGDAESDVP----
Coelacanth β             ERHSLTPVWKTNL-KKLLILPKTSQACVYLQKPQFLYLVSFFNKYHILKIVVLPL----------
Ropefish β               ISGKVPELPLMA-----NTSLVSV-----FLQNIPLVIMDESDVNNPAIISLFENGTDGF-----
W. Lungfish β            QSGTLPDPLLDTT-SRNATGQNL------LWYHLPLVIIDETDGDNPVIINILGPNNLTSTNGTT
A. Lungfish β            TNGTLPVVFPDMR-SS-YLTGDPP-----PWYQIPLVMIDETDADNPTVTNVLGTDAL------S
Frog β                   QNQGNTFTHNNQT-RQ-NVTLDPA-----LWNHIPLVVIDETDPRNPIIHNIFDNNAVYSKN--S
Cow β                    LSQVNDTRALNLS----------------IWHHTPLVFINEQNPHHPVVLDLFEDNFNGSASNSP
Human β                  LSHANATRNLNFS----------------IWNHTPLVLIDERNPHHPMVLDLFGDNHNGLTSSSA
Chicken β                HGDPISPLLLNNS-NA-TEGLDLD-----LWNQIPLVLIDEQDKDNPVIVEIFETNQSAAGNQ-T
Turtle β                 HGATISAPPLNSS-ETVSQQLDLK-----LWNQIPLVLIDESDPDHPVIIDLFETDQSGSGTRPN
S. Lamprey γ             TNLSAAKRDD-------------------EPLPIPLVWLDTTVTNQTVVTDVISGKFHVVPGKVE
J. Lamprey γ             TNLSAAKRDD-------------------EPLPMPLVWLDTTVTNQTVVTDVISGKFHVVPGEVE
Ropefish γ               EPTEKRKRRGLVTDKHAEDGQVVSN----LLKDIPLFWLDKKTFSAAFAPN---NRKRTYPEKFL
E. Shark γ               DAEDDGGEVRPQH-E-AEPGEGSR-PS--LFQNIPLLQIQARGPEYSIVSNLLSRRQHRVNGTVS
W. Lungfish γ            TDCGSGNFSSRSL-SLDDIPVKTRSLN--LLQDMSLIKVETAKDGQLVASDIVTDLQYRISGTAI
A. Lungfish γ            TGCSNRKLRSVLL-NEAPEEDSGV-AK--LLQDMPLMKFEVIKEDHVIVSELSSNRQYRINNTFI
Coelacanth γ             LDDNESHVLRSTE-TSLNSESDKE-VL--FSRSLPLLKIEEMEQNYTIVSDVFSDVKQRVNAPLM
Frog γ                   EPLIRSKRDVGVNVENSTEDI--------FLKQIPLYRLESVKGSQLVVSDLKTKKRTRMSAKVI
Cow γ                    EITSRKRREAQSW-SSVRKGTDPK-----FLNLAPLMAFEKGDTGKA--RDFFTGRKRKVNARII
Human γ                  E--SRKRREAESW-NSVSEGKQPR-----FSHRIPLLIFDQDEKGKA--RDFFTGRKRKVGGSII
Chicken γ                EGKTKVRRAAGDWNGTESL----------FFRHVPLLRFE---NSFRAATDLRSGRKRKVEGSVF
Turtle γ                 EGKSKVRRDADDWNATESK----------FFEIIPLLKFE--DLSKKTVTEIPSGNKRKIETSVF
```

Spotted Gar γ-like	------------------------------------TDYANSSQNQKAY----------------
Asian Arowana γ-like	---------------------AQRWNSTGYPGA---TRDTN-RCA-NSF--------------S-
Coelacanth ASIC1	---------------------REDTKQKILQLS-----------NFTQY--------------TP
Catfish ASIC1	---------------------HSRHRERLMRLL-----------DFSDY--------------VP
E. Lamprey ASIC1	--HADFRVH--------------KP
E. Shark ASIC1	--KANFRNF--------------KP
J. Medaka ASIC1	--RADFESY--------------KP
Black Rock Cod ASIC1	--RANFDNY--------------KP
Lancelet γ-like	------------------------------------DEDDD-DYDYDGY--------------HG
Lancelet α-like	SSDSSDSSDSSD---------SSDSSDSSDSSDSSDSSDSSDSDYASYMYGGTWSSHYGQDGSS
E. Shark α	---SP
Frog δ	---------------------EKKFK-VGFKLC---NSSRD-DCYYKVF--------------WS
Coelacanth α	----DINNPALY---------KGEFRKIGFKLC---NASGL-SCFYQAY--------------SS
S. Lamprey α	---------------------------VGFKLC---NATGG-DCFYQSY--------------GS
J. Lamprey α	---------------------------VGFKLC---NATGG-DCFYQSY--------------GS
W. Lungfish α	---------------------NSEFYRVGFKVC---NDTDG-VCFYQIY--------------SS
A. Lungfish α	---------------------HTEFYRIGFKLC---NATGE-DCFYQTY--------------SS
Ropefish α	----AKNNPPLH---------NQNWQ-IGFKLC---NASGQ-DCYFQAY--------------SS
Frog α	---------------------RREWN-IGFKLC---NETGG-DCFYQTY--------------TS
Salamander α	PID------------------SISWN-IGFKLC---NDSGK-DCFYQKY--------------SS
Cow α	----RDNNPQVN---------RKDWK-IGFQLC---NQNKS-DCFYQTY--------------SS
Human α	----RDNNPQVD---------WKDWK-IGFQLC---NQNKS-DCFYQTY--------------SS
Chicken α	---------------------KNDWK-IGFVLC---SENNE-DCFHQTY--------------SS
Turtle α	---------------------KSDWK-IGFILC---NETNE-DCFHQTY--------------SS
Cow δ	---------------------------VGFKLC---NSTGG-DCVERAY--------------SS
Human δ	---------------------------VGFRLC---NSTGG-DCFYRGY--------------TS
Coelacanth δ	---------------------KKGFR-VGFRLC---NVTGK-DCFYQSY--------------SS
Chicken δ	---------------------GKRQSLVGFRLC---NATGG-NCFYKTY--------------SS
Turtle δ	---------------------KKHFK-VGFKLC---NATGG-NCFYKAY--------------SS
S. Lamprey β	VDNRVF---------------HIGFCKSALGVC---CDSAGDKCFYSEY--------------LS
J. Lamprey β	TVSVPPERAAADATSSLIPHLGKDVR-IGFRLC---DTAGD-KCFYSEY--------------LS
E. Shark β	----RDAKLGRG---------PRSYK-VALHLC---SKDGR-DCLYRNF--------------TT
Coelacanth β	---------------------ANEKKQKVSVLCKDERMGKKKKSSVYFT--------------NA
Ropefish β	----VQNNPHPP---------TSDLK-VAIKLC---NANKT-ECLYWNF--------------TS
W. Lungfish β	AGYPIP---------------PRRYK-VAFELC---NASGS-DCFYKNF--------------SS
A. Lungfish β	PTNNSTTNSSTE---------ARRYK-VAFHLC---NTNGT-DCFYKNF--------------SS
Frog β	SIRNSSEDQTSY---------SQRYK-VAMKLC---TNNNT-QCVYRNF--------------TS
Cow β	APGRPC---------------SAHRCKVAMRLC---SHNGT-TCTFRNF--------------SS
Human β	SEKIC----------------NAHGCKMAMRLC---SLNRT-QCTFRNF--------------TS
Chicken β	AAPPAPANVTSE---------EKKYK-LAVKLC---SHQGSNNCTYRNF--------------TS
Turtle β	NSSPALSNVTSE---------VKKHK-VAVKLC---HHKDIPQCMYWNF--------------TS
S. Lamprey γ	MRSYFSQNY------------QSSEPLIAIEVC---GEER--KCIYNAF--------------TS
J. Lamprey γ	MRSYFSQNY------------QSSEPLIAIEVC---GEEK--KCIYNAF--------------TS
Ropefish γ	SKSGSRLRF------------HEAQRQAGFQLC---NSTNVTDCVVYAF--------------DT
E. Shark γ	THSLGNADILNQ---------EHL---VGFKLCEGGDIDSS-DCTIYTF--------------TS
W. Lungfish γ	TRMYNNMDLTSL---------GGQGH-VGFKVC---NQGQD-NCVIYTF--------------NS
A. Lungfish γ	TRMYNNMDLATV---------GEQ---VGFKIC---DANKS-NCIIYTF--------------NS
Coelacanth γ	RKMFENVAIENQ---------GEL---VGFKLC---DTNGS-DCAIYTF--------------NS
Frog γ	HRDAESV--------------QDPGNMVGFKLC---DPKNSSDCTIFTF--------------SS
Cow γ	HKASDVMHI------------HNSKEVVGFQLC---SNDTS-DCAVYTF--------------SS
Human γ	HKASNVMHI-------------ESKQVVGFQLC---SNDTS-DCATYTF--------------SS
Chicken γ	HKDSSIVNS------------GDSNDIIGFQLC---DANNSSECALYTF--------------SS
Turtle γ	HQGSSMVNT------------GDPQDVVGFQLC---DPNNSSDCAVYTF--------------SS

```
Spotted Gar γ-like       ----IEDSNQL------GF--LLSKLN-----PDQQAEAGHQLEDMLISCHFHGEKCD-K-----
Asian Arowana γ-like     -----------------KFASEFNRLS-----DEEKLDMGHQLEDMLLFCNYHGQPCN-T-----
Coelacanth ASIC1         PAQ-----YQL------NTTDLINRL-------------GHQMEEMLLECRFQGETCT-S-----
Catfish ASIC1            PPR-----FHL------NTTEMIGRLS-------------HQLEDMLLLCRFRGESCT-Y-----
E. Lamprey ASIC1         RPFSMREFYE---------------------------RAGHELREMLLHCKFHGMNCT-P-----
E. Shark ASIC1           KPFNMREFYA---------------------------RAGHDMKDMLLHCIFKGEFCT-A-----
J. Medaka ASIC1          RSFNMREFYD---------------------------RTGHDIKDMLLSCSYRGTECS-A-----
Black Rock Cod ASIC1     RPFNMREFYD---------------------------RTGHDIKEMLLSCSYRGVECS-A-----
Lancelet γ-like          DFEMVN-----------SFMGLLVQLT-----ASMRQSLGHQGRDFIQECQFDGRTCS-H-----
Lancelet α-like          SSDYMSEDYHHEFELVQNFSSSILGLN-----RTSRRTMGHQYQDLVLECAYDGRSCS-R-----
E. Shark α               G----------------------------------------RSSEFIRSCKFNRVSCD-N-----
Frog δ                   GVNALHEWYKF------HYINIMSNIP------AVLNIANNFSKDFILTCHFNEVPCD-E-----
Coelacanth α             GMDAVREWYMF------HYVNIMVQVP-----MVTNPLQETHIRDFVFSCKFNHASCN-Q-----
S. Lamprey α             GVQAVTEWYTF------QYVNIMSQVP-----SYIKQSDDANIEDFIFSCMFSGMPCS-D-----
J. Lamprey α             GVQAVTEWYTF------QYVNIMSQVP-----SYIKQSDDANIGDFIFSCMFSGMPCS-D-----
W. Lungfish α            GVDALREWYKY------QYVNIMGNAP-----LSTYQEDNPQISNFVYACEFNKISCG-S-----
A. Lungfish α            GVDALREWYKF------QYINIMAQIP-----SQSNQEDDSQISNFVYACEFNKVSCG-V-----
Ropefish α               GVDAIREWYKY------HYINIMAQMSMNALADDDSNPDKADINNFVFACSFNGAVCS-K-----
Frog α                   GVDAIREWYRF------HYINILARVP------QEAAIDGEQLENFIFACRFNEESCT-K-----
Salamander α             GVDAIREWYRF------HYINILARVP----ATSGVPLNEDSFQNFIFACRFNEDSCS-E-----
Cow α                    GVDAVREWYRF------HYINILSRRR----QDTSPSLEEDVLGKFIFTCRFNQDSCN-E-----
Human α                  GVDAVREWYRF------HYINILSRLP-----ETLPSLEEDTLGNFIFACRFNQVSCN-Q-----
Chicken α                GVDAVREWYSF------HYINILAQMP------DAKDLDESDFENFIYACRFNEATCD-K-----
Turtle α                 GVDAVREWYSF------HYINILARMP------NTKALDESNFENFIYACRFNEVTCD-K-----
Cow δ                    GVVAAREWYRF------HYINILALLP--------AAHEDSHGSHFVFSCRYDDRDCH-A-----
Human δ                  GVAAVQDWYHF------HYVDILALLP-----AAWEDSHGSQDGHFVLSCSYDGLDCQ-A-----
Coelacanth δ             VMDAIQEWYKF------HFINIMSQVS-----PMTNVSDSSPIGNVIYSCQYNGKSCS-G-----
Chicken δ                GMDAILEWYRF------HYMNIMSQQP----VIINISDHEEKIEDMVYSCQYDGEPCR-P-----
Turtle δ                 GVDTIQEWYRF------HYMNIMSQLP----VIINISSHEEHIQNLVYSCQYDGEPCR-E-----
S. Lamprey β             GMTAVKQWFHF------NLLSLLGNLS-----TEEKSNLSSSGDELIRSCLFSSDTCS-A-----
J. Lamprey β             GMTAVKQWFHF------NLLSLLGELS-----NEEKSNLSSSGDELIRSCLFSNDACN-A-----
E. Shark β               GLQAVNEWYSL------HYMSLMANVS-----LEDRTAMGEHGQDFILSCNFGGHPCD-L-----
Coelacanth β             FLSRVTHNYVQ------NFFFT----------DSSLVYAPTEGRLSFLQVHYG-EMCA-YFSWTL
Ropefish β               GVDAVNEWYSL------HFMDIMSKFS-----INEKKQMAYSGKEFILTCLFGNQPCS-Y-----
W. Lungfish β            SLEAVKEWYTL------HYLNIMLRIP-----LAEKAAMGYSGKDLILTCFFGGIACD-Y-----
A. Lungfish β            SLEAVKEWYTL------QYIDIISKLP-----LSQKVEMGYSGKDFILTCLFGGEACN-Y-----
Frog β                   GVQALREWYLL------QLSSIFSNVP-----LSGRIDMGFKAEDLILTCLFGGQPCS-Y-----
Cow β                    ATQAVTEWYTL------QATNIFAQVP-----NQELVAMGYPAERLILACLFGAEPCN-Y-----
Human β                  ATQALTEWYIL------QATNIFAQVP-----QQELVEMSYPGEQMILACLFGAEPCN-Y-----
Chicken β                AAQAVTEWYIL------QSTSILSKVP-----LQERIRMGYQAEDMILACLYGAEPCN-Y-----
Turtle β                 AAQAVTEWYIL------QSTSILSKVP-----LQERIRMGYQPEDMILACLYGAEPCS-Y-----
S. Lamprey γ             AIDAVIQWYRL------HFINIMAIVP-----EKDKDKLGYSADEFIIDCLFSGTVCDPS-----
J. Lamprey γ             AIDAVMQWYRL------HFINIMAIVP-----EKDKDKLGYSADEFIIGCLFSGTVCDPS-----
Ropefish γ               GISAVQEWYWL------HFNNIIAQQS-----LETLVEMGYSAEEFISTCTFNEAMCS-L-----
E. Shark γ               GMAAVQEWYQL------HYYNLLAQVP-----PEDKRAMGYSADDLFLTCLYDGLPCD-S-----
W. Lungfish γ            GITALQEWYRL------NFIDIMAQVP-----NEKKAEMGYSADELIVSCMYDGQACD-S-----
A. Lungfish γ            GVTAILEWYRL------NYLNIMAQIP-----NEKKLEMGYSADDLIVTCMYDGQSCD-S-----
Coelacanth γ             GITAIQEWYRL------HYINIMAQVS-----WEKKQEMGYSADDLIVTCFYNGMPCN-S-----
Frog γ                   GVNAIQEWYRL------HYTNILAKIS-----MEDKIAMGYKADELIVTCFFDGLSCD-A-----
Cow γ                    GVNAIQEWYKL------HYMNIMAQVS-----QEKKINMSYSADELLVTCFFDGVSCD-A-----
Human γ                  GINAIQEWYKL------HYMNIMAQVP-----LEKKINMSYSAEELLVTCFFDGVSCD-A-----
Chicken γ                GVNAIQEWYKL------HYMNIMAQIP-----LETKEELSYSADDLLLTCFFDGLSCD-K-----
Turtle γ                 GVNAIQEWYKL------HYMNIMAQIP-----LETKVNMSYSAEDLLLTCFFDGISCD-T-----
```

Spotted Gar γ-like	---SFFNAFFNHKFGNCYTFNSLTKMENRGRLMR-RDVLNATKAGFSYGLTMELSIEQDEYIEQF
Asian Arowana γ-like	---SFFSGFINYKFGNCYTFNSHKQTDIRGRPIK-SESLNTTKAGFMYGLHLELFIQQNEYVRDI
Coelacanth ASIC1	---RNFTPIF-TRYGKCYTFNS-------G--KDGNPLLTTLKGGMGNGLEIMLDIQQDDYLPVW
Catfish ASIC1	---KNFTTIY-TRYGKCYTFNS-------G--LDGNPLLTTLKGGTGNGLEIMLDIQQDEYLPVW
E. Lamprey ASIC1	---QDFQTVY-TRYGKCYTFNS-------G--KDGRPLLTSMKGGMGNGLEMMLDIQQDEYLPVW
E. Shark ASIC1	---QDFKIVF-TRYGKCYTFNS-------GQIKD-QPILTTLEGGTGNGLELMLDIQQDEYLPVW
J. Medaka ASIC1	---ENFKVIF-TRYGKCYTFNS-------G--KDGQPLMVTMKGGTGNGLELMLDIQQDEYLPVW
Black Rock Cod ASIC1	---DNFKVIF-TRYGKCYTFNS-------G--QDGRPLMVTMKGGMGNGLELMLDIQQDEYLPVW
Lancelet γ-like	---RNFTTFEDSTYGNCFTFNK-------D--KDGEVLHTATSAGPLHGLSLILYIEQDEYIPAI
Lancelet α-like	---TDFGRILDEKYGNCYTFNS-------D--K--VLQRKVRDPGPAHGLQLTLYIEQDEYVPAV
E. Shark α	---NNYTTFSHPRYGTCYTLNT-------G-----PVSWRVLGPGSANGLTLTLQVGEG--LRFL
Frog δ	---REYIHFHHPIYGNCFTINN-------H--GK-ENSWYSPRPGKQYGLSMVVKADLHDNMPLL
Coelacanth α	---GNYTYFNHPVYGNCYTFNG-------G--ST-GNLWSSTKPGRENGLSLLLRTEQNDYIPFL
S. Lamprey α	---SEYSRFHHPTYGNCYTFNS-------A--NS-SKLWQASKPGRDYGLSLILRTEQNDYIPFL
J. Lamprey α	---SEYSRFHHPTYGNCYTFNS-------A--NS-SKLWQASKPGRDYGLSLILRTEQNDYIPFL
W. Lungfish α	---GNYTQFNHPQYGSCYTFND-------G--DD-NNPWISFSPGVESGLSLVLRTEQNDFLPYL
A. Lungfish α	---ENYTRFRHPVYGNCYTYND-------G--QS-ATPWASFVPGVGNGLSLVLRTEQNDFLPFL
Ropefish α	---GNYTTFHHPMYGNCYTFNS-------W--ED-GHEWSVSTPGVESGLSLLLRTEQNDFIPLL
Frog α	---ANYSSFHHAIYGNCYTFNQ-------NQSDQ-SNLWSSSMPGIKNGLTLVLRTEQHDYIPLL
Salamander α	---ANYTHSHHPLYGNCYTFNE-------DHSRN-DSRWASSMPGINYGLSLVLRTEQNDYIPLL
Cow α	---ANYSHFHHPMYGNCYTFND-------K--NS-SNLWMSSMPGVNNGLSLTLRTEQNDFIPLL
Human α	---ANYSHFHHPMYGNCYTFND-------K--NN-SNLWMSSMPGINNGLSLMLRAEQNDFIPLL
Chicken α	---ANYTHFHHPLYGNCYTFND----------NS-SSLWTSSLPGINNGLSLVVRTEQNDFIPLL
Turtle α	---ANYTHFHHPIYGNCYTFND----------GN-SSLWTSSLPGINNGLSLLVRTEQNDYIPLL
Cow δ	---QHFQTSHHPTYGSCYTFNG---------------VWAAQRPGVTHRISLVLRAEQLDHLPLL
Human δ	---RQFRTFHHPTYGSCYTVDG---------------VWTAQRPGITHGVGLVLRVEQQPHLPLL
Coelacanth δ	---SEYEHFHHPVYGICYIFKS-------N--GS-DTFWETSKPGIAYGLSLIIGTKQEDFIPLL
Chicken δ	---SDYVHFHHPVFGSCYTFNS-------K--GT-DPFWTATKPGIPYGLSLILRAEQKDHIPLL
Turtle δ	---SDYIHFHHQVYGSCYTFNS-------E--GT-DLFWKASKPGISYGLSLILKAEQNDRLPLL
S. Lamprey β	---TNFTTFFHPMYGNCFIFNW-------G--EN-ETVMQVSNPGVEYGLKLVLSIDQDEYIPFL
J. Lamprey β	---TNFTTLYHPMYGNCFIFNW-------G--EN-ETVMQVSNPGVEYGLKLVLSIDQDEYIPFL
E. Shark β	---RNFTRLFHPTYGNCYIFNW-------G--SS-GSVLTVSNPGAEFGLKVVLDISQEDYNPFL
Coelacanth β	LTVINFTQMFHPTYGNCYIFNW-------G--QD-GNALISSNPGADFGLKLVLDINQEEYIPFL
Ropefish β	---KNFSQIFHHFYGNCYIFNW-------G--LH-DKAISSSNPGGEFGLNVVLDINQKEYIPFL
W. Lungfish β	---TNFTQIYHPSYGNCYIFNW-------G--LD-GNVAVSSNPGVGFGLQLVVDVNQEEYIPFL
A. Lungfish β	---DNFTQFYHSSYGNCYVFNW-------G--LD-GNVLIVSNPGVGFGLQLALDVNQEEYIPFL
Frog β	---RNFTHIYDADYGNCYIFNW-------G--QEGENTMSSANPGADFGLKLVLDIEQGEYLPFL
Cow β	---RNFTPIFHPDYGNCYIFNW-------G--MT-EKALPSANPGTEFGLKLILDMGQEDYVPFL
Human β	---RNFTSIFYPHYGNCYIFNW-------G--MT-EKALPSANPGTEFGLKLILDIGQEDYVPFL
Chicken β	---KNFTQIYHPDHGNCYIFNW-------G--MD-KEALNSSNPGAEFGLKLILDISQQDYIPYL
Turtle β	---RNFTQIYHPDHGNCYIFNW-------G--MD-EEALISSNPGAEFGLKLILDISQQDYIPYL
S. Lamprey γ	---TSFKKLQHPILGNCFTFND-------G--SD-GKSLDIASAGIDYGLHMVLNTRQDNSLPYL
J. Lamprey γ	---TSFKKLQHPILGNCFTFND-------G--RD-GKSLDIASAGIDYGLHMILNTRQDNALPYL
Ropefish γ	---RNFTQSYHPTLGNCYTFNS-------G--YD-GEIIQSSTAGIKNGLIVVLNLGLEDYNPFL
E. Shark γ	---RNFSLHQHPLHGNCFTFNG-------G--EN-GRVLITRTGGSQNGLKVTLHLDEDEYNPYL
W. Lungfish γ	---RNFTLFQHPLHGNCYTFNS-------G--ND-GNILQTLIGGNARGLKLILYTENDDYNPFL
A. Lungfish γ	---RNFTLFQHPLHGNCYTFNS-------G--DD-GNILQTLTGGSEYGLKLTLYLENDDYNPYL
Coelacanth γ	---RNFTLFQHPVYGNCYTFNS-------G--AD-GSILKTSTEASEFGLNVILYIDHKDYNPFL
Frog γ	---RNFTLFHHPLYGNCYTFNS-------A--ER-GNLLVSSMGGAEYGLKVVLYIDEDEYNPYL
Cow γ	---RNFTLFHHPMYGNCYTFNN-------R--QN-ETILSTSMGGSEFGLQVILYINEEEYNPFL
Human γ	---RNFTLFHHPMHGNCYTFNN-------R--EN-ETILSTSMGGSEYGLQVILYINEEEYNPFL
Chicken γ	---RHFTRFHHPLHGNCYTFNS-------G--EN-GTVLSTSTGGSEYGLQVVLYIDEADYNPFL
Turtle γ	---RNFTPFHHPLHGNCYTFNS-------G--EN-GKVLTTSTGGSEYGLQVVLYIEEADYNPFL

Spotted Gar γ-like	SQA------AGIRLIIHDQKDMPFPEDDGVNIPPGQESDIAIVKVHVHRLRAPYSSTCTTGDGIH
Asian Arowana γ-like	THS------AGIRMLIHDHLATPFPEDEGVNIPPGTETDIGITKVGIRRLKHPYGSNCTDGEGIT
Coelacanth ASIC1	KETDETSLEAGIKVQIHSQEEPPFIDQLGFGVAPGFQTFVSCQQQKLMYLPPPWGDCKATPINSE
Catfish ASIC1	GDTDETSYEAGIKVQIHSQDEPPFIDQLGFGVAPGFQTFVSCQQQLLQYLPPPWGDCQSTAMNSE
E. Lamprey ASIC1	GETDETSFEAGIRVQIHSQDEPPFIDQLGFGVAPGFQTFVSCQEQRLTYLPYPWGDCKDTPPESE
E. Shark ASIC1	GETDETSFEAGIKVQIHSQSEPPFIDQLGFGVPPGFQTFVACQEQRLRYLPPPWGDCKSTPMDSD
J. Medaka ASIC1	GETDETSFEAGIKVQIHTQEEPPFIDQLGFGVAPGFQTFVSCQEQRLTYLPPPWGDCKSTPMDSD
Black Rock Cod ASIC1	GETDETSFEAGIKVQIHTQDEPPFIDQLGFGVAPGFQTFVSCQEQRLTYLPPPWGDCKASAMDSD
Lancelet γ-like	AEK------AGARVVIHNPYVFPFPESEGFDAAPGFLTSAGLRLTSITRLGGVYGNCT-NGQG--
Lancelet α-like	TPA------AGVRVVIHQPGEWPFPAEEGFDVGPGYSTSIGLQVTTIRRLGGKYGNCT-DGRDKD
E. Shark α	TPG------FGVRLMVHDPKQTPILEDDGIDLLPGLETSVSLRLESVRRLGGGLSDCTKDGKGVE
Frog δ	SQA------AGARIMIHNPNQPPLVEHEGFDIQPGTETSISVKQEEVIRLGGKYSQCTSDGSDLS
Coelacanth α	STV------AGARVMIHRQNQPPFMEDEGFNIRPGVETSISMKKVSRQQLGGLYSDCTEDGSDIG
S. Lamprey α	STV------AGARIMVHDQESPPFMEEGGFDMRPGFETSLGIRMLEATRMPDPYGNCTEDGSNVP
J. Lamprey α	STV------AGARIMVHDQESPPFMEEGGFDMRPGIETSLGIRMLEATRMPDPYGNCTEDGSDVP
W. Lungfish α	SNV------AGARVMVHDQNQPVFMEDSGFDIRPGVETSIGIKKEIISRLGGVYGNCTADGSDIN
A. Lungfish α	STV------AGARVLVHDQNQPPFMEDSGLDIRPGVETSIGMKKEIISRLGGVYGNCT-DGSDID
Ropefish α	STV------AGARVMVHSQNHPVFMEDGGFDIKPGVETSIGLRQEVFQRLGGEYGDCL-DGTDLD
Frog α	SSV------AGARVLVHGHKEPAFMDDNGFNIPPGMETSIGMKKETINRLGGKYSDCSEDGSDVD
Salamander α	STT------AGARVIIQAPDEPVLLNEGGFNIQPGVETSISMTKETMDRLGGAYSDCTEDGSDVE
Cow α	STV------TGARVMVHERDEPAFMDDAGFNLRPGVETSISMSKEAVDRLGGDYGDCTKNGSEVP
Human α	STV------TGARVMVHGQDEPAFMDDGGFNLRPGVETSISMRKETLDRLGGDYGDCTKNGSDVP
Chicken α	STV------TGARVMVHDQNEPAFMDDGGFNVRPGIETSISMRKEMTERLGGSYSDCTEDGSDVP
Turtle α	STV------TGARVMVHEQNEPAFMDDGGFNVRPGMETSISMRKETTMRLGGSYSDCTEDGSDVL
Cow δ	STK------AGIKVMIHPQDHTPFLEHQGFSIRPGTETTIDIREDEVHRLGSPYGQCMDSTGSVD
Human δ	STL------AGIRVMVHGRNHTPFLGHHSFSVRPGTEATISIREDEVHRLGSPYGHCTAGGEGVE
Coelacanth δ	STV------AGTRVMIHKQDQPAFMEDEGLNIKPGTETSIGMKQDEVNRLAGNYGQCTFDGTDVK
Chicken δ	STV------AGVKVMIHNHNQTPFLEHEGFDIRPGIATTIGIQQDKVNRLGGNYGKCTTDGSDVK
Turtle δ	STV------AGVQVMIHNHNQTPFLEHEWFDIRPGIATNIGIRQDEVHRLGGNYGKCTVDGADVD
S. Lamprey β	TTI------AGAVIMVHDQNTYPFLSDLGVFVKTGVETSVGIEVGQLQRQGAPYSDCTMDGTDLP
J. Lamprey β	TTI------AGAVIMLHDQNTYPFLSNQGFFVKTGAETSVGIEVGQLQRQGAPYSDCTMDGTDLP
E. Shark β	SMA------AGAKFMLHQQNTFPFLRDLGMYAKAGTESSITIFADEIERLGGVYSRCRLNPSATE
Coelacanth β	TTS------AGARLMLHDQNTFPFLKDLGMYAMAGSQTSIGILVDEIQRIGAPYSQCTPYGSDVP
Ropefish β	STT------AGARLLVHEQRSFPFLKDLGIFVLPGTETSIGISVDKIERMEAPYSDCTQNGSDVP
W. Lungfish β	TTS------AGVRFLLHDQKTFPFVETMGIYALVGTVTSVEILVDEVMRMEQPYGTCTADGSDVP
A. Lungfish β	TTR------AGARFLLHTQNTFPFVETMGTYALVGTVTSVGILVDEVQRMGQPYGTCTTDGLDVP
Frog β	QTT------AAARLILHQQRSFPFVKDLGIYAMPGTETSISVLVDQLEHMEAPYSSCTVNGSDIP
Cow β	TST------AGARLMLHEQRSYPFIKEEGIYAMAGMETSIGVLVDKLQRKGEPYSQCTKNGSDVP
Human β	AST------AGVRLMLHEQRSYPFIRDEGIYAMSGTETSIGVLVDKLQRMGEPYSPCTVNGSEVP
Chicken β	SSA------AGARLMLHQQKSFPFLKDQGIYAMAGTETSIGVLVDELERMGYPYSDCTANGSDVP
Turtle β	TST------AGARLMLHEQKSFPFLKDQGIYAMSGTETSIGVLVDELERMGYPYSDCTMNGSDVP
S. Lamprey γ	AMG------AGAKIGIHLQNTTPFIEAVGIDIPPAMESSLGLRVNDVQKLGDPYSDCTMDGSDID
J. Lamprey γ	AMG------AGAKIGIHLQNTTPFIEAVGINIPPATESSLGLRVNDVQKLGEPYSDCTMDGSDID
Ropefish γ	SSS------EGAIIMIHNQNEHPFIEDLGIMIQTAKETSIGLQFMESHKLGEPYSSCTEDGTDVS
E. Shark γ	VTS------TGAKIVVHDQSEHPFVEDLGIAIPAGMETSIGLDLTESHKLGGPYSDCIE---DLP
W. Lungfish γ	FTS------MGAKVIVTDQNEYPLIDDVGLEVQTAMETLVGLQLTDSAKLSQPYSDCTVDGSDVM
A. Lungfish γ	FTS------MGAKIIVHDQTEYPLVDDVGLEIQTATETLIGLQVTTSAKLSKPYSDCTMDGSDVL
Coelacanth γ	VTS------TGAKVVIHDQNEHPFIEDMGLEVETATETSIGLQLTESHRLSSPYSNCTEDGSDVP
Frog γ	STA------AGAKILVHDQDEYPFIEYLGTELETATETSIGMQLTESAKLSDPYSDCTMDGRDVS
Cow γ	VSS------TGAKVIIHRQDEYPFVEDVGTEIETAMATSIGMHLTESFKLSDPYSQCTEDWSDVQ
Human γ	VSS------TGAKVIIHRQDEYPFVEDVGTEIETAMVTSIGMHLTESFKLSEPYSQCTEDGSDVP
Chicken γ	VTS------TGAKIIVHDQDEYPFIEDIGTEIETAAATSIGMHFTRSRKLSKPYSDCTETGADIP
Turtle γ	VTS------TGAKIIVHDQNEYPFIEDIGTEIETATATSIGMHFTRSHKLSKPYSDCTETGTDIP

```
Spotted Gar γ-like        NYYR--DVYK-VGYSR-------EACKKTCGQMYIIKNCGCGMWEFPVPKDVKVPFCNITNKNI-
Asian Arowana γ-like      NFYH--DLHG-FKYTR-------EACKRTCAQQSIMKDCGCSHWEFAVLPDLQYPKCNFSSPAT-
Coelacanth ASIC1          --------FF-STYSI-------TACRIDCETRYLVENCNCK--MVHMPGNAKV--CTPDQY---
Catfish ASIC1             --------FF-STYSI-------TGCRIDCETRYLLENCNCR--MVHMPGTSTV--CTPEQY---
E. Lamprey ASIC1          --------FF-DTYSI-------AACQIDCETRYLVENCNCR--MVHMPGDAPY--CTPEQY---
E. Shark ASIC1            --------FF-DTYSI-------TACRIDCETRYLVENCNCR--MVHMPGDAPY--CTPEQY---
J. Medaka ASIC1           --------FF-NSYSI-------TACRIDCETRYLVENCNCR--MVHMPGDAPY--CSPEQY---
Black Rock Cod ASIC1      --------FF-NTYSI-------TACRIDCETRYLVENCNCR--MVHMPGDAPY--CTPEQY---
Lancelet γ-like           RHLL----YP-QMYSQ-------QNCLATCHQEHMVEICGCADVTFLQPN---------------
Lancelet α-like           --NL----YR-SKYST-------KTCLHTCFQRLLVEKCGCGSRFIPLPKKVPA--CPVPI----
E. Shark α                IENL----YD-SSYSQ-------QTCVRSCFQALMTLRCNCSYFFYNKPKNSHY--CNSRSHPDW
Frog δ                    IKIL----YN-TSYTM-------QACLNSCFQYKMIEMCGCGYYFYPLPPGMEY--CNYNKYPGW
Coelacanth α              VENL----YN-SNYTQ-------QACVRSCFQVTLVQRCGCGHYFYPLPEGAQY--CNYKKHKTW
S. Lamprey α              VLNL----YS-SAYTV-------QVPECSCFQLALVEACGCGYYFYPLPPNASY--CSYNN-TAW
J. Lamprey α              VLNL----YS-SAYTV-------QACVRSCFQLALVEMCGCGHYFYPLPPNAAY--CSYNN-TAW
W. Lungfish α             VENL----YN-SDYTQ-------QACIRSCFQATIVERCGCGYYFYPLPAGATY--CTNTKHRGW
A. Lungfish α             VVNL----YN-SDYNQ-------QACVRSCFQATIVQQCGCGYYFYPLPSGAEY--CSYSRNKSW
Ropefish α                IENL----YE-SSYTQ-------QACIRSCFQLIMVKRCGCAYYFYPLPKGASY--CNYNRHIAW
Frog α                    VKNL----FQ-SEYTE-------QVCVRSCFQAAMVARCGCGYAFYPLSPGDQY--CDYNKHKSW
Salamander α              VKNL----FN-KKYTQ-------QACVRSCFQANMVQRCGCAYYFDPLPPGEEY--CDYHKQPNW
Cow α                     VENL----YN-TKYTQ-------QVCIHSCFQESMIKECGCAYIFYPRPDGVEF--CDYRKHNSW
Human α                   VENL----YP-SKYTQ-------QVCIHSCFQESMIKECGCAYIFYPRPQNVEY--CDYRKHSSW
Chicken α                 VQNL----YS-SRYTE-------QVCIRSCFQLNMVKRCSCAYYFYPLPDGAEY--CDYTKHVAW
Turtle α                  VQNL----YS-SRYTE-------QVCIRSCFQSSMVERCGCAYYFYPLPSGAEY--CDYTKHIAW
Cow δ                     VQLL----YN-TSYTR-------QACLVSCFQHLMVETCSCGYFFYPLPAGAEY--CSYMRHPAW
Human δ                   VELL----HN-TSYTR-------QACLVSCFQQLMVETCSCGYYLHPLPAGAEY--CSSARHPAW
Coelacanth δ              IK-L----YN-TPYSV-------QACVRSCFQYLLIQECGCGYYYYPLPPGAQY--CNYNKYPSW
Chicken δ                 VKLL----YN--SYTL-------QACLHSCFQHIMVQKCGCGYYYYPLPPGAEY--CNYNKQPAW
Turtle δ                  VKLL----YN-SSYTL-------QACLHSCFQDKMVERCGCGYYYYPLPPGAEY--CNYNKHPAW
S. Lamprey β              ITNL----YNGTAYSV-------QACLRSCFQTKMIEMCGCGYYLYPLPPGEKY--CQNQNFTGW
J. Lamprey β              ITNL----YNGTAYSV-------QACLRSCFQTKMIEMCGCGYYLYPLPPGEKY--CQNQNFTGW
E. Shark β                VTTL----YN-TSYSM-------QTCLRSCHQAHMVRLCGCAYHHYPLSEGAQY--CNNQDHPGW
Coelacanth β              VPNL-YSIYN-TSYSM-------QNCLYSCLQAKLVEKCGCGNYLHPLPDGAHS--CNNEDNPSW
Ropefish β                ITDLFYKMYE-TSYSV-------QSCLRSCFQMNLVKMCGCAYNLYPLPAEAPY--CNFGDHPEW
W. Lungfish β             VNNL-YSSYN-LSYSM-------QSCLWSCFQAQMVKRCGCAYYLYPLLDGANY--CDTQNNSDW
A. Lungfish β             IDNL-YSQYN-LSYTM-------QSCLWSCFQIQMVNSCGCAYYLYPLPEGATY--CNNQNNSDW
Frog β                    VQNL-YAEFN-SSYSI-------QSCLRSCYQEEMVKTCKCAHYQYPLPNGSEY--CTNMKHPDW
Cow β                     IQNL-YSNYN-TTYSI-------QACIRSCFQEHMIRECGCGHYLYPLPHKRKY--CNNQEFPDW
Human β                   VQNF-YSDYN-TTYSI-------QACLRSCFQDHMIRNCNCGHYLYPLPRGEKY--CNNRDFPDW
Chicken β                 VKNL-YSEYN-TSYSIQLPLSFFQACLRSCFQNHMTEICGCGHYMFPLPEGVTY--CNNEDNPGW
Turtle β                  VKNL-YNEYN-TSYSI-------QACLRSCFQAQMFENCGCGHYLFPLPEGVNY--CNNEDDPDW
S. Lamprey γ              VKSL----YD-SPYSV-------QTCQNSCFQWEMIKSCGCANYEQPLPEGSRF--CNYDNNPGW
J. Lamprey γ              VKSL----YD-SPYSV-------QTCQNSCFQLEMIKSCGCANYEQPLPEGSLF--CNYDNNPGW
Ropefish γ                VNNL----YN-KTYSL-------QVCLHSCFQKEMTLQCGCAHFHYPLPAEAQY--CNYNAFPDW
E. Shark γ                EDNL----YN-KSYSL-------QMCLHSCFQKEMVQTCGCGHYEKPLPPGAQY--CDYNRFPGW
W. Lungfish γ             EENL----YN-KSYSL-------QICLHSCFQKEMVNSCGCAYYEQPLPPGAEY--CSYEKFPGW
A. Lungfish γ             EQNL----YN-TSYSL-------QICLHSCFQTEMISNCGCAYYEQPLPSGAEY--CYYEKYPGW
Coelacanth γ              MQNL----YN-TTYSF-------QMCLYSCFQKEMVQSCGCAHYEYPLPVDTEY--CDYKKYPGW
Frog γ                    VENL----YN-KKYTL-------QICLNSCFQREMVRSCGCAHYDQPLPNGAKY--CNYEEYPSW
Cow γ                     ITNI----YN-ATYSL-------QICLHSCFQAKMVENCGCAQYSQPLPRGADY--CNYQQHPNW
Human γ                   IRNI----YN-AAYSL-------QICLHSCFQTKMVEKCGCAQYSQPLPPAANY--CNYQQHPNW
Chicken γ                 VENL----YN-KSYSL-------QICLHSCFQKAMVESCGCAQYAQPLPNGAEY--CNYKKNPNW
Turtle γ                  VANL----YN-KSYSL-------QICLHSCFQRAMVDTCGCAQYAQPLPPGAEY--CNYKKYPNW
```

Spotted Gar γ-like	--NKCVQLYEDKFAHDELEC--NCPLQCEEEIFELTLSSSQWPSAVYMNEFARKLRQSG------
Asian Arowana γ-like	--RRCLELYEYKFAQDILPC--HCPLQCKEELYSLTVSGSQWPATAFLDKFSSNLRGKG------
Coelacanth ASIC1	--KNCADPALDFLVEKD-NDYCVCQTPCNMTRYGKELSMVKIPSKASAKYLAKKFNKTE------
Catfish ASIC1	--KDCADPALDFLVEKD-NNYCVCPTPCNMTRYGKELSMVKIPSKASAKYLAKKFNKSE------
E. Lamprey ASIC1	--KECANPALMFLVEKD-DDYCACEMPCNIKRYAKELSMVKVPSQASAKYLAKKFNKTE------
E. Shark ASIC1	--KECADPALDFLVEKD-SVFCTCETPCNMTRYGKELSMVKIPSKASAKYLAKKYNKSE------
J. Medaka ASIC1	--KECADPALDFLVERD-NDYCVCETPCNLTRYGKELSFVKIPSKASAKYLAKKFNKTE------
Black Rock Cod ASIC1	--KDCADPALDFLVERD-NDYCVCETPCNMTRFGKEMSFVKIPSKASAKYLAKKFNKTE------
Lancelet γ-like	--VDCEEKVKQRLGNGNLTC--QCPISCMDRIYRKAIGLSEWPADSYVSTVLNKLKTKR------
Lancelet α-like	--NSCEQRWISEMRNGRIWC--DCPPSCVDNTLSMTFGFSEWPADSYEKSLRQKLSKLD------
E. Shark α	--GHCYYKLYEEFIAEKLNCFEKCPKLCDQSLYHITVGHSKWPSKQVESWMRPFLSNKK------
Frog δ	--GHCFYQLYEKMLDHTLICFTQCPKQCKQTQYHLAAGTAKWPSPVSKA--IQLLSLQE------
Coelacanth α	--GHCYYRLYKEFKANDLGCFTKCRKRCLESEYHQMTGYSKWPAKDSGKWIHHILAKQN------
S. Lamprey α	-AGHCYYKLYRQFISDELGCVDKCAQPCTTKRFAVTPGYAAWPDSSSEKWIFNLLSLQN------
J. Lamprey α	-AGHCYYKLYRRFISDELGCVDKCAQPCTTKRFAVTPGYATWPDSSSEKWIFNLLSLQN------
W. Lungfish α	--GYCYYKLYKAFAADELGCFKRCPKPCIVAEYVKTAGYSKWPSSSSETWIAKVLSQES------
A. Lungfish α	--GYCYYKLYKAFAADELGCFRRCRKPCQYTDYKMTAGYAQWPSSVSESWITSILSQEN------
Ropefish α	--GHCYYKLYNEFSLDNLGCSTKCRRPCQDTEYTMTAGYATWPTKASKNWIFNVLNKQN------
Frog α	--GHCYYKLIIEFTSNKLGCFTKCRKPCLVSEYQLTAGYSKWPNRVSQDWVLHTLSR--------
Salamander α	--GHCYYKLENEFVSDDLQCFTKCRKPCQLSEYHLSAGYSRWPSDVSKSWVFHMLSQQN------
Cow α	--GYCYYKLQDAFSSDRLGCFTKCRKPCSVTIYKLSASYSQWPSATSQDWVFQMLSRQN------
Human α	--GYCYYKLQVDFSSDHLGCFTKCRKPCSVTSYQLSAGYSRWPSVTSQEWVFQMLSRQN------
Chicken α	--GYCYYKLLAEFKADVLGCFHKCRKPCKMTEYQLSAGYSRWPSAVSEDWVFYMLSQQN------
Turtle α	--GHCYYKLQVEFKSNVLGCFSKCRKPCEVTEYQLSAGHSHWPSTVSEDWVSHMLSRQN------
Cow δ	--GHCFHHLYQKLKTHQLPCTTRCPRPCRESSYKLSAGTSRWPSSTSADWVLAVLGEPSRRNPWP
Human δ	--GHCFYRLYQDLETHRLPCTSRCPRPCRESAFKLSTGTSRWPSAKSAGWTLATLGEQG------
Coelacanth δ	ETGHCYYKLYKKFVAGDSGCFQKCPKPCQEFKYKLTTGISKWPSQNAENWIFHLLSHHN------
Chicken δ	--GHCFYQLYSRLRNHHLNCFDQCPKPCRESLYKVSAGTAKWPSRKSQDWIRQALRHQN------
Turtle δ	--GHCFYQLYNRLADHHLSCFAKCPKPCWESWYKLSAGTAKWPSTKSQDWVRQILSRQK------
S. Lamprey β	--RYCYYKLYEQFVEEDMDCYTICKQPCIESEYKMSISMSDWPSQSSEDWIFHILSKER------
J. Lamprey β	--RYCYYKLYDQFVEEDMDCYTICKQPCIESEYKMSISMSDWPSQSSEDWIFHILSKER------
E. Shark β	--AYGYYHLKEQIESENSECLTSCIPPCNDTLYRLTISMAEWPSQASEEWIYQILSYER------
Coelacanth β	--AYCYYSLGDSSEYKD-SCLQICEQPCNETQYRLTISMADWPSESSEDWIFHVLSYER------
Ropefish β	--VYCYHKWKKS-AESELLCLQTCKQSCNESQHLLTVSMADWPSESSEDWIFHVLSYER------
W. Lungfish β	--VYCYYHLQDSTDANE-ECIQICELPCIENQFRISTSMADWPSESSEDWIFHVLSHER------
A. Lungfish β	--AYCYYLLQDSKDHKN-ECLQTCIQTCNELQFRISTSMADWPSESSEDWIFHVLSYER------
Frog β	--VPCYYSLRDSVAIRE-NCISLCQQPCNDTHYKMVISMADWPSAGAEDWIFHVLSYEK------
Cow β	--AHCYSALRISLAQRE-TCIYACKESCNDTQYKMTISMAVWPSEASEDWIFHVLSQER------
Human β	--AHCYSDLQMSVAQRE-TCIGMCKESCNDTQYKMTISMADWPSEASEDWIFHVLSQER------
Chicken β	--AYCYSSLRSSIRHRQ-ICIDSCKETCNDTQYKMTISMADWPSEASEDWIFHILSYER------
Turtle β	--AYCYSSLRSSIRHRQ-FCIDSCKETCNDTQYKMTISMADWPSEASEDWIFHILSYER------
S. Lamprey γ	--EYCYYRLYDMYIKEELKCIQVCRQICSETEHEVTLSLADWPSKASKGWLLRALSKEQ------
J. Lamprey γ	--EYCYYRLYDMYIKEELKCIQVCRQICSETEHEVTLSLADWPSKASKGWLLRALSEER------
Ropefish γ	--MVCYSKLHAKFLQEELNCQKTCKGTCHTKEWILTESVAQWPSVNSEKWVLQTLRFGN------
E. Shark γ	--IYCYYRLRDRFHREQLVCQELCRQACHCKEWTLMTSVAQWPAQSAEDWVLRLLSWER------
W. Lungfish γ	--IYCYYQLQDKFVNERLPCQDVCKEPCNSKDFEITKSLAQWPSGASEAWVIRLLDWER------
A. Lungfish γ	--IYCYYQLQDKFVNERLACQDICKETCNSKDWDLTKSLARWPSVASKDWVLNLLNWER------
Coelacanth γ	--IYCYYKLRGKFAQEQLECQQVCKEACNSKEWALTKSLAHWPSLASEDWILRALNLQG------
Frog γ	--IYCYFKVYKQFVQEELGCQSACRESCSFKEWTLTRSLAKWPSLNSEEWMLRVLSWEL------
Cow γ	--MYCYYQLHQAFVREELGCQSVCKEACSFKEWTLTTSLAQWPSEVSEKWLLSILTWDQ------
Human γ	--MYCYYQLHRAFVQEELGCQSVCKEACSFKEWTLTTSLAQWPSVVSEKWLLPVLTWDQ------
Chicken γ	--MYCYYRLHEKFVKEQLGCQQICKDACSFKEWALTTSIAQWPSTVSEDWMLRVLSWDK------
Turtle γ	--MYCYYKLHETFVKEQLGCQQICKEACSFKEWTLTTSLAQWPSSVSEDWMLRVLSWDK------

```
Spotted Gar γ-like       ----------GKLAKVADK---VRD--------------------NLVKVIINYQQLNYELIEEI
Asian Arowana γ-like     ----------GQLKAIADNPQDIRD--------------------NMVKVVVYYQKLNYEHISEE
Coelacanth ASIC1         ------------DYIA--------E--------------------NILVLDIFFEALNYETIEQK
Catfish ASIC1            ------------QYIG--------E--------------------NILVLDIFFEALNYEKIEQK
E. Lamprey ASIC1         ------------AYIA--------E--------------------NVLVLDIFFEALNYETIEQK
E. Shark ASIC1           ------------DYIG--------E--------------------NILVLDIFFEALNYETIEQK
J. Medaka ASIC1          ------------QYIA--------D--------------------NILVLDIFFEALNYETIEQK
Black Rock Cod ASIC1     ------------QYIS--------D--------------------NLLVLDIYFEALNYETIEQK
Lancelet γ-like          ---------RGPGTGILDDRDEFKK--------------------NLLKLNIYYEALNYETITES
Lancelet α-like          ----------EESQEKLQTSHDARR--------------------NLLKLSVYFEQLNQQTISES
E. Shark α               ------------DFNV-------RK--------------------DFAKLNVYFEALSYREIEEI
Frog δ                   ------------RYNSTSE----RS--------------------DVSKINVYYEELSYRSVEET
Coelacanth α             ------------QYNFTTN---SRE--------------------DVSKLTVYFQELHHKTVGES
S. Lamprey α             ------------NYSVTTV----RN--------------------DVAKLNVYFRELNMKTISES
J. Lamprey α             ------------NYSVTTV----RN--------------------DVAKLNVYFRELNMKTISES
W. Lungfish α            ------------PYS-TSA----RK--------------------VIAKLNIYFYELSYKTTGES
A. Lungfish α            ------------QYNMTSG----RK--------------------NIAKLNVYFYELNYQTMGES
Ropefish α               ------------GYNITSD----RN--------------------DIAKVNIYFEDLNYRTFGES
Frog α                   ------------QYNLT-D----RN--------------------GIAKLNIYFEELNYKTILES
Salamander α             ------------QYNFTSD----RS--------------------GVAKLNIYFSEMTYKSTVES
Cow α                    ------------NYTIKNK----RD--------------------GVAKLNIFFKELNYKSNSES
Human α                  ------------NYTVNNK----RN--------------------GVAKVNIFFKELNYKTNSES
Chicken α                ------------KYNITSK----RN--------------------GVAKVNIFFEEWNYKTNGES
Turtle α                 ------------KYNITSK----RN--------------------GVAKVNIFFEEWKYKTNGES
Cow δ                    SSASIKSWPLPLPSSPSRA----RTEGPTSRSGAQPLSPEPCPSISLAKVNIFYQELNYRTVDET
Human δ                  -----------LPHQSHRQ----RS--------------------SLAKINIVYQELNYRSVEEA
Coelacanth δ             ------------GKNLTNN----RR--------------------DVSKLNIFFQKLSYESFDET
Chicken δ                ------------GYNSTSN----RK--------------------DIAKVTIYYKQLNYQSVNES
Turtle δ                 ------------GYNSTHN----RR--------------------DIAKVNIFYQQLNYQSVDES
S. Lamprey β             ------------KHNVSRI-FNRKQ--------------------DIIKLNLFFQEFNSMTISES
J. Lamprey β             ------------KHNVSRQ--------------------------DIIKVNLFFQEFNSMTTSES
E. Shark β               ----------DSSPKVTVN----RN--------------------SILKLNMYFKENNFRTISES
Coelacanth β             ----------DLAINRTMN----RN--------------------GALKLNLYFQEFNYRTISES
Ropefish β               ----------DFSSNVTIN----RD--------------------GVMKMNLYFKEINYRSITES
W. Lungfish β            ----------DNSSDITMN----SD--------------------GVLRLNFYFNEFNYRVISES
A. Lungfish β            ----------DDSTNITMK----RD--------------------GVLKLNLYFKEFNYRVITES
Frog β                   ----------DSSHNITVN----RN--------------------GIVRLNIYFQEFNYRSISES
Cow β                    ----------DQSSNITLS----RK--------------------GIVKLNIYFQEFNYRTIEES
Human β                  ----------DQSTNITLS----RK--------------------GIVKLNIYFQEFNYRTIEES
Chicken β                ----------DMSTNVTLD----RN--------------------GIIKLNIYFQEYNYRTISES
Turtle β                 ----------DLSTNVTLD----RN--------------------GIIKLNIYFQEYNYRTISES
S. Lamprey γ             ----------GLPANDTLK----PS--------------------DIAIVNIYFKDLTQKTISES
J. Lamprey γ             ----------GLPANDTLK----PS--------------------DIAIVNIYFKDLNQKTISES
Ropefish γ               ----------VLKKKQNIS----KE--------------------NFAKFSIFYKDLNLKTITES
E. Shark γ               ----------GRDGNKTLS----TS--------------------ELASLDIYYADLSVRNITEK
W. Lungfish γ            ------------GLNGTLS----KN--------------------DLVNLAIFYQDLNWRSLSES
A. Lungfish γ            ------------GLNNTLN----KN--------------------DLASIAIFYQDLNLRSLSES
Coelacanth γ             ----------SLKGNKGLS----KN--------------------DLVNLGIFYKDLNLRSISES
Frog γ                   ----------GEKLNKNLT----KN--------------------DLANLNIFYQDLNSRSISES
Cow γ                    ----------SQQIKKKLN----KT--------------------DLAKLLIFYKDLNQRSIMEN
Human γ                  ----------GRQVNKKLN----KT--------------------DLAKLLIFYKDLNQRSIMES
Chicken γ                ----------GQKINKKLN----KT--------------------DLANLMVFYKDLNERFISEN
Turtle γ                 ----------GQKINKMLN----KT--------------------DLANLVVFYKDLNERFISEN
```

Spotted Gar γ-like	PSFQVIDLVSSIGGLVGLWIGVSICTVAEFVE-LFLKVIIFIIKRVIR-----------------
Asian Arowana γ-like	PFITDIDLFSSVGGLVGLWVGVSLCTLAEFLE-FAVNVVLIVARGCLS-----------------
Coelacanth ASIC1	KAYEVAGLLGDIGGQMGLFIGASILTILEILD-YLYEVFKDQVLGYFK----RKKRPKRSH----
Catfish ASIC1	KAYEVAGLLGDIGGQMGLFIGASVLTILEIFD-YLYEVFKDKVLGYFLR-KGRPRRSASDN----
E. Lamprey ASIC1	RAYEVAGLLGDIGGQMGLFIGASILTILEIFD-YLYEIIKYRILYYFR----RNKKQRNIS----
E. Shark ASIC1	KAYEVAGLLGDIGGQMGLFIGASLLTILELFD-YVYEVLKHKMCGVLR--MGKQQKRNNND----
J. Medaka ASIC1	KAYELAGLLGDIGGQMGLFIGASILTVLELFD-YLYEILKYKLCRCMK---KKHKSRNNND----
Black Rock Cod ASIC1	KAYELAGLLGDIGGQMGLFIGASLLTILELFD-YLYEVIKYKLCRCVK---KKHKGRNNND----
Lancelet γ-like	PAYEVENLLGDLGGQLGLWVGMSCLSAMELLE-FLVDIAIIL------------WKKMSNR----
Lancelet α-like	PAYRVENLLGDLGGQLGLWVGVSVMTILEVLE-LIVDVTQILLS------KARGKKKTRV-----
E. Shark α	SAISVMELLSSLGGEVSVWFGGSVLSGFELLE-LLLDFLVLSIALL----KASVWRGH-------
Frog δ	PTMSVNVLLSSMGGLWSWWFGSSVLSVAEIAE-LVLDTAAMVTIVIYQ--WKKQRANRNDG----
Coelacanth α	PSINAATLLSNLGSQWSFWFGSSVLSVIEMVE-LLIDFFVLSTILLFR--HYCIQREENQD----
S. Lamprey α	AATNVIWLLSNIGSQWSLWFGSSVLSWLEVGE-LGIDCCIMVFVLAYR--RRRSRAERRRA----
J. Lamprey α	AATNVIWLLSNIGSQWSLWFGSSVLSWLEVGE-LGIDCCIMVFVLAYR--RRRSCAERRRARGTE
W. Lungfish α	PSFNVVTLLSNMGSQWSLWFGSSVLSVVEMGE-LVIDLIAVGIIVLRR----RQREKTKAT----
A. Lungfish α	PSFTVVTLLSNMGSQWSLWFGSSVLSVVEMGE-LVFDLIAVGVIVLRR----RRREKCQAS----
Ropefish α	PAFTAVMLLSNLGSQWSLWFGSSVMSVVELAE-LVFDLVAITLIFSAQ--KYFQWKNTESKVNTD
Frog α	PTINMAMLLSLLGSQWSLWFGSSVLSVVEMLE-LVIDFVIIGVMILLHRYYYKKANEGEET----
Salamander α	PAINMVVLLSLLGSQWSLWFGSSVLSVVEMAE-LLFDVAAITVILYLQ--RRRKRQMDSVE----
Cow α	PSVTMVTLLSNLGSQWSLWFGSSVLSVVEMAE-LIIDLLVITFLMLLRRFRSRYWSPGRGG----
Human α	PSVTMVTLLSNLGSQWSLWFGSSVLSVVEMAE-LVFDLLVIMFLMLLRRFRSRYWSPGRGG----
Chicken α	PAFTVVTLLSQLGNQWSLWFGSSVLSVMELAE-LILDFTVITFILAFR--WFRSKQ---------
Turtle α	PAFTVVTLLSQLGNQWSLWFGSSVLSVVELAE-LILDFIAITFILSFR--WLRSRQ---------
Cow δ	PVYSVPQLLSAMGSLWSLWFGSSVLSVVEVLE-LLLDAIALTLLLCCR--WLCGSRGQPRA----
Human δ	PVYSVPQLLSAMGSLCSLWFGASVLSLLELLE-LLLDASALTLVLGGR--RLRRAWFSWPR----
Coelacanth δ	PSISAVTILSQMGNLWSFWFGSSVLSVIELIE-LILDVIAMSFILTFK--WHKLKK---------
Chicken δ	PLLSDNLLLSSMGSQWSLWFGSSVLSVVEMLE-LLIDTLVLSLLFCYQ--RFRSKTLNVAR----
Turtle δ	PVYTVNLLLSNMGSQWSLWFGSSVLSVVEMFE-LLLDIMVLSLIFCYR--RFKAKKTLKMA----
S. Lamprey β	PAQTIVTLLSNLGGQFGFWMGGSVLCIIEFIE-IIIDCVWIGMIKASN--DVRERRKTSRK----
J. Lamprey β	PAQTIVTLLSNLGGQFGFWMGGSVLCIIEFIE-IIIDCVWIGIIKASH--DVRERRKTSRK----
E. Shark β	KAQTLVWLLSNLGGQFGFWMGGSVLCIVELLE-VMLDCVWIMAIRGAR--LYMGHRRRRSL----
Coelacanth β	AATDISWLVSNLEGQFGFWMGGSILCIIEFLE-IIIDCVWITIIKLVI--WYRDRKHKKAQ----
Ropefish β	ASTTVVWLLSNLGGQFGLWMGGSVICIVEFGE-IIIDCLWITIIKLIM--WNRNWKLKKAQ----
W. Lungfish β	AATNVVWLLSNLGGQFGFWMGGSVLCIIEFGE-IIIDCIWIAIIRFVI--WQKNRKKMQLP----
A. Lungfish β	VATNVVWLLSNLGGQFGFWMGGSVLCIIEFGE-VFIDCIWIAVIRFVK--WYKNRKERQVQ----
Frog β	EATNVVWLLSNLGGQFGFWMGGSVLCIIEFGE-IIIDCMWITILKFLA--WSRNRRQRRKR----
Cow β	AANNIVWLLSNLGGQFGFWMGGSVLCLIEFGE-IIIDFVWITIIKLVA--LAKSVRQKRAQ----
Human β	AANNIVWLLSNLGGQFGFWMGGSVLCLIEFGE-IIIDFVWITIIKLVA--LAKSLRQRRAQ----
Chicken β	AATTIVWLLSSLGGQFGFWMGGSVLCLIEFGE-IIIDSLWITVINIIS--WCKGLKQKRVR----
Turtle β	AATTIVWLLSSLGGQFGFWMGGSVLCLIEFGE-IIIDFLWITIINIIS--WCKGLKQKRAR----
S. Lamprey γ	PASSIVTLLSNLGGLLGLWLSCSMLCVVEVLEIFCVDFPWILLKKLLT--TCSSAFASLIR----
J. Lamprey γ	PASSIVTLLSNLGGLLGLWLSCSMLCVVEVLEIFCVDFPWILLKKLLA--TCRRTFASLVR----
Ropefish γ	PLNNIVTLLSNVGGQLGLWLSCSIVCVIEIIEVFFLDAPWILVRQIIR--SCQIRCRERQQ----
E. Shark γ	PANTMVTLLSNFGGQLGLWMSCSVICVIEIVEVLLVDALWVLVRSAGQ--RVRRWW---------
W. Lungfish γ	PANSIVTLLSNFGGQNGLWMSCSVVCFIEIWEVFLVDILTILVRNWFR--KAKLWWNKKKE----
A. Lungfish γ	PANSIATLLSNMGGQLGLWMSCSIVCFLEMWEVFLVDILTIIARYWLH--RGRQWWRKRKE----
Coelacanth γ	PANNIVTLLSNFGGQLGLWLSCSVVCVLEIIEVFFIDAFWIVLRQTTQ--KARDWWTKRK-----
Frog γ	PTYNIVTLLSNFGGQLGLWMSCSMICVLEIIEVFFIDSFWVVLRQRWR-----NWWENRKE----
Cow γ	PANSIEQLLSNIGGQLGLWMSCSVVCVIEIIEVFFIDSLSIIARHQWH--KAKGWW----A----
Human γ	PANSIEMLLSNFGGQLGLWMSCSVVCVIEIIEVFFIDFFSIIARRQWQ--KAKEWW----A----
Chicken γ	PANTLVILLSNFGGQLGLWMSCSVVCVIEIIEVFFIDSFSIVMRRQWQ--KAKKWWNHRKR----
Turtle γ	PANNIVILLSNFGGQLGLWMSCSVVCVIEIIEVFFIDSFFIVTRRRWQ--KAKKWWRDRKA----

Spotted Gar γ-like	-KNKEAPLNPYM-----------------------------IAHQTFKSSV--------------
Asian Arowana γ-like	-SRSQGPASSAL---
Coelacanth ASIC1	-SDNLSTCDTLR---------------------------------SHSDSLGFTPNMLPR-----
Catfish ASIC1	-LDYSENPTSPG-----------------------------------VTPNHTPRAHATH-----
E. Lamprey ASIC1	-DNSVPMTSSYA---APQGHTQAAIH---------------------------------------
E. Shark ASIC1	-KGVTLSLDDIK--RHNPC----------------------ESLRGHPTGMSYTANMLPH-----
J. Medaka ASIC1	-RGAVLSLDDVK--RHAPCE-------------------------NLRTPSTYPGNMLPH-----
Black Rock Cod ASIC1	-RGAVLSLDDVK--HHDPCD-------------------------NLRTPSTYPGNMLPH-----
Lancelet γ-like	-EKTTSRNNVVN--VAETG----------------------KIGTSNGHTIPIHMTEVM------
Lancelet α-like	--------------IDISGHM--
E. Shark α	---
Frog δ	-MNGSNAASNVC-----------------------------SVPYIDSKTFSTPQTGLSE-----
Coelacanth α	-PEITISTVSYP--HYANE----------------------NSEFNSEESMGNHFDVVAD-----
S. Lamprey α	-RGTGDSEAAPP---
J. Lamprey α	DSEAAPPPPPSF--REALGCANAAYDGANDGADDG------HDGDGGGVAIGGPTERPPPWRRGS
W. Lungfish α	-DDTEDNSPSET--YVHPRQENS------------------DNEHRERAPNRIEVVAEIS-----
A. Lungfish α	-SDGEGTSDSTA--GTHRGQ---------------------ENASRSGRDVACNRFVVVA-----
Ropefish α	GHQEPNTHNAVSNENHQEFDSLGVNFAFEAEDATHSIQSIPSDSPNVEMTTSFQFDVVAD-----
Frog α	-TVVPTPAPAFA--DLEQ-----------------------QVPHIPRGDLSQRQISVVA-----
Salamander α	-EDSQAPTPTLP---RFEGHGNPEFQ------------------SEEEPSHQFRVVADIT-----
Cow α	-KGTQEVASTPA--ASLPSS---------------------FCPHPAFFSSSPPDPAISP-----
Human α	-RGAQEVASTLA--SSPPSH---------------------FCPHPMSLSLSQPGPAPSP-----
Chicken α	-WHSSPAPPPNS-------HDNTAFQ---------------DEASGLDAPHRFTVEAVVT-----
Turtle α	-LLASAVPPPGA-------HDNTAFQ---------------PEPSGPSAPHRFTVEAVVT-----
Cow δ	-ATRVHPPSQRP------------------------------------ASGPVAADTTSN-----
Human δ	-ASPASGASSIK--PEAS-----------------------QMPPPAGGTSDDPEPSGPH-----
Coelacanth δ	---
Chicken δ	-TPSIPSVSLTL--ESYRVVQEAGN----------------GTAPAHGHTSGVPMAVANS-----
Turtle δ	-QPLAISSVTLTLENYRAVHEDLAADWNSIWTNQN------VGTVAKTKNGDLPFHSICN-----
S. Lamprey β	-PRYSDEPPTLS--SIVQGQGNSGFEMEERGPPG-------EAPEANGSAAQPPAAAAEQ-----
J. Lamprey β	-PRYSDEPPTLS--SIVQGQGNSGFEMEEREPPR-------EAPDANGSAAQPPTAAAEQ-----
E. Shark β	-ARHPPPVPTVA---------------------------------QCVEEQEHRAQSERP-----
Coelacanth β	-AQYSGPPPSVS--QLARAHTDTGFQ------HD-------STDINYGTEAYCNEAYIPP-----
Ropefish β	-SQYMGPPPSIS--QLAEGHMNTGFE------PD-------NVTSDSGQSLQTMSSCEHP-----
W. Lungfish β	-QLYNDPPPTVS--ELVEGISNQGFQ---------------PDIINSCTSQPQPPDLHIP-----
A. Lungfish β	-AQYADPPPTVS--ELVEGYTNQGFQ---------------PDIINSCSPQAQPPDLYLP-----
Frog β	-PQYSDPPPTVS--ELVEAHTNSGFQ--------------------HDDGDHVPVD---------
Cow β	-ARYEGPPPTVA--ELVEAHTNFGFQ------PD-------LATPGPDVE-AYPHEQNPP-----
Human β	-ASYAGPPPTVA--ELVEAHTNFGFQ------PD-------TAPRSPNTG-PYPSEQALP-----
Chicken β	-ARYPDTPPTVS--ELVEAHTNLGFQ-------------------HEEAGTETQGEALPP-----
Turtle β	-AQYPDTPPTVS--ELVEAHTNLGFQ------HE-------DTNITPCDE-VPPPGALPP-----
S. Lamprey γ	-GPDPAPSVHFP--VQLPVGGSPA-----------------EDPPTFHTAMQCPREPIPM-----
J. Lamprey γ	-GPDPAPSVHFP--VQLPVGGSPA-----------------EDPPTFHTAMQCPREPIPT-----
Ropefish γ	----RPTLPTVS--GGIPVD---------------------EDPPTFNSALRLPQPNLLE-----
E. Shark γ	---QRPEQEEVP--AHGTAHGITVYL------ED-------EDPPTFNAALRLP--RQCP-----
W. Lungfish γ	-GQ-EQVYN--Q--NRHTGHDNPVCV------ED-------EDPPTFHTAMQLPCVQTGE-----
A. Lungfish γ	-RQMQQPSP--P--DHDTGHHNPVCI------DD-------EDPPTFHTAMQLPCVQTGP-----
Coelacanth γ	-VRQAQPSLAAP--TDYAGQHNPVYVSD-------------EDPPTFSTAVHLPHSESCP-----
Frog γ	-NQAEDTPEIPV--PTMTGHDNPLCVDNPICLGE-------EDPPTFNSALQLPQSQDSH-----
Cow γ	-RRRAPACPEAP--RAPQGRDNPSLD------ID-------DDLPTFTSALSLPPAPGSQ-----
Human γ	-WKQAPPCPEAP--RSPQGQDNPALD------ID-------DDLPTFNSALHLPPALGTQ-----
Chicken γ	-DETGKPPEVGD--AEQQGHDNPAC-------SD-------EDLPTFNTALRLPLPQEGH-----
Turtle γ	-APAQVNAPAKE------GHDNPVCID--------------EDLPTFNTALHLPLPQENH-----

```
Spotted Gar γ-like       -----------------------------------------------------------------
Asian Arowana γ-like     ----------------------------------MAGREGPPS----------------------
Coelacanth ASIC1         -------------------------------------HPPLGNFEEFAC----------------
Catfish ASIC1            ------------------------------------SGVTRTASDSRRTCYLVTRL---------
E. Lamprey ASIC1         ------------------------------------PHGAHAKYEDFTC----------------
E. Shark ASIC1           -------------------------------------HPPRNTFEDFTC----------------
J. Medaka ASIC1          -------------------------------------HPGQGNFEDFTC----------------
Black Rock Cod ASIC1     -------------------------------------HPGQANFEDFTC----------------
Lancelet γ-like          -----------------------------------------------------------------
Lancelet α-like          -----------------------------------------------------------------
E. Shark α               -----------------------------------------------------------------
Frog δ                   -----------------------------------------KELESKTNDPKFNGEINTFS----
Coelacanth α             -------------------------------------VSAPPAYETLDLDLPPSAAQCITGCKCV
S. Lamprey α             ---------------------------------------PPPSF---------------------
J. Lamprey α             LTRNACSFCVVADISCPPAAAAKPVVGASREDAAAAPEALPPDYGSLRRVPEGYVAVSSDPGGPP
W. Lungfish α            ---------------------------------------PPPAYDSLELDTPVACSADCSCTRRM
A. Lungfish α            ------------------------------------EISPPPAYDTLQLDVPVACAPDCECTQHV
Ropefish α               -------------------------------------ISPPPAYDSLNLECSSLSQTIKGCNAEC
Frog α                   ------------------------------------DITPPPAYESLDLRSVGTLSSRSSSMRSN
Salamander α             ---------------------------------------PPPAYDSLELHAREECDDSCSCSHRS
Cow α                    -----------------------------------ALSAPPPAYATLGPHPAPSGLAEASTSAHA
Human α                  -----------------------------------ALTAPPPAYATLGPRPSPGGSAGASSSTCP
Chicken α                ---------------------------------------TLPSYNSLEPCGPSKDGETGLE----
Turtle α                 ---------------------------------------TLPSYNSLELCGQNRDAEIGVE----
Cow δ                    --------------------------------------APGPGCLHLPRCCRDFSRSLG------
Human δ                  -----------------------------------LPRVMLPGVLAGVSAEESWAGPQPLETLDT
Coelacanth δ             -----------------------------------------------------------------
Chicken δ                -------------------------------------SDPHPAQLSSKAIPEHCPDVVLNGFRYM
Turtle δ                 -------------------------------------KTPTPELNPDVVLNGFRHIKDHSIEIDL
S. Lamprey β             --------------------------------QPDVPGTPPPHYDTLRISKTELHDEINSDDDGE
J. Lamprey β             ---------------------------------QDVPGTPPPHYDTLRISKTELHDEINSDDDSE
E. Shark β               -----------------------------------VPSTPPPRYDSLHICSLTEPGPPEAGGKIV
Coelacanth β             --------------------------------REPTPGTPPPNYDSLRVQPVENTEQISDSEEN-
Ropefish β               --------------------------------VHPIPGTPPPHYDSLRLKSVRVNNE--------
W. Lungfish β            -------------------------------TTLEVPGTPPPHYDSLRIQPIDMEQQSDNEDF--
A. Lungfish β            -------------------------------TTLEIPGTPPPKYDSLRVHPIDTEHHSDSEDL--
Frog β                   -----------------------------------IPGTPPPNYDSLRVNTAEPVSSDEEN----
Cow β                    -----------------------------------IPGTPPPNYDSLRLQPLDVIESDSEGDAI-
Human β                  -----------------------------------IPGTPPPNYDSLRLQPLDVIESDSEGDAI-
Chicken β                -----------------------------------EPGTPPPNYDSLRVQPSHNPGTDSDIECEE
Turtle β                 -----------------------------------EPGTPPPNYDSLRVDPRAGIDSDSDAEAS-
S. Lamprey γ             ------------------------------------PNTPPPQYNTLRLRQIAGYVPDGGSDGE-
J. Lamprey γ             ------------------------------------PNTPPPQYDTLRLRQIAGYVPDEGSDGED
Ropefish γ               -----------------------------------VPKTPPPNYNTLRIHNVFSHMNDEDEHDTI
E. Shark γ               ------------------------------------PTAPPPNYEMLQQCHGFNGGPEEERF---
W. Lungfish γ            -----------------------------------VPSTPPPQYDALRIQNVFDEQFSDTEVN--
A. Lungfish γ            -----------------------------------VPSTPPPQYNALRIQSVFDEQVSDTEVN--
Coelacanth γ             -----------------------------------VPKTPPPTYDALRIQTAFAEQISDTEDNEY
Frog γ                   -----------------------------------VPRTPPPKYNTLRIQSAFQLETIDSDEDVE
Cow γ                    -----------------------------------VPGTPPPRYNTLRLERAFSSQLTDTQTTFP
Human γ                  -----------------------------------VPGTPPPKYNTLRLERAFSNQLTDTQMLDE
Chicken γ                -----------------------------------PPRTPPPNYSTLRLETAFTEQLPDTLEAGQ
Turtle γ                 -----------------------------------VPRTPPPNYSTLKLDAAFTDPLPDTLEGSC
```

Spotted Gar γ-like	---
Asian Arowana γ-like	---
Coelacanth ASIC1	---
Catfish ASIC1	---
E. Lamprey ASIC1	---
E. Shark ASIC1	---
J. Medaka ASIC1	---
Black Rock Cod ASIC1	---
Lancelet γ-like	---
Lancelet α-like	---
E. Shark α	---
Frog δ	---
Coelacanth α	HCASFISHEVEDLMSDLAGDP--------------------
S. Lamprey α	---
J. Lamprey α	RRGAGAPLLSPRVSALAASGKEVLRRRSASLNVVSFAIEEA
W. Lungfish α	SQTSIKSHTSNSSNTEESTSEGPTAL---------------
A. Lungfish α	SHASVHSQAPCSSQPEQEASEGPTVL---------------
Ropefish α	QCSRRLCEINEKD----------------------------
Frog α	RSYYEENGGRRN-----------------------------
Salamander α	SIRSNMSVHSRASSAATNA----------------------
Cow α	PGEP-------------------------------------
Human α	LGGP-------------------------------------
Chicken α	---
Turtle α	---
Cow δ	---
Human δ	---
Coelacanth δ	---
Chicken δ	KDSSLGGEINH------------------------------
Turtle δ	NS---------------------------------------
S. Lamprey β	FV---------------------------------------
J. Lamprey β	FVS--------------------------------------
E. Shark β	VEVGGEEAGEEWGEEGCRL----------------------
Coelacanth β	---
Ropefish β	---
W. Lungfish β	---
A. Lungfish β	---
Frog β	---
Cow β	---
Human β	---
Chicken β	QRPAANHHGDASVWAE-------------------------
Turtle β	---
S. Lamprey γ	---
J. Lamprey γ	---
Ropefish γ	TPIDDSIVHDNTMTINQRRRKENPFLII-------------
E. Shark γ	---
W. Lungfish γ	---
A. Lungfish γ	---
Coelacanth γ	---
Frog γ	RL---------------------------------------
Cow γ	H--
Human γ	L--
Chicken γ	H--
Turtle γ	H--
