# Supplementary figures and images for: Activation by cleavage of the epithelial Na+ channel α and γ subunits independently coevolved with the vertebrate terrestrial migration
Source: eLife. 2022 Jan 5;11:e75796. doi: 10.7554/eLife.75796 (PMC8791634; doi:10.7554/eLife.75796)

Figure 3. Blot 1

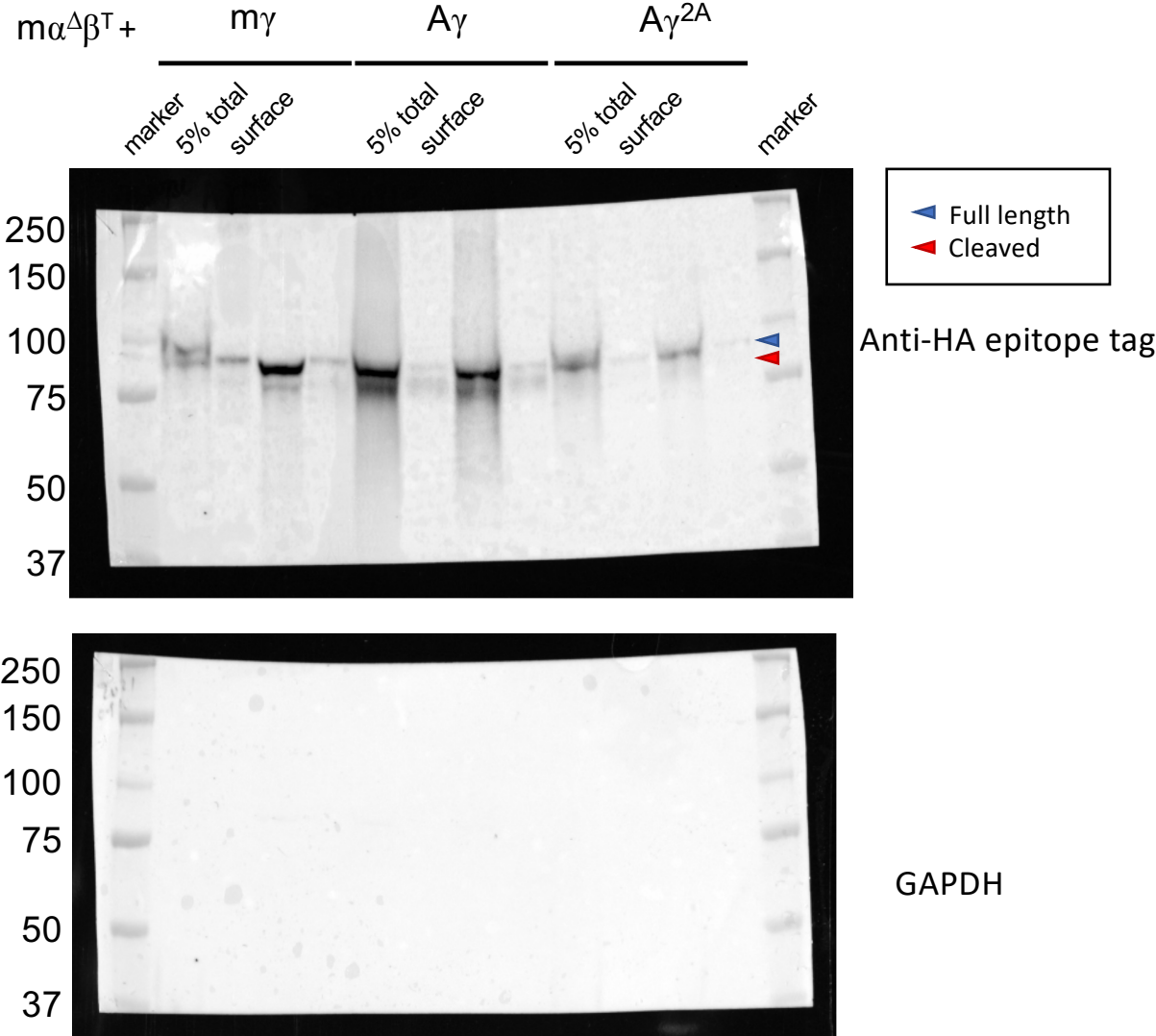

Figure 3. Blot 2

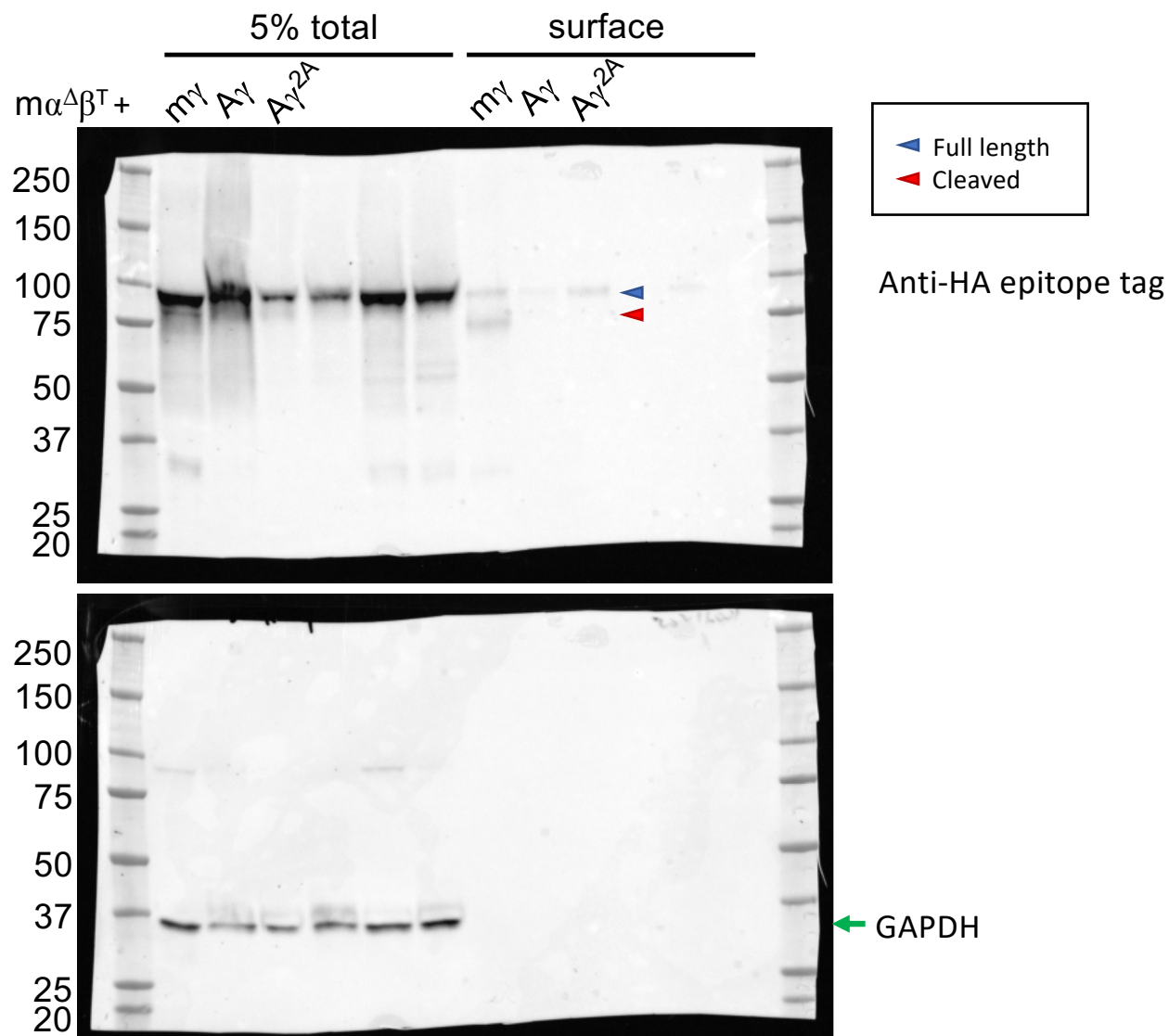

Figure 3. Blot 3

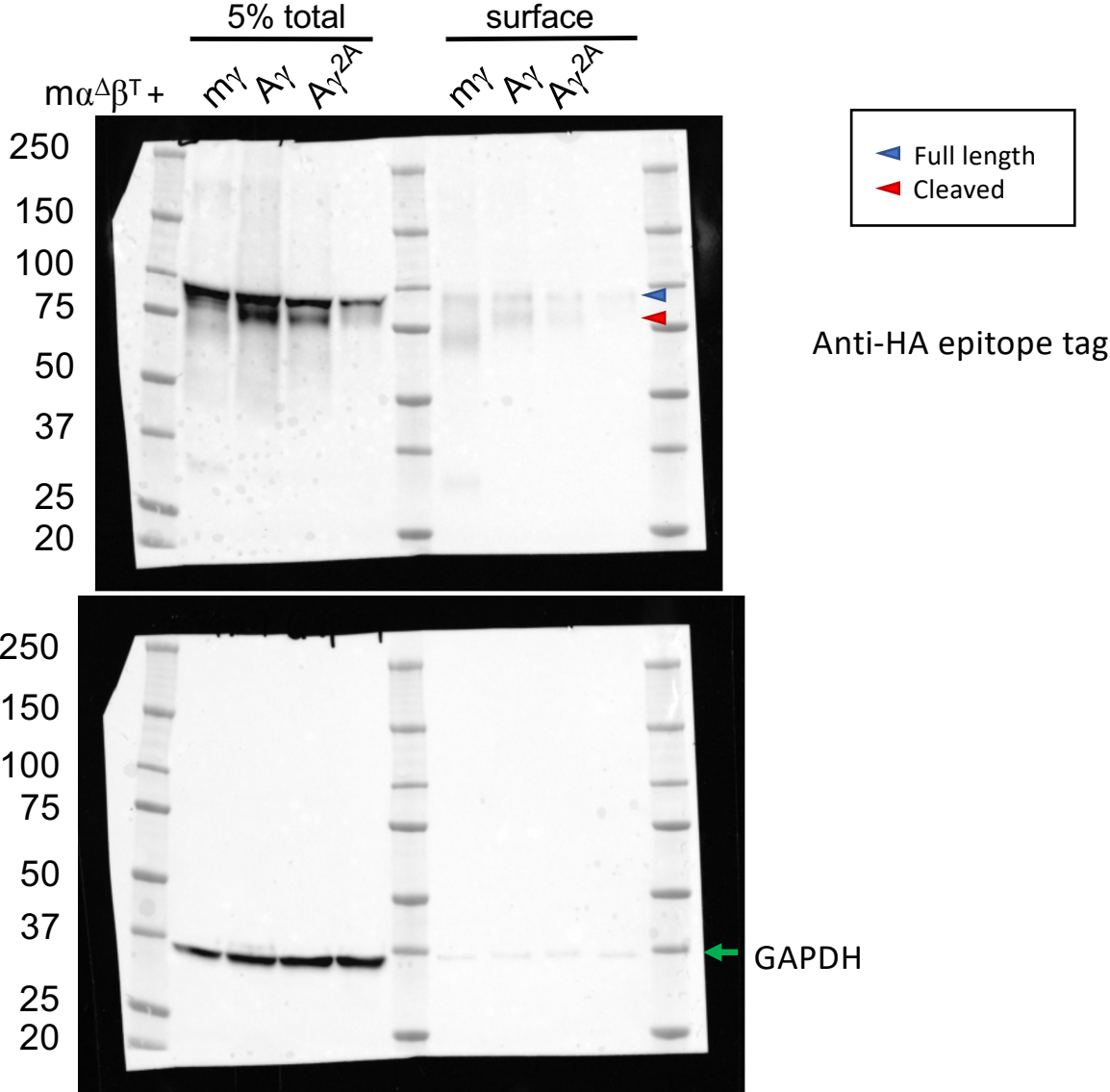

Figure 3. Blot 4

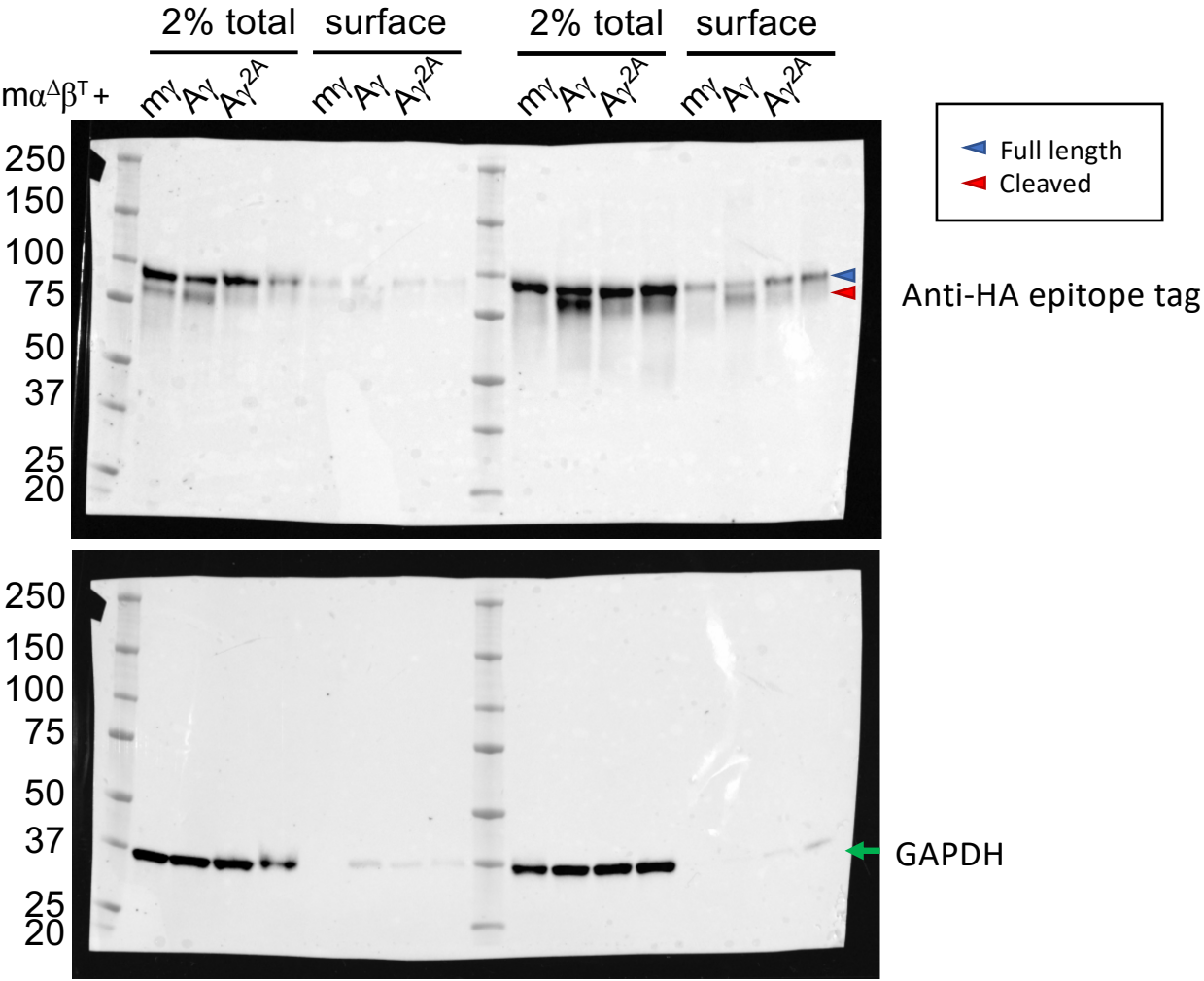

Figure 3. Blot 5

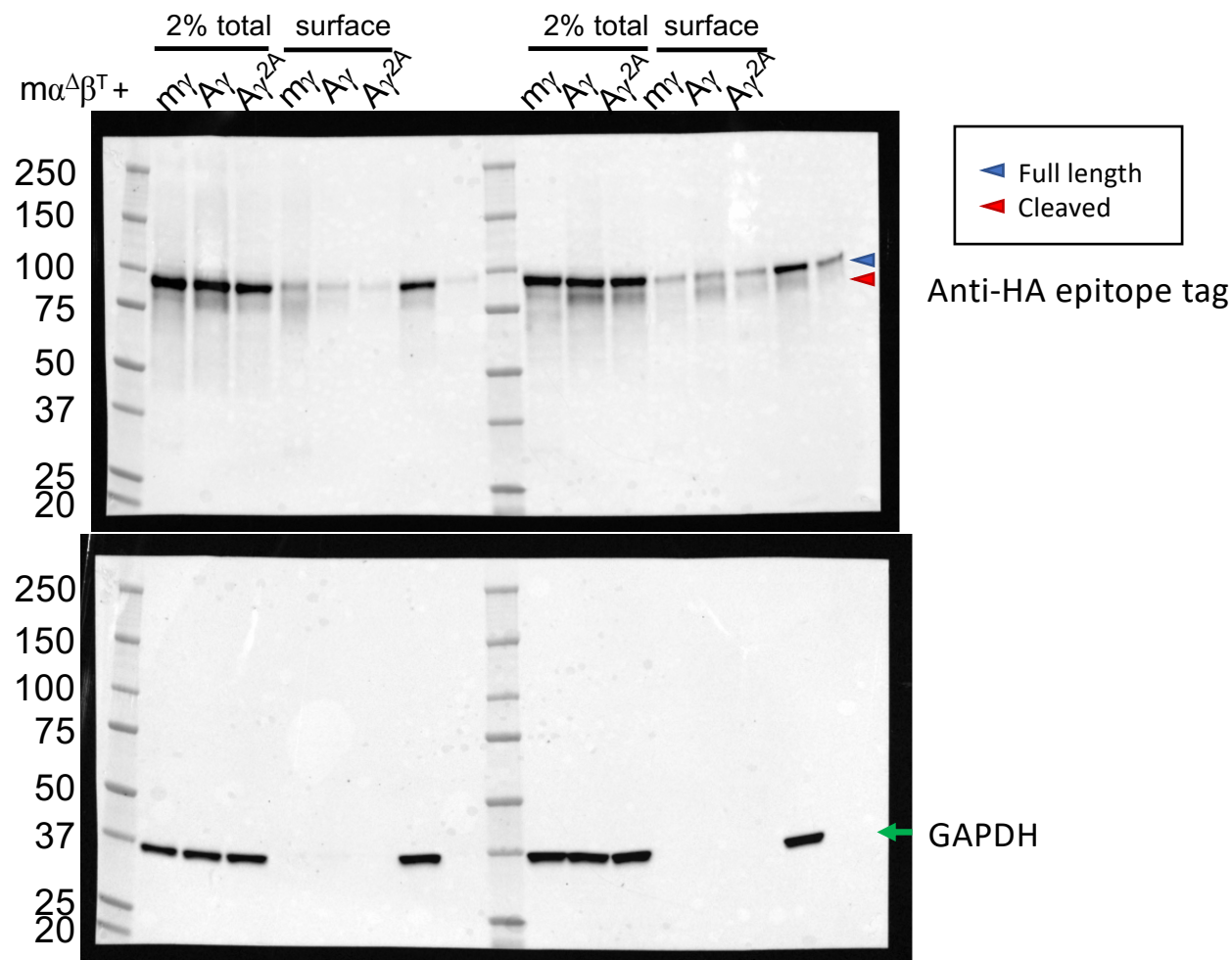

Supplement: Figure 3—source data 1. — Quantification for both blotting and two-electrode voltage clamp experiments are provided in a Microsoft Excel file. [file elife-75796-fig3-data1.zip › Figure 3-source data 1/Figure 3 uncropped labeled blots.pdf]

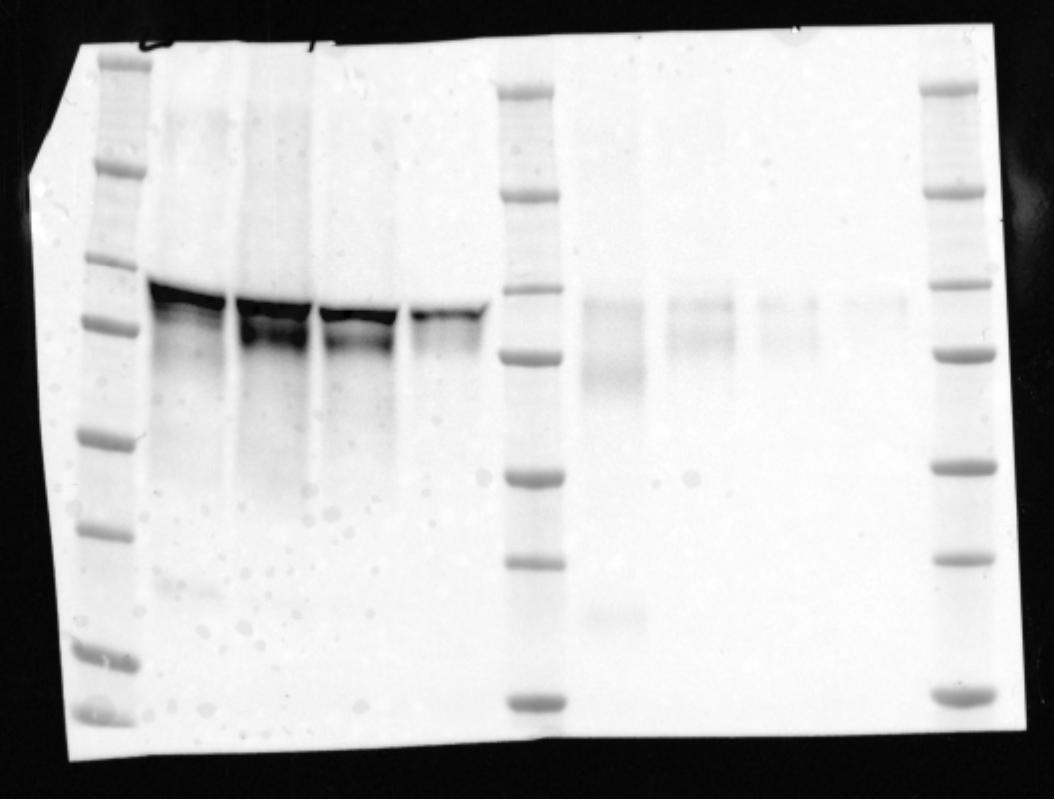

Supplement: Figure 3—source data 1. — Quantification for both blotting and two-electrode voltage clamp experiments are provided in a Microsoft Excel file. [file elife-75796-fig3-data1.zip › Figure 3-source data 1/Figure 3 original blots/Figure 3. Blot3 anti-HA.tif]

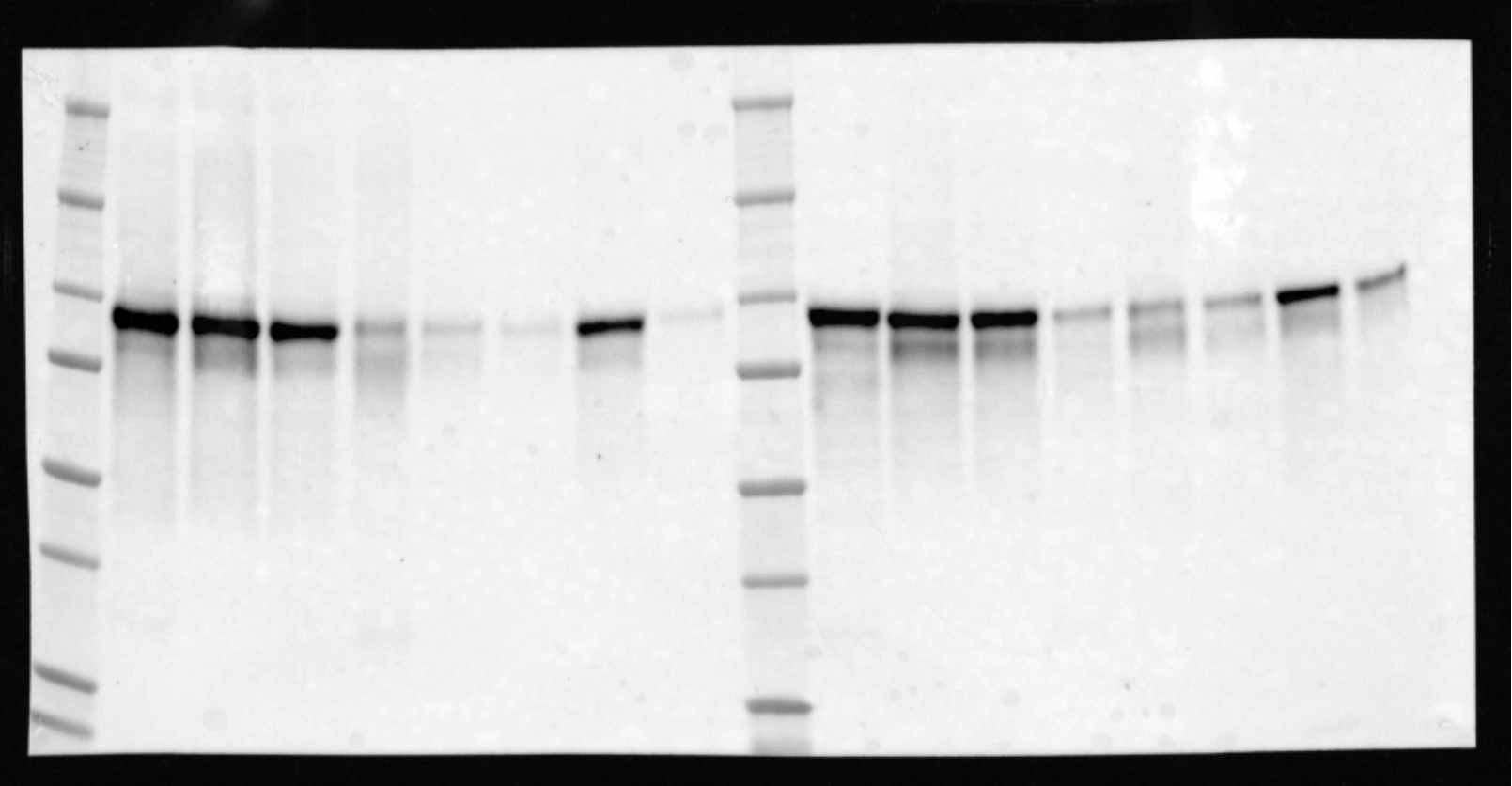

Supplement: Figure 3—source data 1. — Quantification for both blotting and two-electrode voltage clamp experiments are provided in a Microsoft Excel file. [file elife-75796-fig3-data1.zip › Figure 3-source data 1/Figure 3 original blots/Figure 3. Blot5 anti-HA.tif]

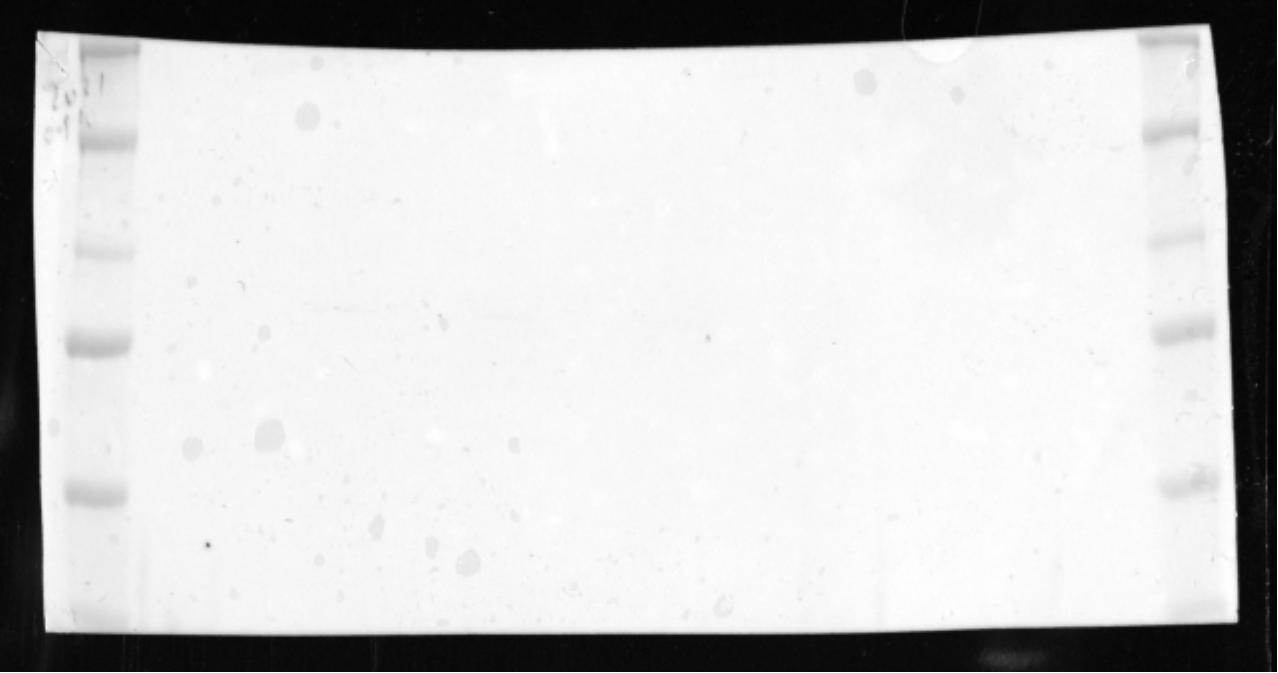

Supplement: Figure 3—source data 1. — Quantification for both blotting and two-electrode voltage clamp experiments are provided in a Microsoft Excel file. [file elife-75796-fig3-data1.zip › Figure 3-source data 1/Figure 3 original blots/Figure 3. Blot1 anti-GAPDH.tif]

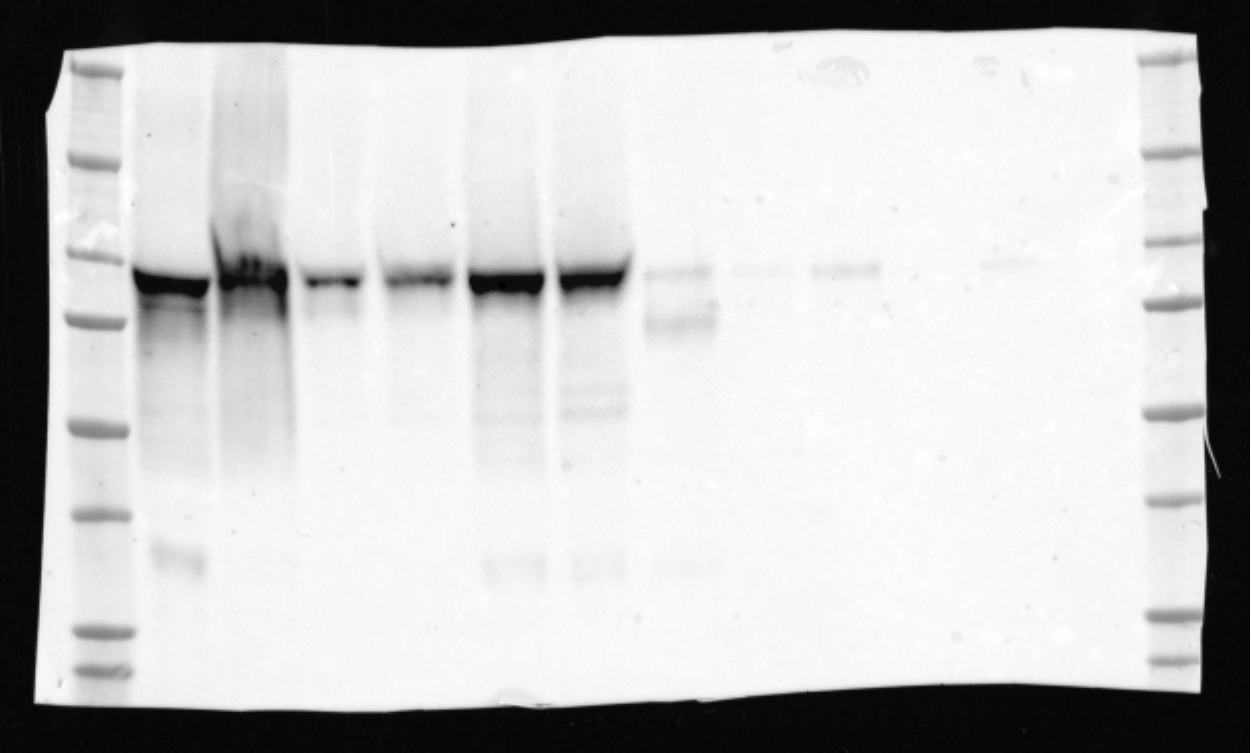

Supplement: Figure 3—source data 1. — Quantification for both blotting and two-electrode voltage clamp experiments are provided in a Microsoft Excel file. [file elife-75796-fig3-data1.zip › Figure 3-source data 1/Figure 3 original blots/Figure 3. Blot2 anti-HA.tif]

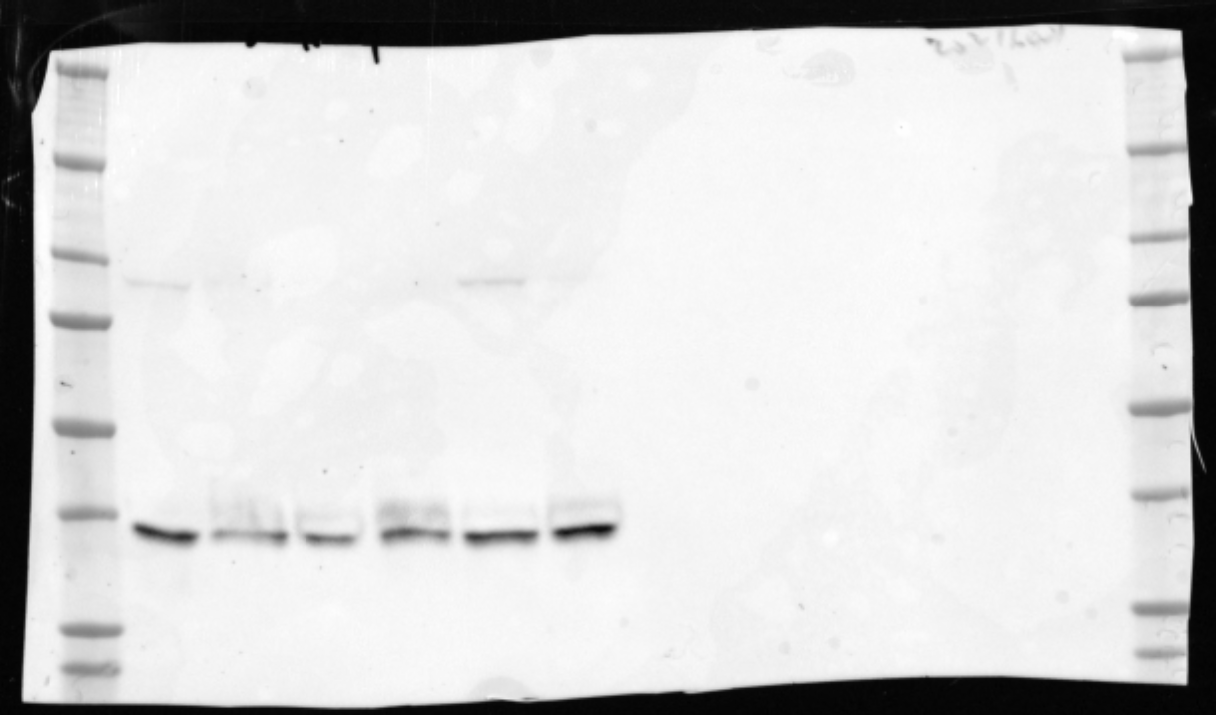

Supplement: Figure 3—source data 1. — Quantification for both blotting and two-electrode voltage clamp experiments are provided in a Microsoft Excel file. [file elife-75796-fig3-data1.zip › Figure 3-source data 1/Figure 3 original blots/Figure 3. Blot2 anti-GAPDH.tif]

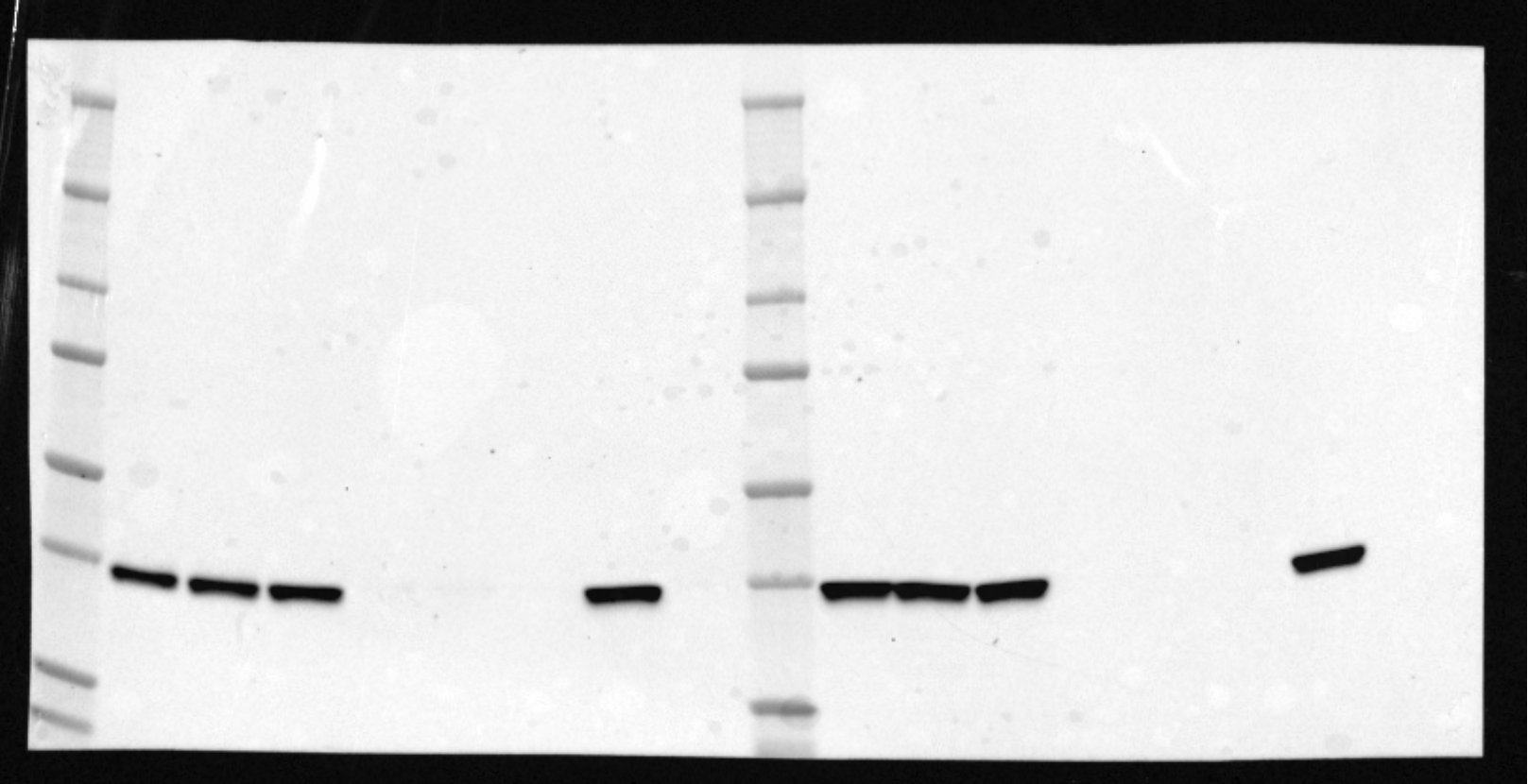

Supplement: Figure 3—source data 1. — Quantification for both blotting and two-electrode voltage clamp experiments are provided in a Microsoft Excel file. [file elife-75796-fig3-data1.zip › Figure 3-source data 1/Figure 3 original blots/Figure 3. Blot5 anti-GAPDH.tif]

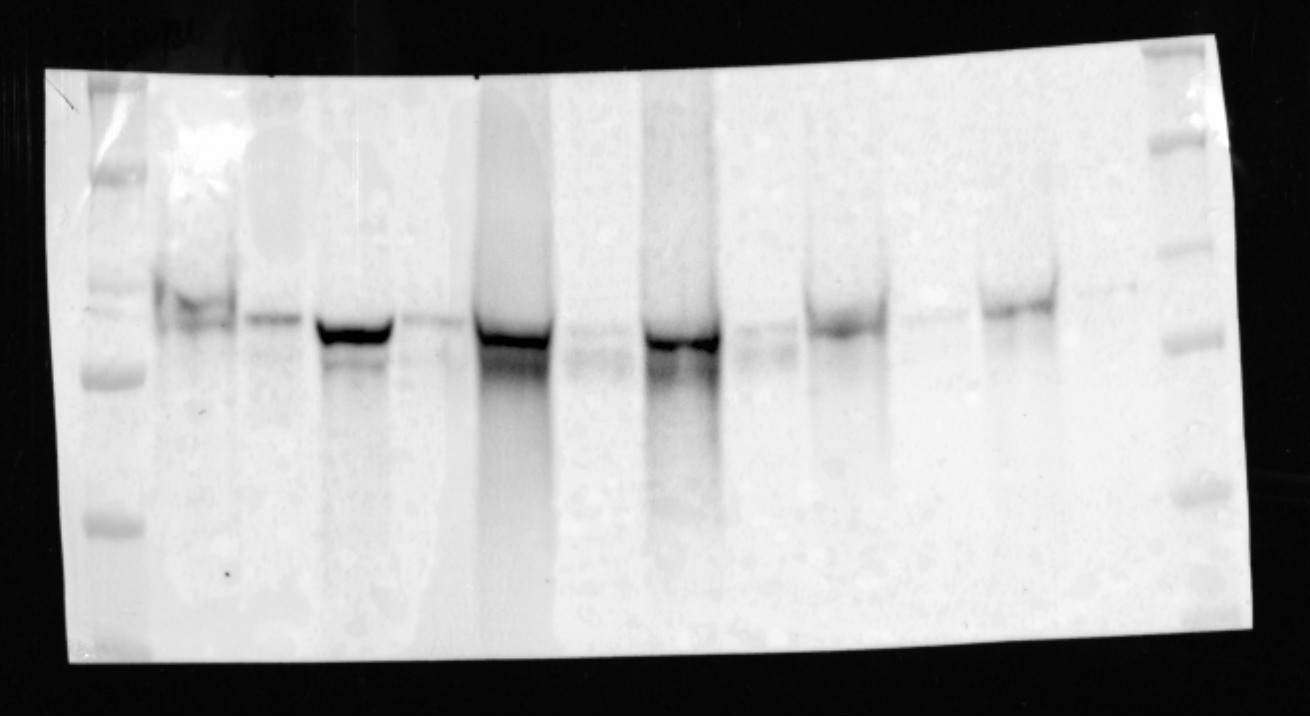

Supplement: Figure 3—source data 1. — Quantification for both blotting and two-electrode voltage clamp experiments are provided in a Microsoft Excel file. [file elife-75796-fig3-data1.zip › Figure 3-source data 1/Figure 3 original blots/Figure 3. Blot1 anti-HA.tif]

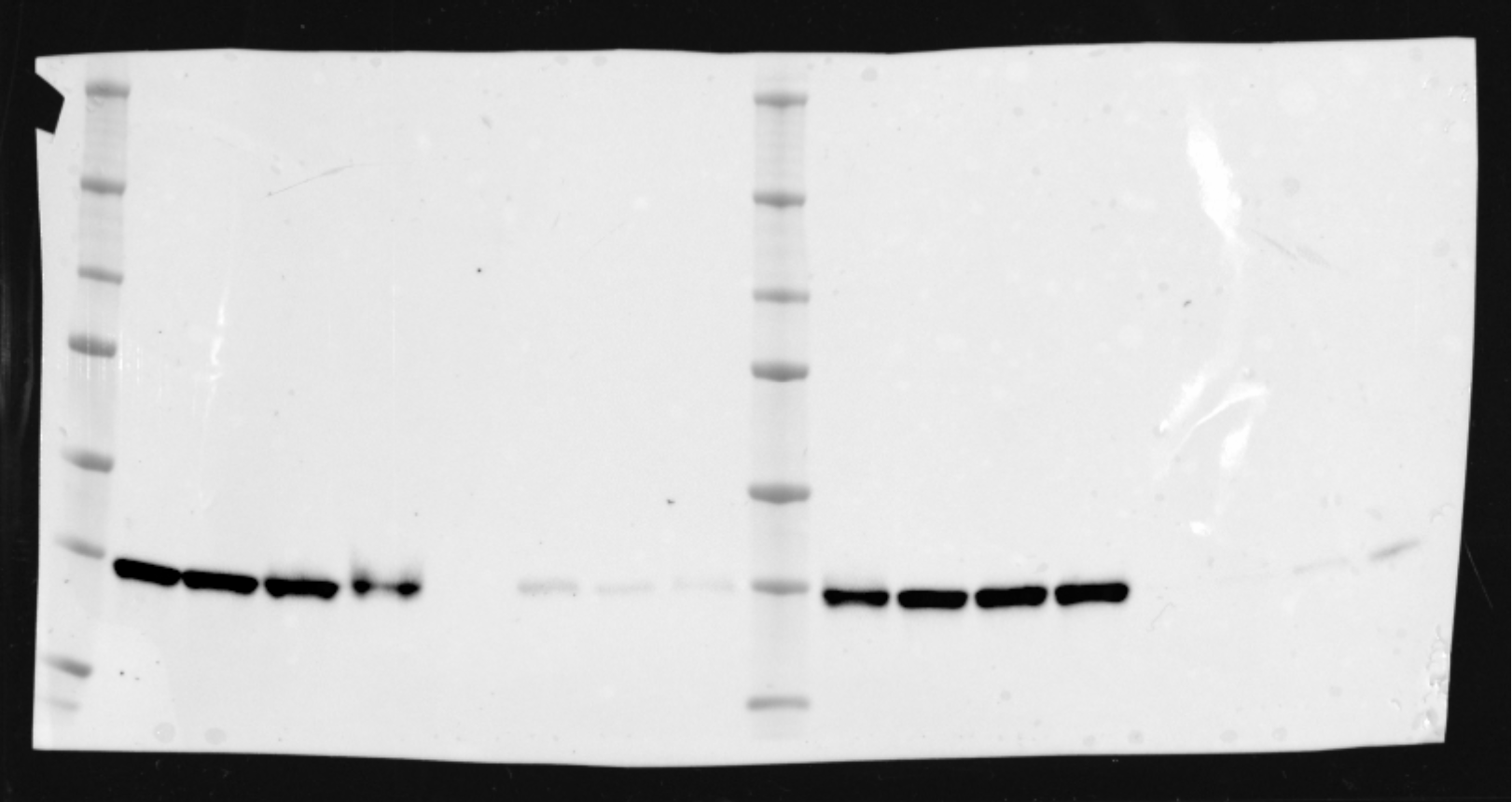

Supplement: Figure 3—source data 1. — Quantification for both blotting and two-electrode voltage clamp experiments are provided in a Microsoft Excel file. [file elife-75796-fig3-data1.zip › Figure 3-source data 1/Figure 3 original blots/Figure 3. Blot4 anti-GAPDH.tif]

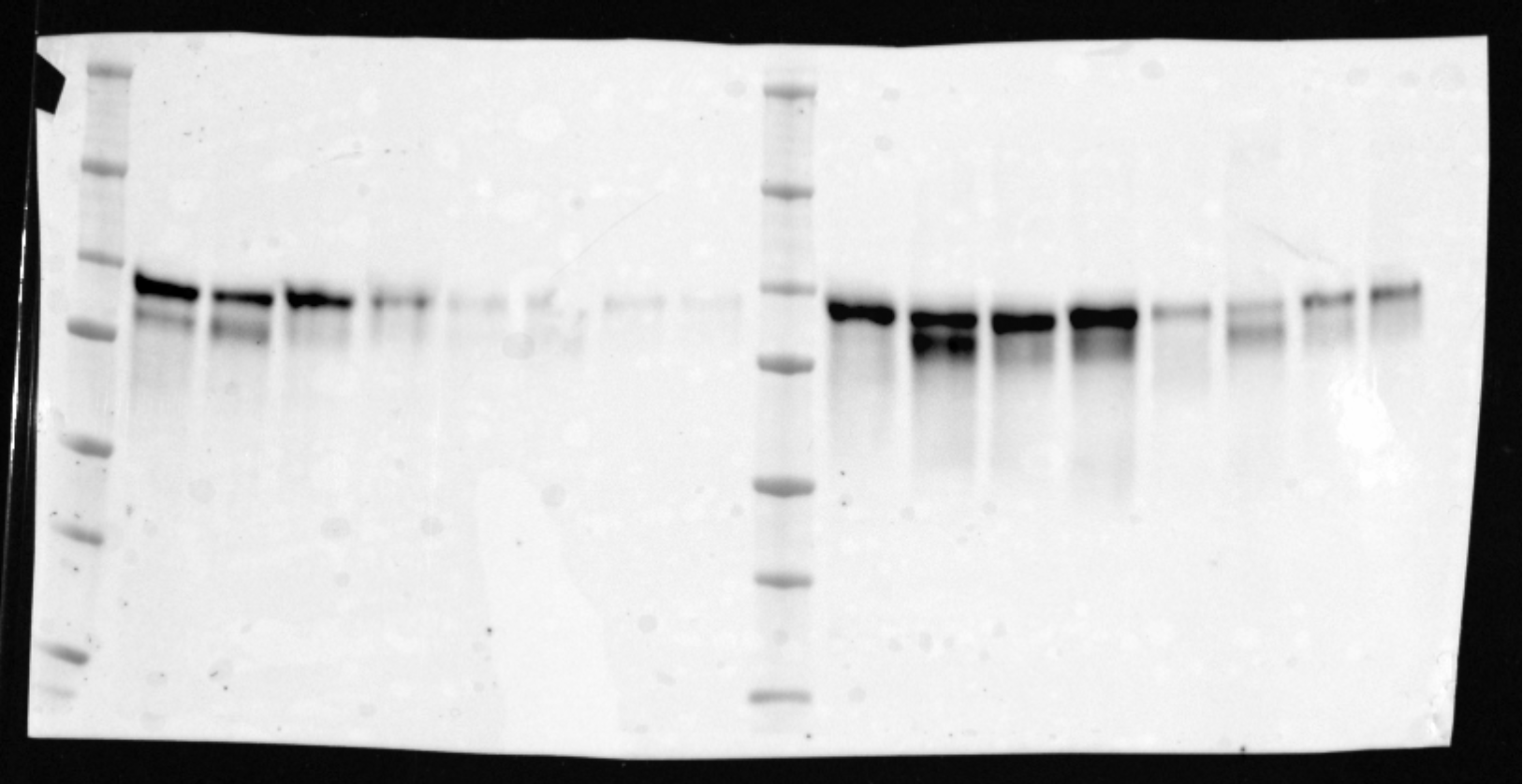

Supplement: Figure 3—source data 1. — Quantification for both blotting and two-electrode voltage clamp experiments are provided in a Microsoft Excel file. [file elife-75796-fig3-data1.zip › Figure 3-source data 1/Figure 3 original blots/Figure 3. Blot4 anti-HA.tif]

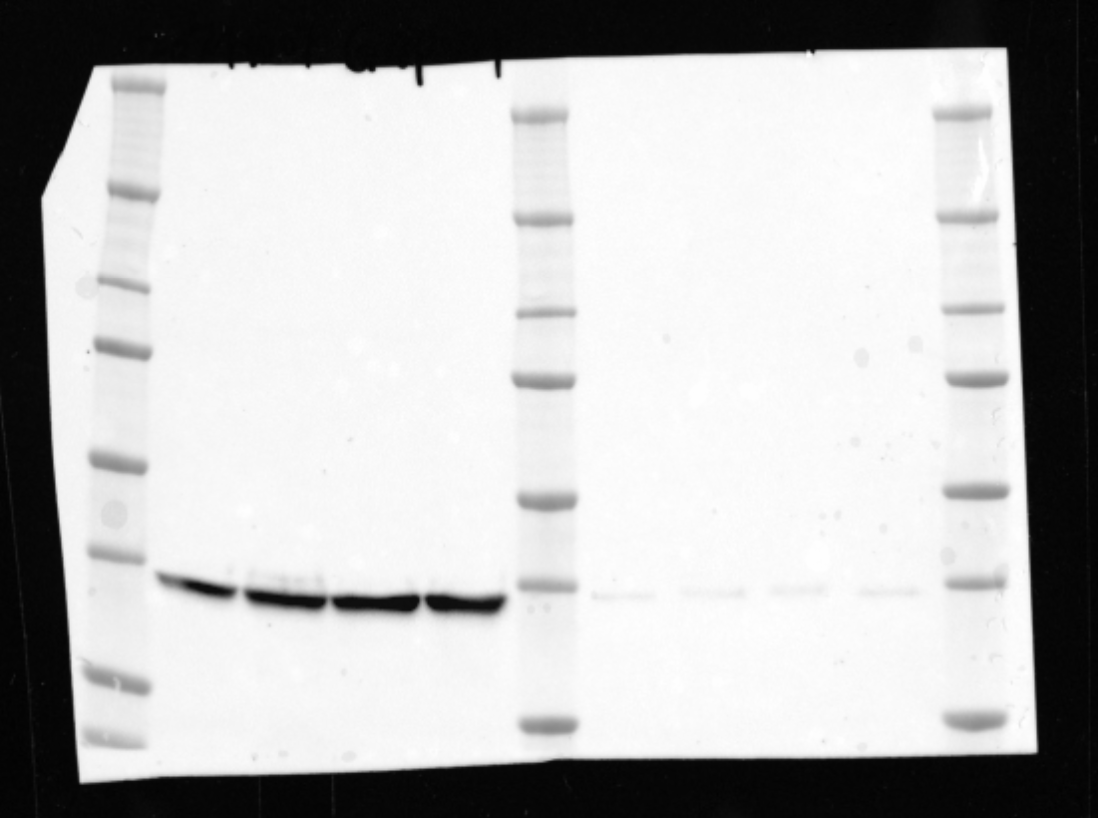

Supplement: Figure 3—source data 1. — Quantification for both blotting and two-electrode voltage clamp experiments are provided in a Microsoft Excel file. [file elife-75796-fig3-data1.zip › Figure 3-source data 1/Figure 3 original blots/Figure 3. Blot3 anti-GAPDH.tif]

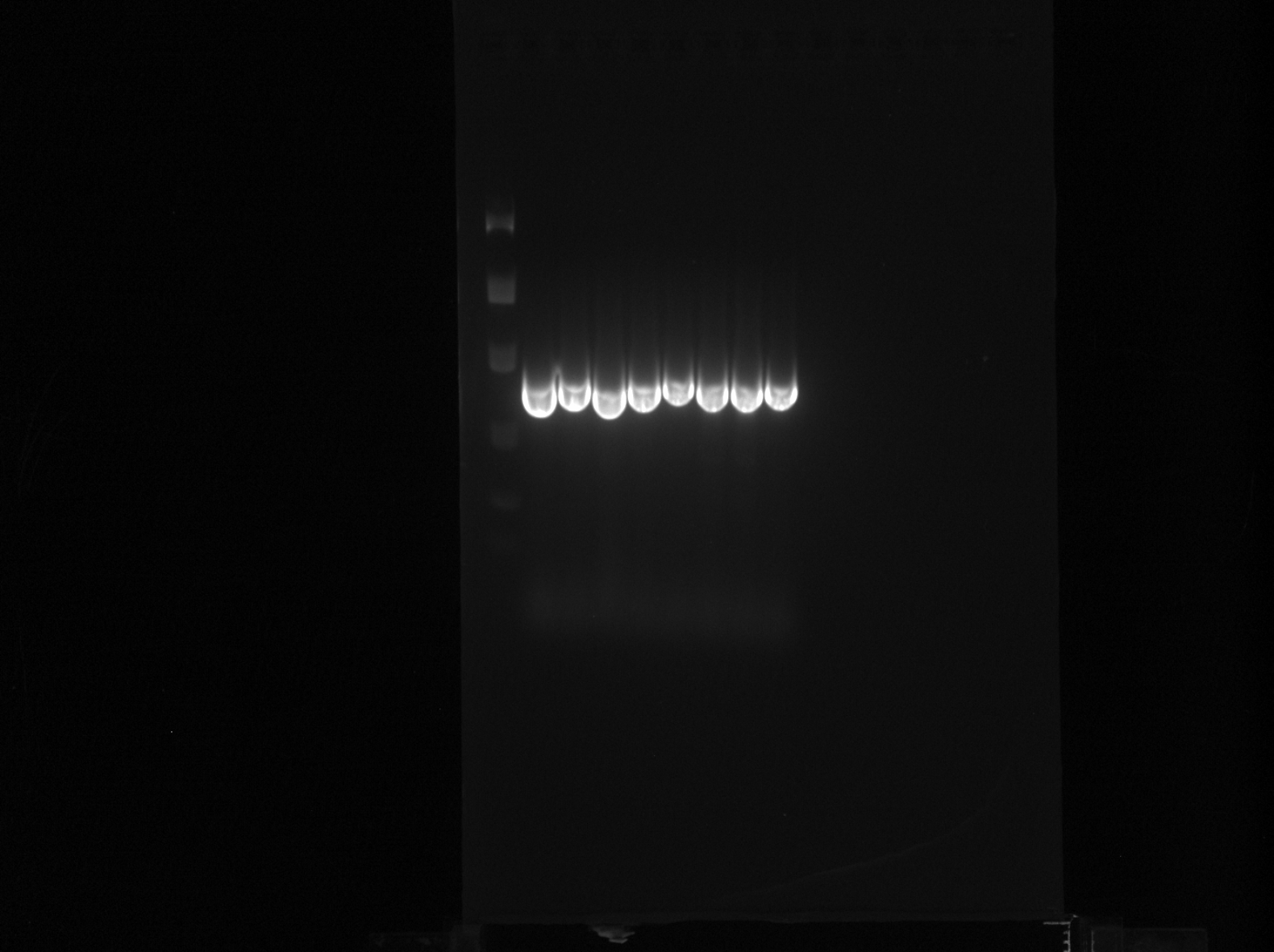

Supplement: Figure 4—source data 1. [file elife-75796-fig4-data1.zip › Figure 4-source data 1/Figure 4 original gels/Figure 4A-2.tif]

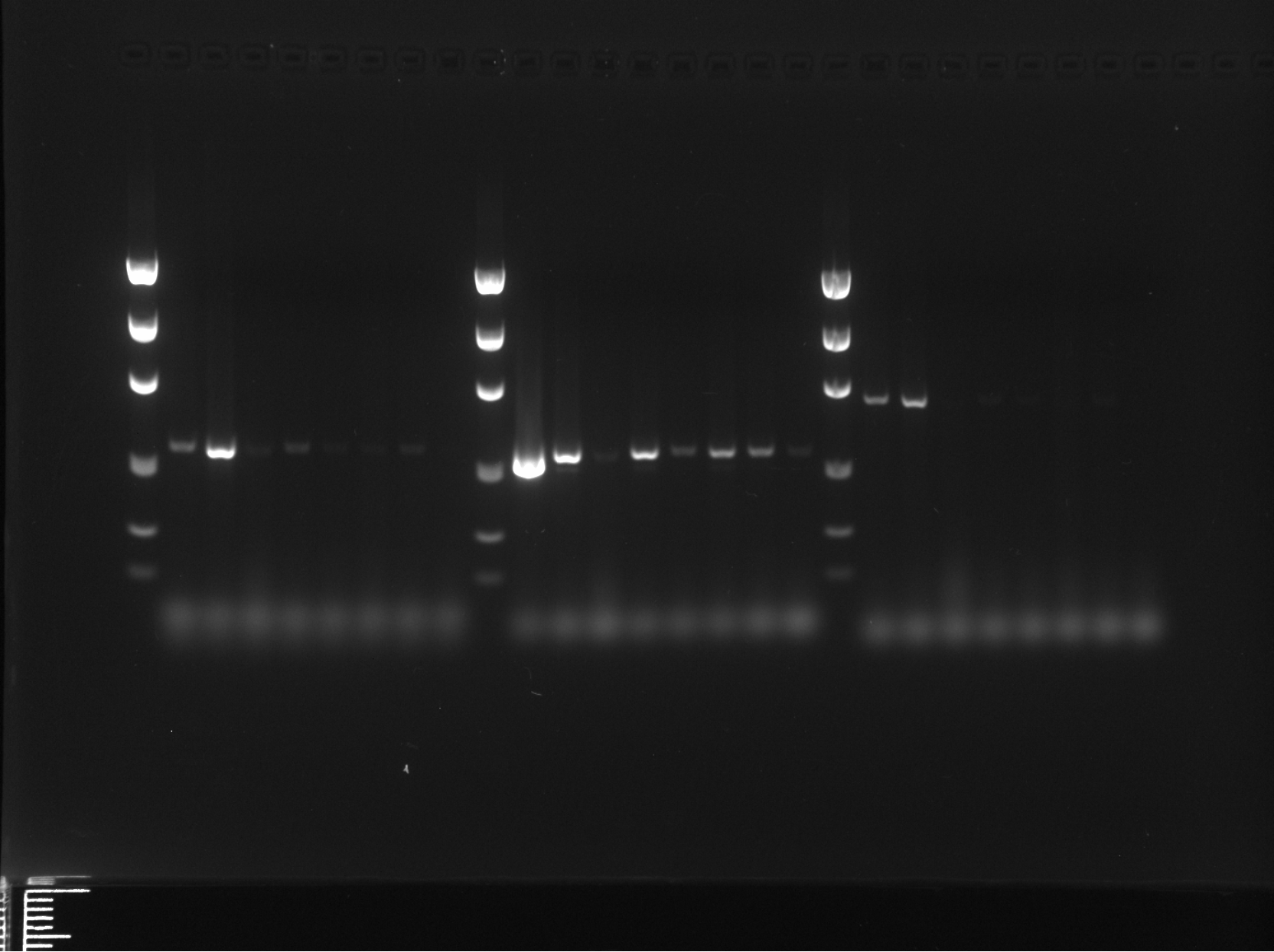

Supplement: Figure 4—source data 1. [file elife-75796-fig4-data1.zip › Figure 4-source data 1/Figure 4 original gels/Figure 4A-1.tif]

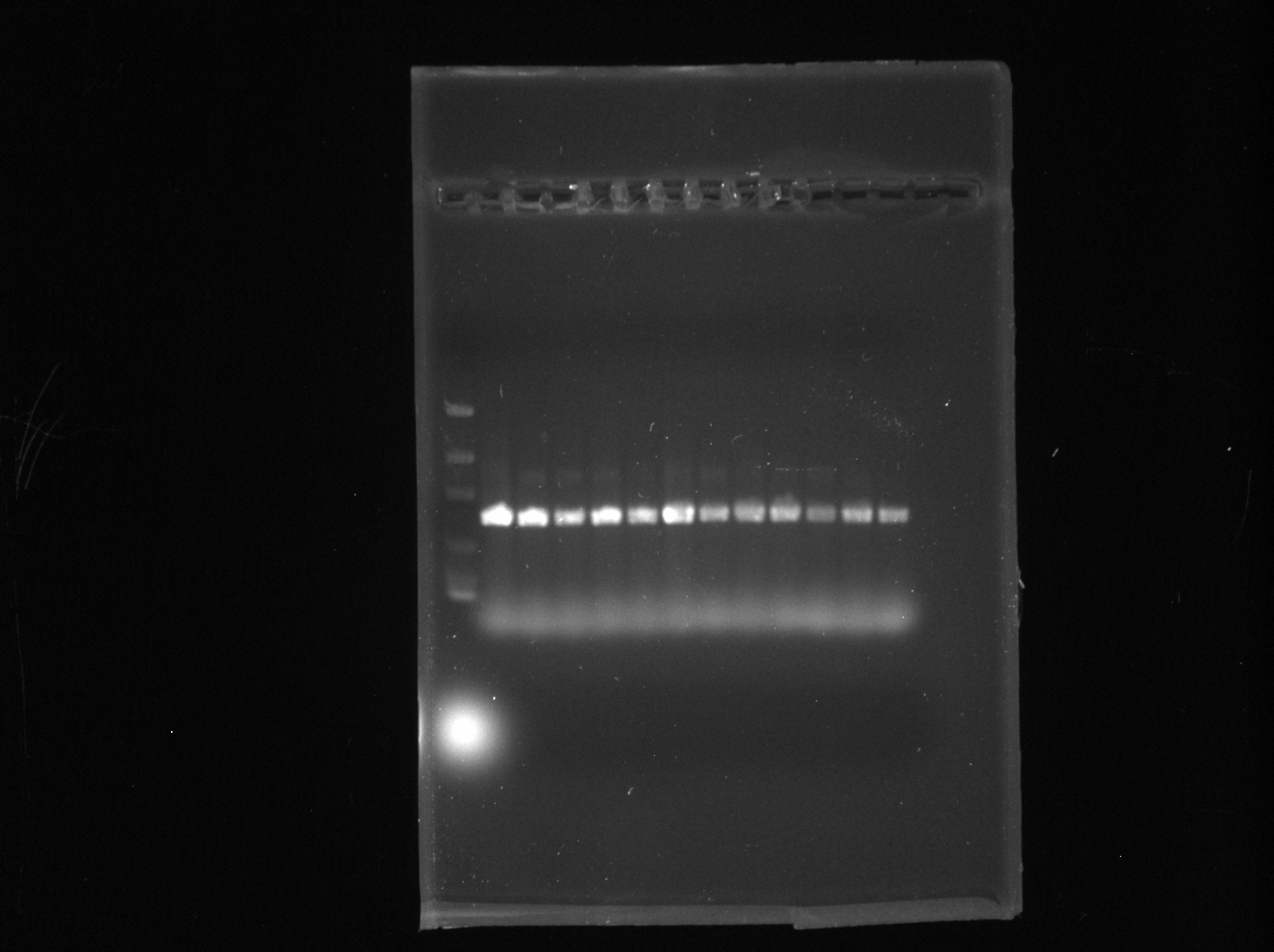

Supplement: Figure 4—source data 1. [file elife-75796-fig4-data1.zip › Figure 4-source data 1/Figure 4 original gels/Figure 4B-3.tif]

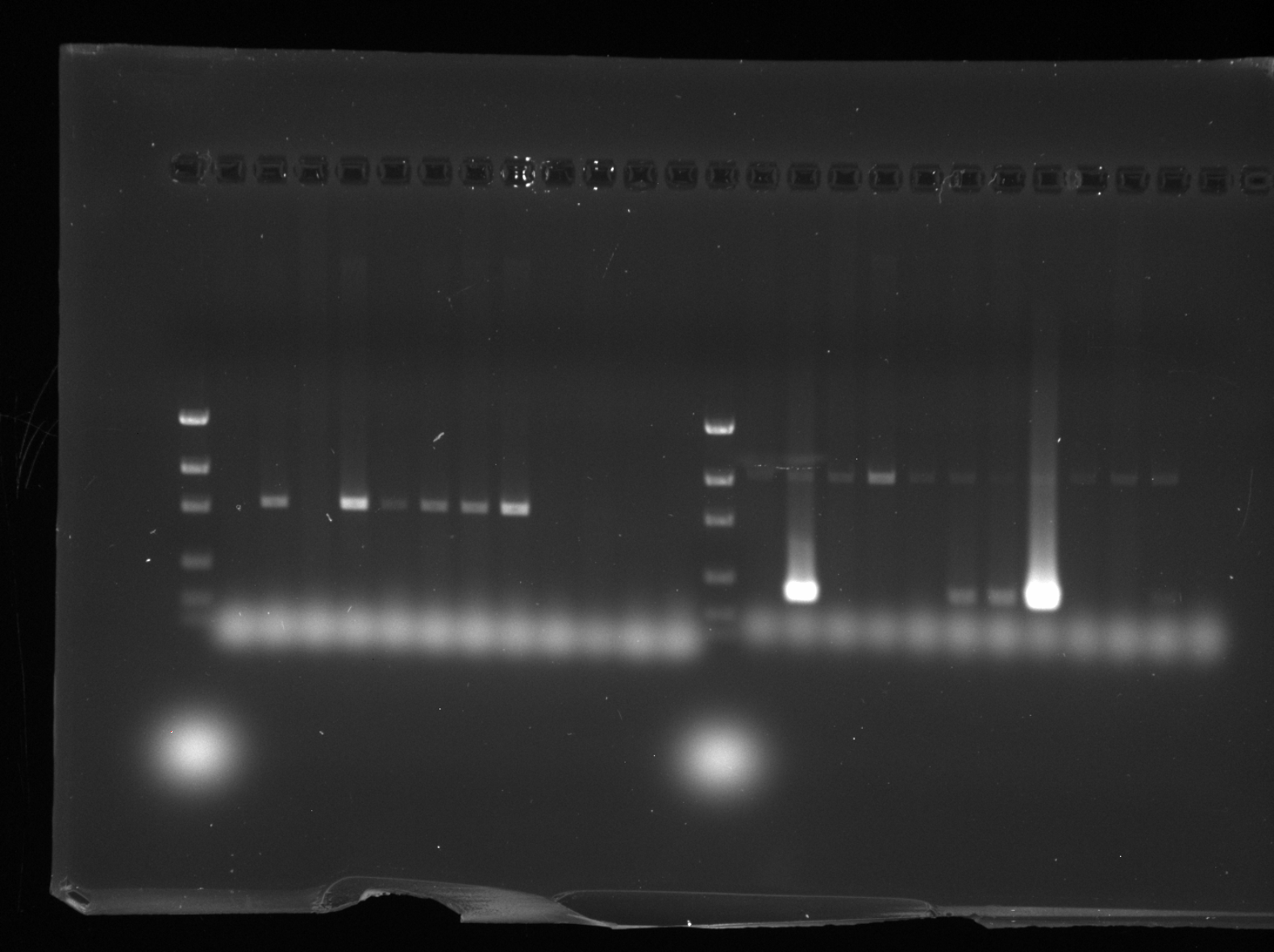

Supplement: Figure 4—source data 1. [file elife-75796-fig4-data1.zip › Figure 4-source data 1/Figure 4 original gels/Figure 4B-2.tif]

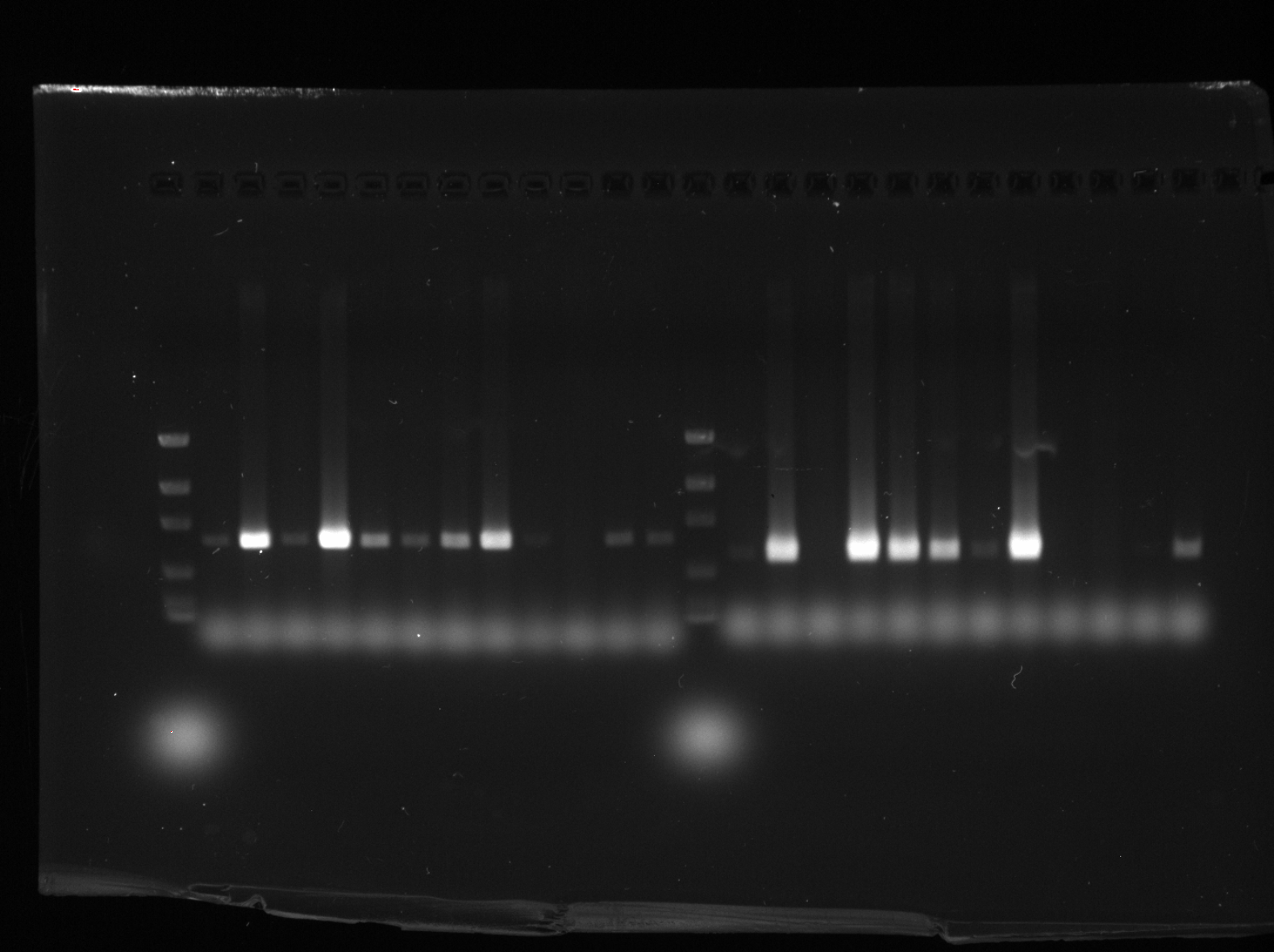

Supplement: Figure 4—source data 1. [file elife-75796-fig4-data1.zip › Figure 4-source data 1/Figure 4 original gels/Figure 4B-1.tif]
