# Supplementary material for: Activation by cleavage of the epithelial Na+ channel α and γ subunits independently coevolved with the vertebrate terrestrial migration
Source: eLife. 2022 Jan 5;11:e75796. doi: 10.7554/eLife.75796 (PMC8791634; doi:10.7554/eLife.75796)

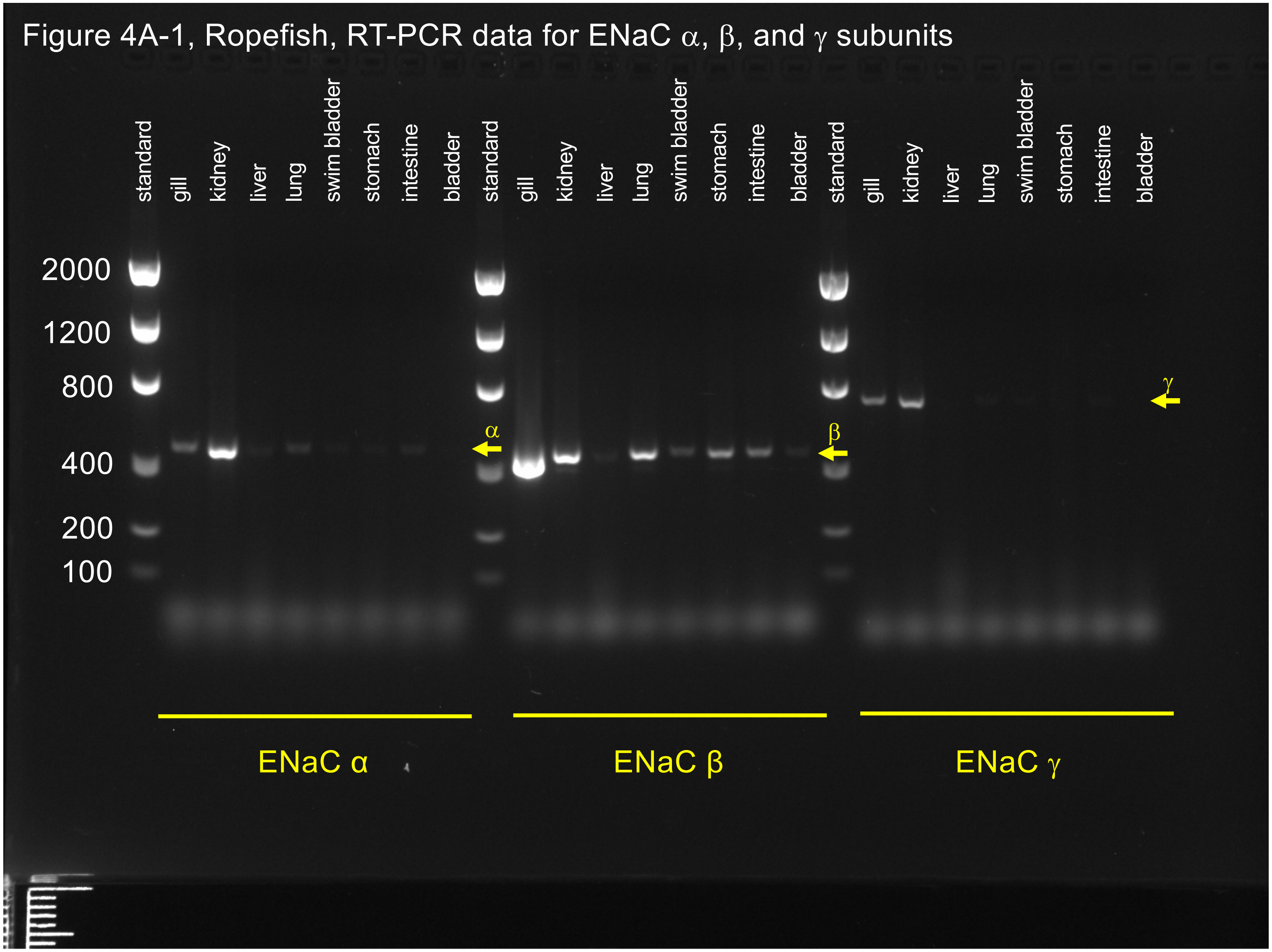

Figure 4A-1, Ropefish, RT-PCR data for ENaC α, β, and γ subunits

Figure 4A-2, Ropefish, RT-PCR data for GAPDH

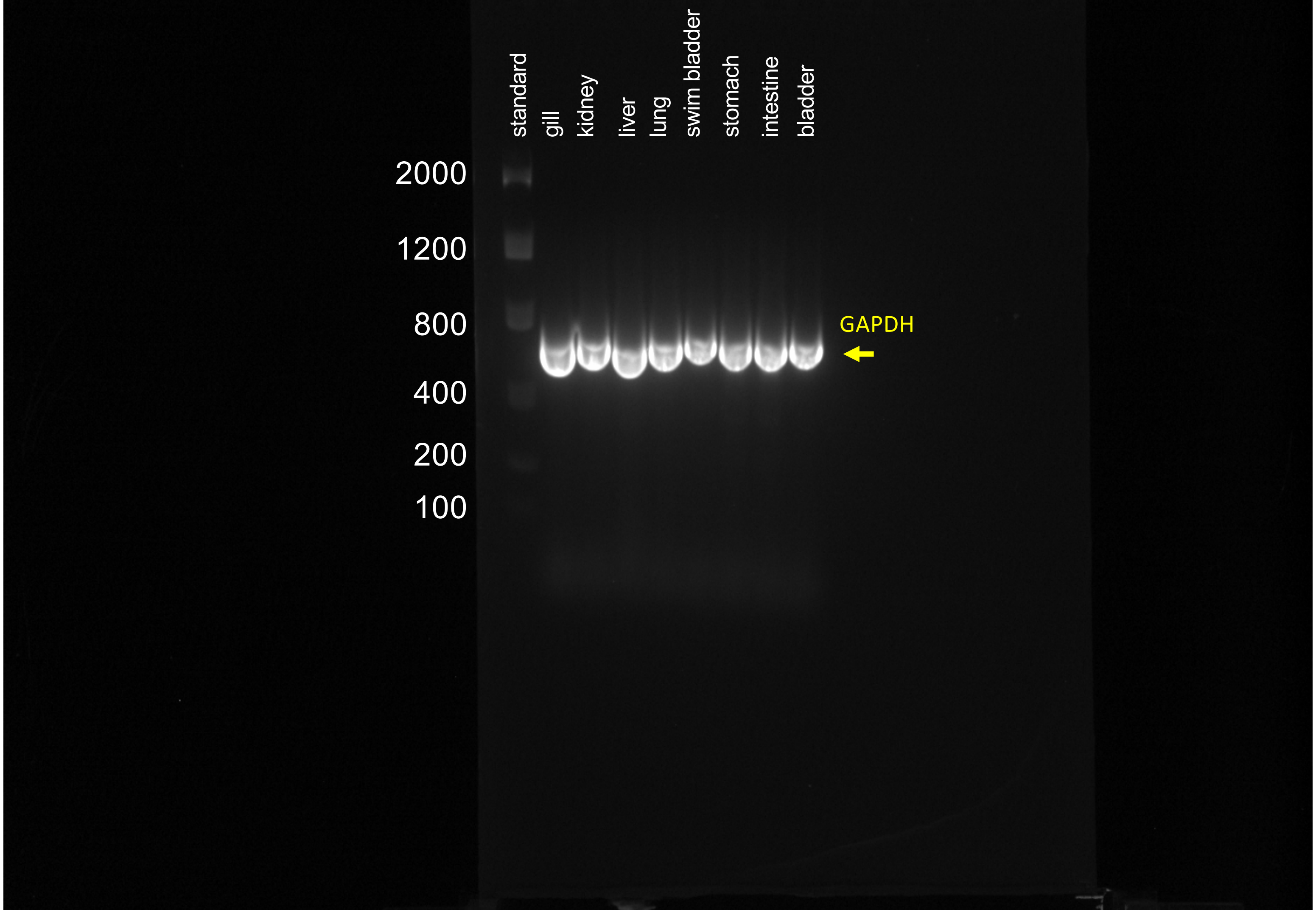

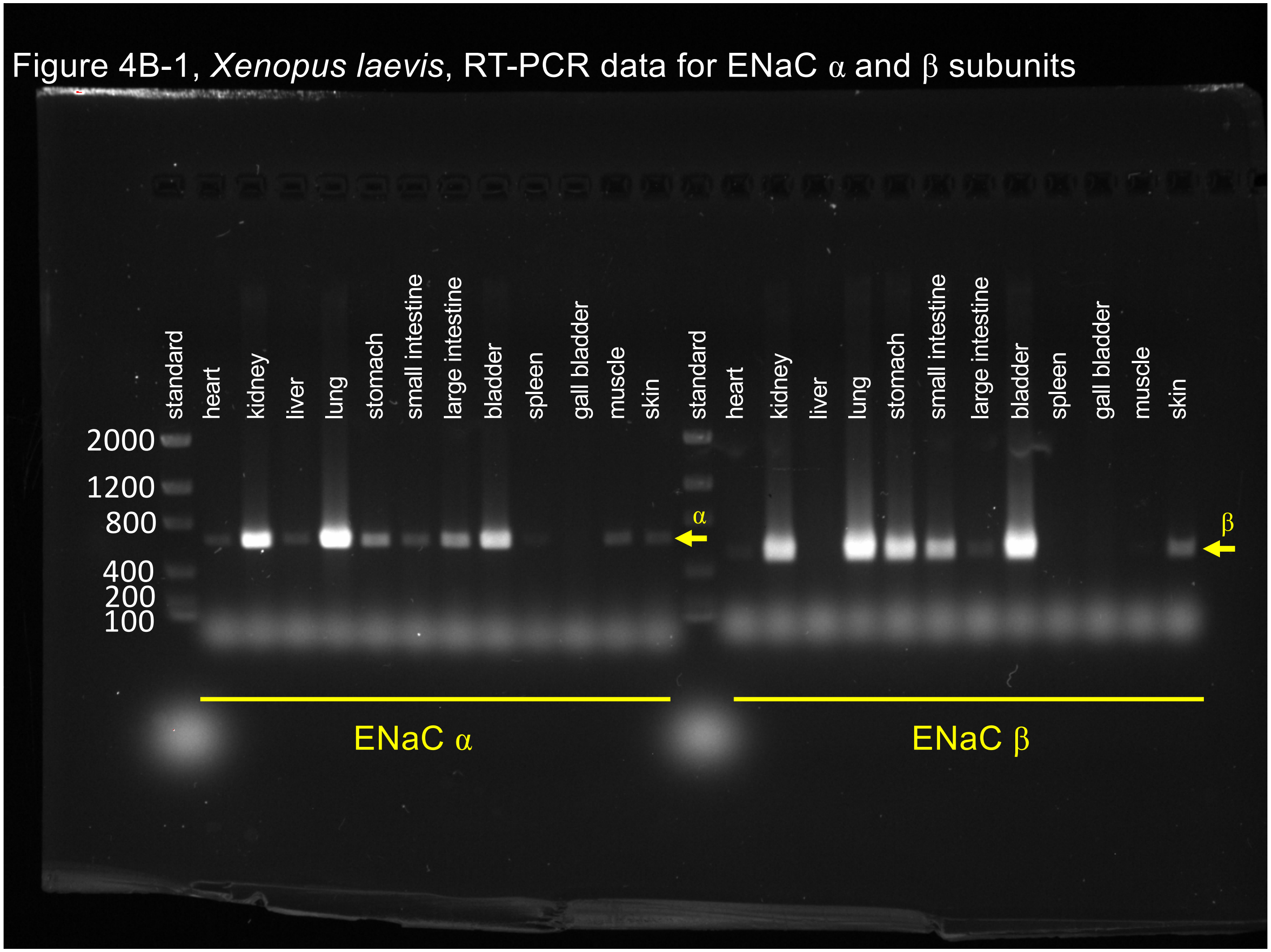
Figure 4B-1, *Xenopus laevis*, RT-PCR data for ENaC α and β subunits
standard
heart
kidney
liver
lung
stomach
small intestine
large intestine
bladder
spleen
gall bladder
muscle
skin
standard
heart
kidney
liver
lung
stomach
small intestine
large intestine
bladder
spleen
gall bladder
muscle
skin
2000
1200
800
400
200
100
α
β
ENaC α
ENaC β

Figure 4B-2, *Xenopus laevis*, RT-PCR data for ENaC γ and δ subunits

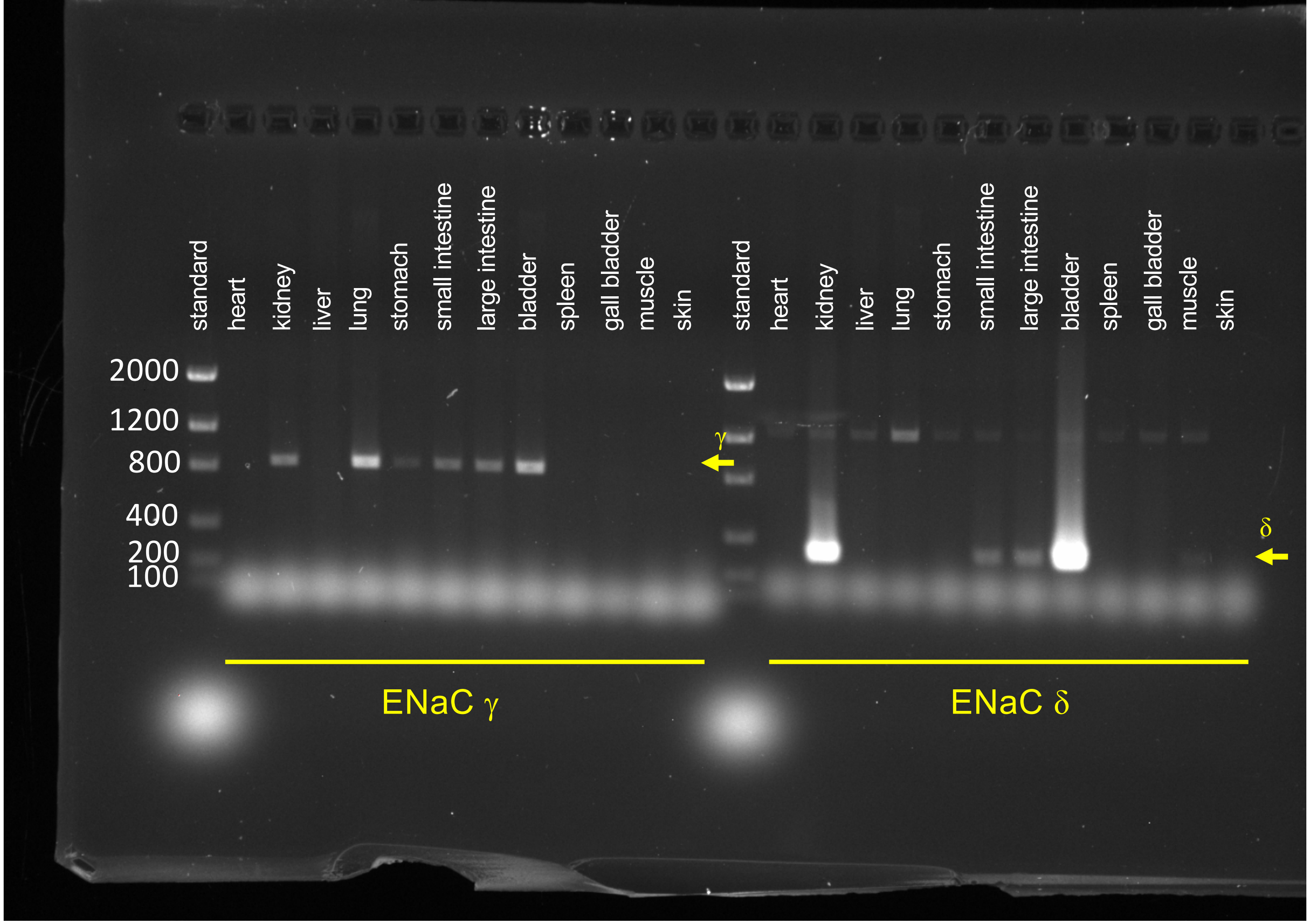

Figure 4B-3, *Xenopus laevis*, RT-PCR data for β–actin

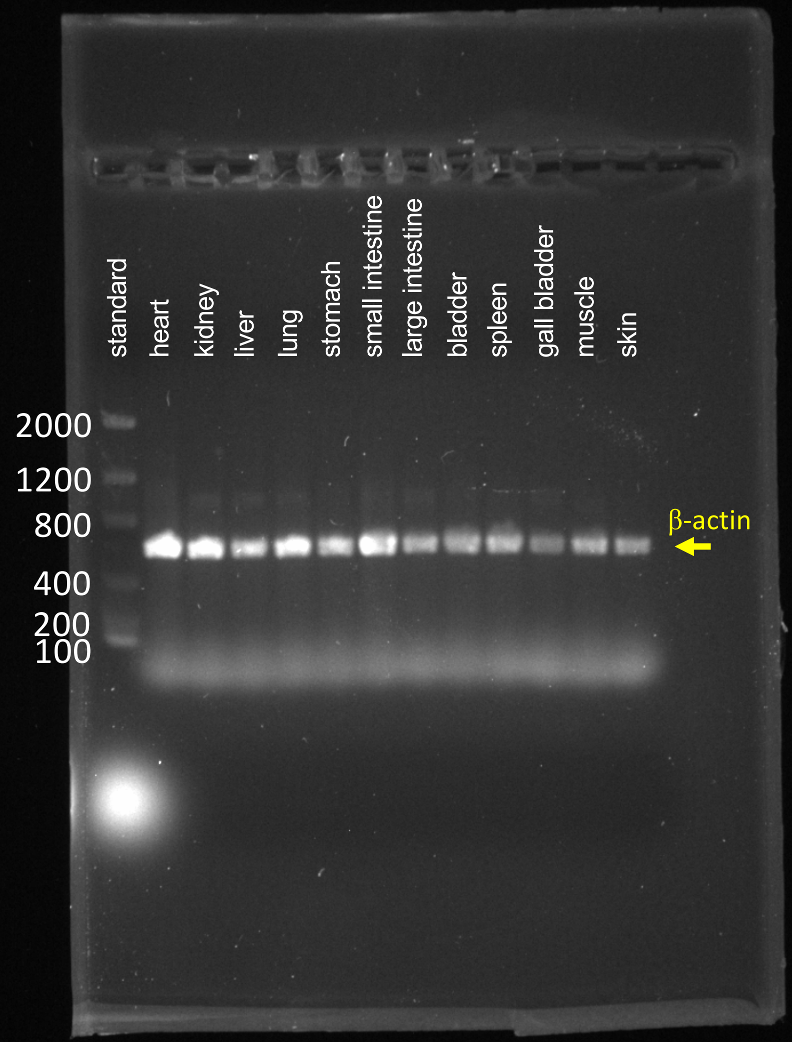

Supplement: Figure 4—source data 1. [file elife-75796-fig4-data1.zip › Figure 4-source data 1/Figure 4 uncropped labeled gels.pdf]
