# Supplementary material for: Activation by cleavage of the epithelial Na+ channel α and γ subunits independently coevolved with the vertebrate terrestrial migration
Source: eLife. 2022 Jan 5;11:e75796. doi: 10.7554/eLife.75796 (PMC8791634; doi:10.7554/eLife.75796)
Supplement: Supplementary file 1. — #Annotated as epithelial Na+ channel (ENaCα) subunit in NCBI, but named ASIC1 here on the basis of calculated phylogenetic tree in Figure 2. *Protein sequences were originally found using the BLAST tool at A*STAR (http://jlampreygenome.imcb.a-star.edu.sg/ and http://esharkgenome.imcb.a-star.edu.sg/), but were no longer available at the time of publication. Coding sequences are available at the accession numbers shown at NCBI. [file elife-75796-supp1.docx]

**Supplementary file 1.**

| Species Name | Common Name | Protein | Accession Number | Database |
| --- | --- | --- | --- | --- |
| *Callorhinchus milii* | Elephant Shark | ASIC1 | XP_007884967.1 | NCBI |
| *Lampetra fluviatilis* | European River Lamprey | ASIC1 | AAY28983.1 | NCBI |
| *Latimeria chalumnae* | Coelacanth | ASIC1 | XP_006007803.1 | NCBI |
| *Notothenia coriiceps* | Black Rock Cod | ASIC1 | XP_010778727.1 | NCBI |
| *Oryzias latipes* | Japanese Medaka | ASIC1 | XP_004068864.1 | NCBI |
| *Ictalurus punctatus* | Catfish | ASIC1^#^ | XP_017320908.1 | NCBI |
| *Bos taurus* | Cow | ENaC α | NP_777023.1 | NCBI |
| *Callorhinchus milii* | Elephant Shark | ENaC α | JW872093.1* | NCBI |
| *Chrysemys picta bellii* | Western Painted Turtle | ENaC α | XP_005291372.1 | NCBI |
| *Gallus gallus* | Chicken | ENaC α | NP_990476.2 | NCBI |
| *Homo sapiens* | Human | ENaC α | NP_001029.1 | NCBI |
| *Hynobius nigrescens* | Sendai Salamander | ENaC α | BAI66492.2 | NCBI |
| *Latimeria chalumnae* | Coelacanth | ENaC α | H3AJ42 | UniProt |
| *Lethenteron camtschaticum* | Japanese Lamprey | ENaC α | APJL01005293.1* | NCBI |
| *Neoceratodus forsteri* | Australian Lungfish | ENaC α | H1AFJ5.1 | NCBI |
| *Petromyzon marinus* | Sea Lamprey | ENaC α | S4RTA3 | UniProt |
| *Protopterus annectens* | West African Lungfish | ENaC α | BAO27802.1 | NCBI |
| *Xenopus laevis* | African Clawed Frog | ENaC α | NP_001081392.1 | NCBI |
| *Erpetoichthys calabaricus* | Ropefish | ENaC α | XP_028666006.1 | NCBI |
| *Bos taurus* | Cow | ENaC β | NP_001091544.1 | NCBI |
| *Callorhinchus milii* | Elephant Shark | ENaC β | XP_007903981.1 | NCBI |
| *Chrysemys picta bellii* | Western Painted Turtle | ENaC β | XP_005288958.2 | NCBI |
| *Gallus gallus* | Chicken | ENaC β | XP_015149982.1 | NCBI |
| *Homo sapiens* | Human | ENaC β | NP_000327.2 | NCBI |
| *Latimeria chalumnae* | Coelacanth | ENaC β | H3AVV2 | UniProt |
| *Lethenteron camtschaticum* | Japanese Lamprey | ENaC β | APJL01036227.1* | NCBI |
| *Neoceratodus forsteri* | Australian Lungfish | ENaC β | H1AFJ6.1 | NCBI |
| *Petromyzon marinus* | Sea Lamprey | ENaC β | S4RY81 | UniProt |
| *Protopterus annectens* | West African Lungfish | ENaC β | BAO27803.1 | NCBI |
| *Xenopus laevis* | African Clawed Frog | ENaC β | P51169 | NCBI |
| *Erpetoichthys calabaricus* | Ropefish | ENaC β | XP_028670289.1 | NCBI |

**Supplementary file 1** (continued)

| Species Name | Common Name | Protein | Accession Number | Database |
| --- | --- | --- | --- | --- |
| *Bos taurus* | Cow | ENaC δ | XP_005217258.1 | NCBI |
| *Chrysemys picta bellii* | Western Painted Turtle | ENaC δ | XP_005293087.1 | NCBI |
| *Gallus gallus* | Chicken | ENaC δ | XP_004947475.1 | NCBI |
| *Homo sapiens* | Human | ENaC δ | AAI25075.1 | NCBI |
| *Latimeria chalumnae* | Coelacanth | ENaC δ | H3BHF6 | UniProt |
| *Xenopus laevis* | African Clawed Frog | ENaC δ | NP_001082645.1 | NCBI |
| *Bos taurus* | Cow | ENaC γ | NP_001180103.1 | NCBI |
| *Callorhinchus milii* | Elephant Shark | ENaC γ | XP_007903982.1 | NCBI |
| *Chrysemys picta bellii* | Western Painted Turtle | ENaC γ | XP_008163042.1 | NCBI |
| *Gallus gallus* | Chicken | ENaC γ | XP_015149986.1 | NCBI |
| *Homo sapiens* | Human | ENaC γ | NP_001030.2 | NCBI |
| *Latimeria chalumnae* | Coelacanth | ENaC γ | H3AU95 | UniProt |
| *Lethenteron camtschaticum* | Japanese Lamprey | ENaC γ | APJL01036229.1* | NCBI |
| *Neoceratodus forsteri* | Australian Lungfish | ENaC γ | H1AFJ7.1 | NCBI |
| *Petromyzon marinus* | Sea Lamprey | ENaC γ | S4RK61 | UniProt |
| *Protopterus annectens* | West African Lungfish | ENaC γ | BAO27804.1 | NCBI |
| *Xenopus laevis* | African Clawed Frog | ENaC γ | NP_001079123.1 | NCBI |
| *Erpetoichthys calabaricus* | Ropefish | ENaC γ | XP_028670288.1 | NCBI |
| *Lepisosteus oculatus* | Spotted Gar | γ-like | XP_006632023.1 | NCBI |
| *Scleropages formosus* | Asian Arowana | γ-like | XP_018588349.1 | NCBI |
| *Branchiostoma belcheri* | Lancelet | γ-like | XP_019619098.1 | NCBI |
| *Branchiostoma belcheri* | Lancelet | α-like | XP_019623744.1 | NCBI |
