# Supplementary material for: Activation by cleavage of the epithelial Na+ channel α and γ subunits independently coevolved with the vertebrate terrestrial migration
Source: eLife. 2022 Jan 5;11:e75796. doi: 10.7554/eLife.75796 (PMC8791634; doi:10.7554/eLife.75796)
Supplement: Supplementary file 2. — 1ML, maximimum likelihood run. All ML runs included the commands: MLTries 10,000; ScaleTrees 1000. 2Markov chain Monte Carlo run. All MCMC runs included the commands: ScaleTrees 1000; Stones 100, 10,000. 3Restricts reverse rate for trait 1–0. 4Restricts trait one forward rates to be independent of trait 2, and trait 1 reverse rates to 0. 5Restricts the equivalent sites 1 and 2 rates to be equal. 6Restricts the equivalent sites 1 and 2 rates to be equal, but dependent on the status of the other site. 7The AddTag and Fossil commands select and restrict a node given by the most recent common ancestor of the proteins specified. Nodes 1, 2, 3, and 4 in Figure 2 are specified in commands as ‘ENaC’, ‘bg’, ‘alpha’, and ‘ad’, respectively. [file elife-75796-supp3.docx]

**Supplementary file 2.**

| # | Trait 1 | | Trait 2 | | Model | | Method | | | Commands | |
| --- | --- | --- | --- | --- | --- | --- | --- | --- | --- | --- | --- |
| 1 | Terrestrial  Lungs | | Site 1  Site 2  PY  Lungs | | Discrete:Independent | | ML^1^ | | | Res beta1 0^3^ | |
| 2 | Terrestrial  Lungs | | Site 1  Site 2  PY  Lungs | | Discrete:Dependent | | ML | | | Res q24 q13^4^  Res q31 q42 0 | |
| 3 | Site 1 | | Site 2 | | Discrete:Independent | | ML | | | Res alpha1 alpha2^5^  Res beta1 beta2 | |
| 4 | Site 1 | | Site 2 | | Discrete:Dependent | | ML | | | Res q12 q13^6^  Res q21 q31  Res q24 q34  Res q42 q43 | |
| 5 | Site 1 | | Site 2 | | Discrete:Dependent  *unrestricted ancestral nodes* | | MCMC^2^ | | | Commands from #4  Prior q34 exp 0.001  Prior q43 exp 0.001 | |
| 6 | Site 1 | | Site 2 | | Discrete:Dependent  *Trait 1 in ancestral nodes restricted to reflect divergent evolutionary model* | | MCMC | | | Commands from #7  AddTag TENaC Cow_alpha Cow_beta  Fossil ENaC TENaC 15  AddTag Tbg Cow_beta Cow_gamma  Fossil bg Tbg 15  AddTag Talpha Cow_alpha SLamprey_alpha  Fossil alpha Talpha 15  AddTag Tad Cow_alpha Cow_delta  Fossil ad Tad 15 | |
| 7 | Site 1 | | Site 2 | | Discrete:Dependent  *Trait 2 in ancestral nodes restricted to reflect divergent evolutionary model* | | MCMC | | | Commands from #7  AddTag TENaC Cow_alpha Cow_beta  Fossil ENaC TENaC 14  AddTag Tbg Cow_beta Cow_gamma  Fossil bg Tbg 14  AddTag Talpha Cow_alpha SLamprey_alpha  Fossil alpha Talpha 14  AddTag Tad Cow_alpha Cow_delta  Fossil ad Tad 14 | |
| 8 | | Site 1 | | Site 2 | | Discrete:Dependent  *Trait 1 in ancestral node 1 restricted to reflect convergent evolutionary model* | | MCMC | commands from #7  AddTag TENaC Cow_alpha Cow_beta  Fossil ENaC TENaC 10  AddTag Tbg Cow_beta Cow_gamma  AddMRCA bg Tbg  AddTag Talpha Cow_alpha SLamprey_alpha  AddMRCA alpha Talpha  AddTag Tad Cow_alpha Cow_delta  AddMRCA ad Tad | |  |
| 9 | | Site 1 | | Site 2 | | Discrete:Dependent  *Trait 2 in ancestral node 1 restricted to reflect convergent evolutionary model* | | MCMC | commands from #7  AddTag TENaC Cow_alpha Cow_beta  Fossil ENaC TENaC 11  AddTag Tbg Cow_beta Cow_gamma  AddMRCA bg Tbg  AddTag Talpha Cow_alpha SLamprey_alpha  AddMRCA alpha Talpha  AddTag Tad Cow_alpha Cow_delta  AddMRCA ad Tad | |  |
| 10 | | Terrestrial | | PY | | Discrete:Independent  *Unrestricted ancestral nodes* | | MCMC | Commands from #1  PriorAll exp 0.001 | |  |
| 11 | | Terrestrial | | PY | | Discrete:Independent  *Trait 2 in ancestral nodes restricted to reflect divergent evolutionary model* | | MCMC | Commands from #10  AddTag TENaC Cow_alpha Cow_beta  Fossil ENaC TENaC 14  AddTag Tbg Cow_beta Cow_gamma  Fossil bg Tbg 14  AddTag Talpha Cow_alpha SLamprey_alpha  Fossil alpha Talpha 14  AddTag Tad Cow_alpha Cow_delta  Fossil ad Tad 14 | |  |
| 12 | | Terrestrial | | PY | | Discrete:Independent  *Trait 2 in node 1 restricted to reflect convergent evolutionary model* | | MCMC | Commands from #10  AddTag TENaC Cow_alpha Cow_beta  Fossil ENaC TENaC 11 | |  |
| 13 | | Terrestrial | | PY | | Discrete:Independent  *Trait 2 in node 2 restricted to reflect convergent evolutionary model* | | MCMC | Commands from #10  AddTag Tbg Cow_beta Cow_gamma  Fossil bg Tbg 11 | |  |

| 14 | Terrestrial | PY | Discrete:Independent  *Trait 2 in node 3 restricted to reflect convergent evolutionary model* | MCMC | Commands from #10  AddTag Talpha Cow_alpha SLamprey_alpha  Fossil alpha Talpha 11 |
| --- | --- | --- | --- | --- | --- |
| 15 | Terrestrial | PY | Discrete:Independent  *Trait 2 in node 4 restricted to reflect convergent evolutionary model* | MCMC | Commands from #10  AddTag Tad Cow_alpha Cow_delta  Fossil ad Tad 11 |
