# Supplementary material for: Activation by cleavage of the epithelial Na+ channel α and γ subunits independently coevolved with the vertebrate terrestrial migration
Source: eLife. 2022 Jan 5;11:e75796. doi: 10.7554/eLife.75796 (PMC8791634; doi:10.7554/eLife.75796)
Supplement: Supplementary file 3. [file elife-75796-supp4.docx]

**Supplementary file 3.**

| Target | Primer sequence (5'–3') | | Amplicon (bp) |
| --- | --- | --- | --- |
| *Erpetoichthys calabaricus* | | | |
| ENaC α | forward reverse | ACATATTGCCTGGGGGCAC TTAGGCTCTTGGTGTCCATCTG | 474 |
| ENaC β | forward reverse | CCGGAATGGGTGTACTGCTA AAGGAGGAGGCCCCATGTAT | 469 |
| ENaC γ | forward reverse | AGCCGAACAACGAGCATGTA AGTTGCAAAGCTGGAAGCCT | 725 |
| GAPDH | forward reverse | TGAAAAGGCCTCTGCTCACC AGGTTTAACTGCGTCAGGGG | 734 |
| *Xenopus laevis* | | | |
| ENaC α | forward reverse | ACAGAGTGAGCCAGGATTGG AATTAACAGCTCCAGGTGGCA | 628 |
| ENaC β | forward reverse | CACATACCGCCGGCTCACT CGGCTTAACCCGTGAGTATTTGA | 515 |
| ENaC γ | forward reverse | GTCAGATCTCTGGGACAGATCCA GTTCCGAGCATCACAGGACA | 847 |
| ENaC δ | forward reverse | ACCACTTTCTGGCTTGTGCT CCTCCATTGACTTGGCCTGT | 320 |
| β-actin | forward reverse | GCCCGCATAGAAAGGAGACA GTCTGTCAGGTCACGTCCAG | 610 |
