# Supplementary material for: Activation by cleavage of the epithelial Na+ channel α and γ subunits independently coevolved with the vertebrate terrestrial migration
Source: eLife. 2022 Jan 5;11:e75796. doi: 10.7554/eLife.75796 (PMC8791634; doi:10.7554/eLife.75796)
Supplement: Supplementary file 4. [file elife-75796-supp5.docx]

**Supplementary file 4.**

#Nexus

Begin Trees;

Translate

1 Frog_alpha,

2 Frog_beta,

3 Frog_delta,

4 Frog_gamma,

5 Alungfish_alpha,

6 Alungfish_beta,

7 Alungfish_gamma,

8 BCod_ASIC1,

9 Arowana_g_like,

10 Lancelet_a_like,

11 Catfish_alpha,

12 Chicken_alpha,

13 Chicken_beta,

14 Chicken_delta,

15 Chicken_gamma,

16 Coelacanth_ASIC1,

17 Coelacanth_alpha,

18 Coelacanth_beta,

19 Coelacanth_delta,

20 Coelacanth_gamma,

21 Cow_alpha,

22 Cow_beta,

23 Cow_delta,

24 Cow_gamma,

25 Eshark_alpha,

26 Eshark_ASIC1,

27 Eshark_beta,

28 Eshark_gamma,

29 Elamprey_ASIC1,

30 Human_alpha,

31 Human_beta,

32 Human_delta,

33 Human_gamma,

34 JLamprey_alpha,

35 JLamprey_beta,

36 JLamprey_gamma,

37 JMedaka_ASIC1,

38 Lancelet_g_like,

39 Ropefish_beta,

40 Ropefish_gamma,

41 Ropefish_alpha,

42 SLamprey_alpha,

43 SLamprey_beta,

44 SLamprey_gamma,

45 Salamander_alpha,

46 SpottedGar_g_like,

47 WLungfish_alpha,

48 WLungfish_beta,

49 WLungfish_gamma,

50 Turtle_alpha,

51 Turtle_beta,

52 Turtle_delta,

53 Turtle_gamma

;

Tree tree_1 = ((((10:0.240154,38:0.400628):0.26525,(9:0.332645,46:0.205856):0.672235):0.132642,(29:0.024507,

((8:0.020914,37:0.026452):0.020569,(26:0.041216,(11:0.054532,16:0.022955):0.024701):1E-06):0.059287):

0.917353):0.065548,(((34:0.004582,42:0.050101):0.445435,((19:0.339745,(3:0.354015,((52:0.082886,14:

0.103927):0.085846,(32:0.199417,23:0.044511):0.204):0.090769):0.102711):0.154028,(25:0.80082,(41:

0.214639,((5:0.090541,47:0.111692):0.0775,(17:0.249053,(1:0.178596,(45:0.221607,((50:0.067304,12:

0.056789):0.076495,(30:0.021723,21:0.082526):0.142622):0.057806):0.056807):0.093321):0.04201):0.04742):

0.02314):0.039558):0.032187):0.182706,(((36:0.007161,44:0.030747):0.356102,((28:0.247241,40:0.284502):

0.040389,(20:0.138309,((7:0.113037,49:0.120798):0.151714,(4:0.176153,((53:0.029715,15:0.054405):

0.057263,(33:0.014233,24:0.049296):0.128043):0.069185):0.046522):0.012495):0.042939):0.250399):0.186618,((35:0.037746,43:1E-06):0.193114,(39:0.259873,((18:0.135812,(27:0.296282,(6:0.093056,48:0.073352):

0.216967):0.012272):0.028046,(2:0.20256,((51:0.013166,13:0.029852):0.064836,(31:0.037238,22:0.057629):

0.087265):0.073248):0.067358):0.038976):0.116599):0.130724):0.134782):0.147799);

End;
